# Harnessing Cooperative Energy Transfer and Hydrogen Atom Transfer for Direct Nitrogenations of Non‐Activated Alkanes

**DOI:** 10.1002/anie.202518795

**Published:** 2025-11-28

**Authors:** Sai Teja Kolla, Àlex Díaz‐Jiménez, Sven Trienes, Ignacio Funes‐Ardoiz, Joanna Wencel‐Delord

**Affiliations:** ^1^ Institute of Organic Chemistry JMU Würzburg Am Hubland 16 Würzburg Germany; ^2^ Institut für Organische und Biomolekulare Chemie and Wöhler Research Institute for Sustainable Chemistry (WISCh) Georg‐August‐Universität Tammannstrasse 2 Göttingen 37077 Germany; ^3^ Departamento de química Instituto de Investigación Química de la Universidad de La Rioja (IQUR) Universidad de La Rioja Madre de Dios 53 Logroño 26004 Spain; ^4^ Laboratoire d'Innovation Moléculaire et d'Applications (CNRS UMR 7042) Université de Strasbourg 25 rue de Becquerel, Strasbourg Strasbourg 67037 France

**Keywords:** Amination of alkanes, C─H functionalization of alkanes, Energy transfer, Nitrene radical, Photocatalysis

## Abstract

While important progress has been achieved recently on direct functionalization of alkanes via photocatalytic methods, direct amination of aliphatic compounds remains a great challenge. Herein we report a conceptually new strategy towards direct conversion of hydrocarbons into amines, based on in situ generation of nitrene radical. Energy transfer between thioxanthone (TXO) photocatalyst and iminoiodinane furnishes the triplet nitrene radical, able to undergo HAT reaction promoting alkane activation and subsequent C─N bond formation step. The user‐friendly nature of the metal‐free transformation, along with mild reaction conditions further highlight the sustainability of this scalable process delivering a diversity of cycloalkylamines. Combined experimental and computational mechanistic studies support the unusual reaction mechanism.

## Graphical abstract

Cooperative EnT & HAT: New solution for direct C─N coupling of unactivated alkanes. Metal‐free photocatalytic nitrogenation. Use of unactivated alkanes. Original reaction pathway.

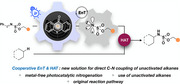

The amine functional group is omnipresent in a diversity of natural products and therefore, not surprisingly, it is also one of the most often encountered functionalities in drug design (Figure 1a).^[^ ^1^, ^2^, ^3^, ^4^, ^5^, ^6^, ^7^, ^8^ ^]^ Applications of alkyl‐amines are, however, much broader, encompassing agrochemistry, rubber industry, water treatment, and textiles. Therefore, as new structures are continuously requested for the pharmaceutical industry, and green processes are in demand for the large‐scale industrial applications, sustainable synthesis of aliphatic amines presents a great scientific challenge. While industrially aliphatic amines are typically synthesized from the corresponding alcohols or ketones,^[^ ^9^ ^]^ the design of straightforward transformations allowing direct amination of latent C─H bonds is extremely appealing.^[^ ^10^, ^11^ ^]^ However, while over the last two decades, the field of direct C─H activation has progressed tremendously,^[^ ^12^, ^13^, ^14^, ^15^, ^16^ ^]^ inner‐sphere activation of aliphatic substrates is underdeveloped (Figure 1b **, up**).^[^ ^17^, ^18^, ^19^ ^]^ In particular, direct amination of alkanes occurring via a metalation step is poorly known. The need of directing groups, combined with challenging metalation of C(sp^3^)─H bonds alongside high‐energy demanding C─N‐forming reductive eliminations, considerably limits the widespread use of this synthetic route.^[^ ^20^, ^21^, ^22^ ^]^ Rare examples of photocatalytic C─H aminations of unbiased C(sp^3^)─H bonds are also limited by the general need for dialkyl azodicarboxylate as aminating agent and formation of hydrazide derivatives.^[^ ^23^, ^24^, ^25^, ^26^, ^27^, ^28^ ^]^

**Figure 1:**
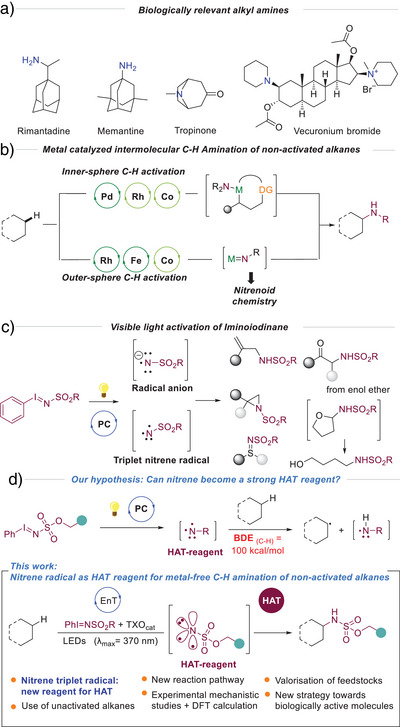
a) Selected examples of biologically relevant alkyl amines. b) Recent examples of nitrene C─H insertion into non‐activated C─H bonds via transition metal catalysis. c) Nitrene derivatives generation and subsequent reactivity via photocatalytic activation of iminoiodinanes. d) Photocatalytic intermolecular nitrene C─H amination of non‐activated hydrocarbons via energy transfer mechanism.

In contrast, outer‐sphere C─H amination is a well‐established and privileged strategy to convert latent C─H bonds into amine motifs (Figure 1b **, down**).^[^ ^29^, ^30^, ^31^, ^32^, ^33^ ^]^ Catalytic systems based on Rh(II),^[^ ^34^, ^35^, ^36^, ^37^, ^38^, ^39^, ^40^ ^]^ Fe,^[^ ^41^, ^42^, ^43^, ^44^, ^45^ ^]^ Co,^[^ ^46^, ^47^ ^]^ Cu,^[^ ^48^, ^49^, ^50^, ^51^, ^52^ ^]^ or Ag,^[^ ^53^, ^54^, ^55^ ^]^ allow typically selective cleavage of electronically activated C─H bonds at benzylic, allylic and tertiary positions. While diversity of metal catalysts can be used, the key role of nitrene species in direct amination of unbiased alkanes is evident. Therefore, development of alternative strategies to activate nitrenes under metal‐free conditions, could provide a conceptually complementary pathway toward even more sustainable C─N bond formation of simple aliphatic substrates.^[^ ^56^ ^]^ In this regard, the chemistry of iminoiodinanes under visible light irradiation has attracted significant attention as potential nitrene precursors.^[^ ^57^ ^]^ Independent studies by Powers,^[^ ^58^ ^]^ Takemoto,^[^ ^59^ ^]^ Yoon,^[^ ^60^ ^]^ Koenigs,^[^ ^61^, ^62^, ^63^ ^]^ and Codée^[^ ^64^ ^]^ demonstrated that under photocatalytic conditions or simple blue light irradiation, either direct excitation of the iminoiodinane or single‐electron transfer (SET) from the photocatalyst occurs and thus generated species are able to directly react with highly activated functionalized motifs. Accordingly, chemoselective functionalization of olefins furnishing either aziridines^[^ ^58^, ^59^, ^60^, ^61^, ^62^, ^63^, ^64^ ^]^ or allylic amines,^[^ ^62^, ^65^ ^]^ sulfilimination,^[^ ^63^, ^66^ ^]^ or amination of enol ethers^[^ ^67^ ^]^ were achieved. Besides, amination of ethers (BDE_C‐H_ of approx. 92 kcal mol^−1^) via thermal decomposition or photocatalyst‐free irradiation of iminoiodinanes followed by opening of the functionalized compounds yielding amino alcohols was described by Jurberg and König (Figure 1c).^[^ ^68^ ^]^

In clear contrast, the use of photocatalytically generated nitrenes to activate very strong, non‐activated C(sp^3^)─H bonds remains unexplored. We envisioned that if the nature of nitrene radical species is well controlled, they could be used as in situ generated strong HAT‐reagents, thus promoting the C(sp^3^)─H cleavage and subsequent amination. Herein we describe our strategy to activate iminoiodinanes, in presence of thioxanthone (TXO) as the photocatalyst, under visible light irradiation, for the amination of non‐activated alkanes (Figure 1d). In depth mechanistic studies combined with DFT calculations shed light on the unconventional mechanism of this transformation, proving that the desired reactivity is observed when an energy transfer process^[^ ^69^, ^70^, ^71^, ^72^ ^]^ occurs between the thioxanthone photocatalyst and the iminoiodinane, while the SET‐type process is unproductive.

At the outset of our experimental work, cyclohexane (**1b**) was selected as the model alkane substrate, together with iminoiodinane **2a** as the aminating agent (Figure 2). Attempts using organophotosensitizers such as BzPh, Selectfluor, 4CzlPN^[^ ^73^ ^]^ or Eosin failed as the desired cyclohexylamine derivative was observed only in trace amounts. Changing to organometallic, Ir‐ or Ru‐based photosensitizers did not allow the desired reactivity either. The first highly encouraging result was obtained in the presence of TBADT photosensitizer, and the desired product **3b** was isolated in 40% yield. Further optimization of the photosensitizer revealed that thioxanthone derivative photocatalysts are particularly active. While unsubstituted thioxanthone (TXO) furnished the desired product in 58% yield, lower reactivity was observed while using TXO derivatives bearing either electron‐donating or electron‐withdrawing functional groups. Regarding the light source, the optimal results were generated using 370 nm LED while longer wavelengths led to a progressive drop in the efficiency of this photocatalytic transformation. The reaction also turned out to be particularly sensitive to the solvent. While screening several standard organic solvents, the desired product was only formed in low yields in MTBE and EtOAc. Conversely, we observed that chlorinated solvents played a relevant role in the transformation, as high levels of reactivity were reached in dichloromethane and chloroform.

**Figure 2:**
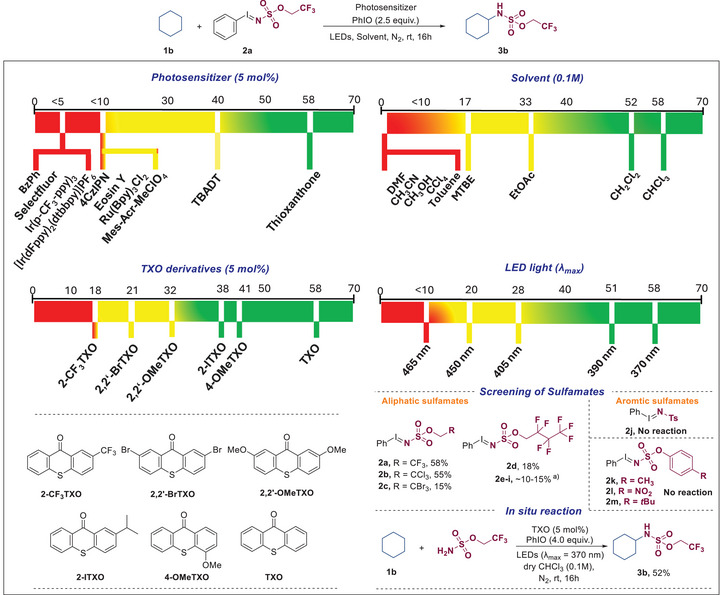
Optimization of the reaction conditions for the photocatalytic amination of non‐activated C─H bonds. ^a)^See Supporting Information.

While considering the nature of iminoiodinanes **2**, 2,2,2‐trifluoroethyl (phenyl‐λ^3^‐iodaneylidene)sulfamate (**2a**) and 2,2,2‐trichloroethyl (phenyl‐λ^3^‐iodaneylidene)sulfamate (**2b**) provided the desired products in high yields. Inferior reactivities were observed in the presence of 2,2,2‐tribromoethyl (**2c**), and heptafluorobutyl (**2d**), and (**2e‐i**) sulfamates whereas aromatic sulfamates (**2j‐m**) turned out to be incompatible with this photocatalytic reaction. While addition of the iodosobenzene is not essential for the reactivity, higher efficiency is generally observed due to the in situ regeneration of **2a**. Very importantly, the reaction can be conducted as well using 2,2,2‐trifluoroethyl sulfamate together with iodosobenzene. This in situ protocol, obviating the synthesis of **2a**, afforded the expected product in only slightly decreased yield of 52%.

With the optimized reaction conditions in hand and our mechanistic hypothesis validated, the scope of this transformation was explored, focusing on cyclic aliphatic hydrocarbons (Scheme 1a). The desired reaction could be extended toward cyclopentane (**3a**), cycloheptane (**3c**) and cyclooctane (**3d**) substrates while maintaining a similar reactivity. Methyl substituted cyclopentane also performed well, with the amination occurring preferentially at the tertiary position (**3e**). Direct amination of *cis* (**3f**) and *trans* (**3** **g**) 1,4‐dimethylcyclohexane proceeds in approximately 50% yield, regardless of the configuration of the methyl substituents. In terms of diastereoselectivity, the *cis* isomer affords the corresponding aminated product **3f** with complete selectivity, whereas the *trans* isomer leads to a d.r. of 1.2:1. Previous reports on radical addition to 1,4‐dimethylcyclohexane have shown that *cis*‐1,4‐dimethylcyclohexane reacts about twice as fast as its *trans* counterpart.^[^ ^74^ ^]^ In our case, this difference in reactivity could be attributed to a memory effect, enabling fully diastereoselective amination of *cis‐*1,4‐dimethylcyclohexane. Based on DFT calculations (Figure S15), upon HAT, we observed the formation of an adduct between the newly generated cyclohexyl‐derived radical and the protonated nitrene radical, previous to the radical recombination. In the case of the *cis* isomer a considerably higher stabilization is observed rationalizing the conservation of stereochemical information for this isomer. In contrast, the *trans* isomer exhibits a significantly lower stabilization energy, leading to a loss of stereochemical information and the formation of substantial amounts of the *cis*‐aminated product, and consequently a decreased diastereomeric ratio. Surprisingly, when norbornane was submitted to standard reaction conditions, direct amination at the secondary position was observed, selectively, furnishing **3j** in 40% isolated yield. Direct functionalization of *trans* decalin is also selective toward the C2‐secondary position, delivering **3k** in 66% yield. The adamantane scaffold is tolerated well under our reaction conditions, furnishing highly substituted adamantyl amines **3l** to **3o**. Interestingly, halogenated substituents were fully compatible with our reaction protocol. Iodo‐, bromo‐, chloro‐, or trifluoro‐ substituted cyclopentane and cyclohexane could be easily converted into the corresponding aminated compounds **3p**‐**3t** with good to moderate yields. Remarkably, these reactions are fully regioselective toward α‐secondary positions and the products obtained can be further derivatized in an easy manner (vide infra). X‐ray structure of **3t’** clearly shows that the desired direct functionalization occurs in *trans* with respect to the halogen substituent. Similarly, linear alkanes such as pentane **2u** and hexane **2v** were also compatible with the reaction protocol, and after careful isolation, the corresponding aminated products **3 u** and **3v** were obtained as mixtures of regioisomers. Interestingly, although the benzylic position is commonly highly reactive toward direct functionalizations, aromatic compounds turned out to be incompatible with our protocol, leading to shut down of the reactivity. Based on DFT calculations (Figure S13), direct HAT between TXO and benzylic substrates proceeds with a lower barrier than those related to the formation of nitrene intermediates thus inhibiting the desired reactivity.

**Scheme 1:**
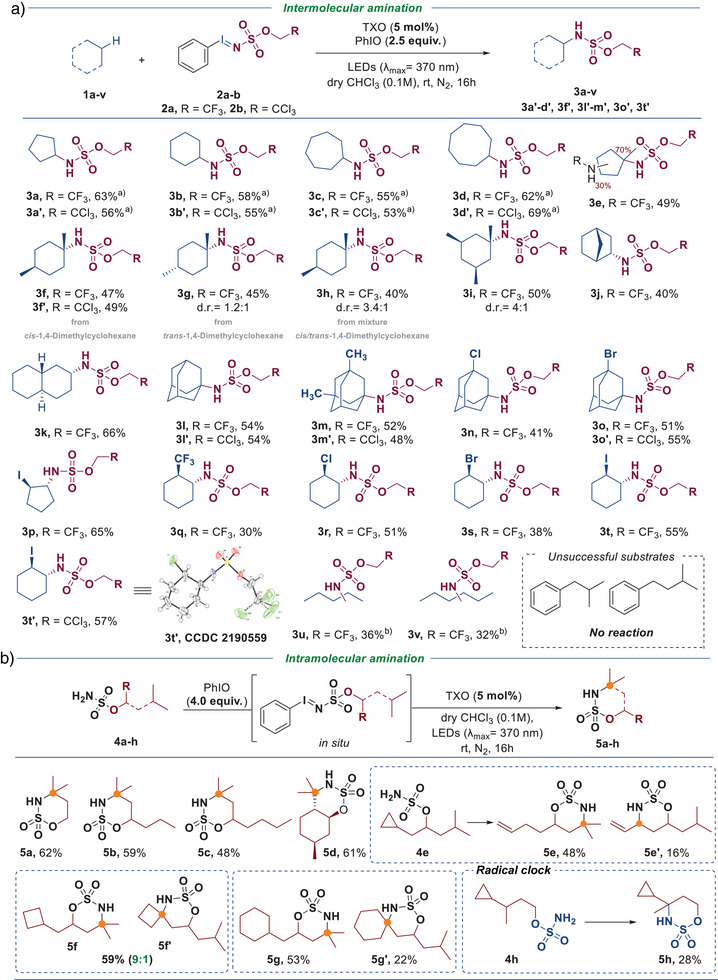
Scope of the photocatalytic amination; Reaction conditions a): **1** Iminoiodinane (0.14 mmol), **2** Cycloalkane (5.0 equiv.), Iodosobenzene (2.5 equiv.), and TXO (5.0 mol%), ^a)^10.0 equiv. of cycloalkane used, ^b)^Reaction performed at 0.5 mmol scale; Reaction conditions b): **4** Imino sulfamate (0.5 mmol), Iodosobenzene (4.0 equiv.), and thioxanthone (5.0 mol%) in dry CHCl_3_ (0.1M), irradiation with LEDs (*λ*_max_ = 370 nm, 2 x 40 W at 100% intensity) under nitrogen atmosphere at room temperature for 16h. Unless otherwise noted obtained d.r. > 20:1. TXO, Thioxanthone.

Encouraged by the interesting results obtained in the direct hydrocarbon amination, our attention focused, subsequently, on intramolecular reactions (Scheme 1b). As expected, the reaction occurs selectively at the tertiary position, delivering the cyclized products **5a–5c** in synthetically useful yields. Direct amination of the menthol derived sulfamate proceeded smoothly as well, delivering the product **5d** in 61% yield. Ring opening of the cyclopropyl group in **4e** occurs upon formation of a cyclopropylcarbinyl radical, which rapidly rearranges to a homoallylic radical owing to strain release and radical stabilization, affording the rearranged oxathiazinane products **5e** and **5e′**. When substrates bearing two tertiary positions are used, products are obtained as regioisomeric mixtures. The cyclobutane‐derived compound **5f** showed high regioselectivity (9:1), whereas the cyclohexane‐substituted sulfamate gave only moderate selectivity, producing regioisomers **5** **g** and **5g′**. Finally, substrate **4** **h**, bearing a cyclopropyl unit, was subjected to the reaction conditions as a radical clock experiment. Formation of **5** **h** was observed as the major product, with only trace amounts of ring‐opened species detected by ^1^H NMR (See Supporting Information). This result suggests that the formation of a freely diffusing radical at this position is unlikely and is instead consistent with a short‐lived radical intermediate.

With the new protocol for photocatalytic amination confirmed, our attention focused on the mechanistic studies to corroborate our initially hypothesized reaction pathway (Figure 1d). Preliminary mechanistic experimental work aimed at demonstrating the radical pathway. Therefore, the reaction was carried out in the presence of several radical trapping reagents (Figure 3a). Addition of TEMPO completely suppressed the reactivity, and the presence of the corresponding cyclohexane‐TEMPO intermediate was detected by HRMS. The use of BHT as the radical trap reduced significantly the reaction efficiency. These results clearly point toward the involvement of radical species in our transformation. Coherently, only a trace amount of product was observed under air. Very interestingly, in the presence of 2,5‐dimethylhexa‐2,4‐diene, generally acknowledged triplet energy transfer quencher,^[^ ^75^ ^]^ complete shutdown of the reactivity was witnessed. This result clearly points out toward unusual iminoiodinane activation, thus supporting our initial mechanistic hypothesis. The second set of experiments was devoted to understanding how the light irradiation promotes the desired reactivity. Spectroscopic investigations confirmed that TXO absorbs light at 370 nm, whereas the absorption of both, **2a** and PhIO, is very limited (Figure 3b), which was further confirmed by a Stern–Volmer quenching study (Figure 3c). Besides, on/off experiments point toward a light‐mediated catalytic cycle and constant light irradiation is crucial to warrant productive reaction outcome (Figure S10).

**Figure 3:**
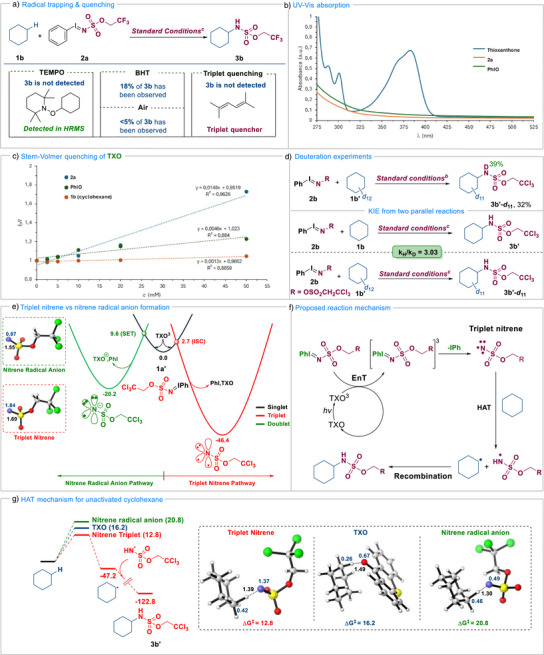
a) Radical trapping and quenching experiments. ^a)^5.0 equiv. of TEMPO/ 1.0 equiv. of BHT/ 1.0 equiv. of 2,5‐dimethylhexa‐2,4‐diene in standard conditions. b) UV–Vis absorption spectrometry. c) Stern–Volmer luminescence quenching experiment. d) Deuteration experiments. ^b)^10.0 equiv. of cyclohexane‐*d_12_* under standard conditions. ^c)^10.0 equiv. of cyclohexane and cyclohexane‐*d_12_* for two parallel reactions under standard conditions. e) DFT calculations for the comparison between triplet nitrene and nitrene radical anion formation. f) DFT calculations for the operating mechanism in the HAT step. g) HAT mechanism for non‐activated cyclohexane. All calculations have been carried out at the ωB97xD/Def2TZVPP//ωB97xD/Def2SVP level of theory. Energies in kcal mol^−1^, bond lengths, labeled in black, in Åmstrong and spin densities are labeled in blue. TXO, Thioxanthone.

With the mechanistic insight gathered based on experimental results, DFT calculations were conducted using the Gaussian 16 software package at the SMD(CHCl_3_)‐ωB97xD/Def2TZVPP//ωB97xD/Def2SVP level of theory (see Supporting Information for full computational details). Based on the insights from the reaction with radical traps, the energy transfer mechanism leading to the formation of triplet nitrene intermediates should be expected. However, Koenigs have previously reported the potential generation of nitrene radical anions via a single electron transfer process between photocatalysts and iminoiodinanes.^[^ ^61^, ^62^, ^63^ ^]^ To shed light on the operative pathway in our transformation, both mechanisms were evaluated in parallel using iminoiodinane **2b** and cyclohexane as model substrates (Figure 3e). For the SET pathway, single electron transfer from the triplet‐state photocatalyst to **2b** occurs by overcoming a barrier of 9.6 kcal mol^−1^, which upon iodobenzene loss, leads to the formation of the corresponding nitrene radical anion. Alternatively, **2b** can undergo energy transfer from the excited photocatalyst. Following the asymmetric approach for energy transfer calculations recently reported by Maseras, ^[^ ^76^ ^]^ we found a remarkably low barrier of 2.7 kcal mol^−1^ for formation of the triplet nitrene after iodobenzene release. The obtained results clearly indicate the preferential formation of the triplet nitrene upon reacting iminoiodinanes and thioxanthone under photocatalytic conditions. This study represents one clear example where the energy barriers required for SET and EnT mechanism are calculated and compared between them, which is usually challenging in DFT mechanistic studies. We thus proceeded to explore the next step of the transformation, the HAT with cyclohexane in both potential mechanisms (Figure 3g). In this regard, HAT from the nitrene radical anion proceeds with a barrier of 20.8 kcal mol^−1^, generating the cyclohexyl radical and anionic sulfamate which subsequently lead to the formation of the aminated product. In contrast, HAT from the energy transfer pathway involves a significantly lower barrier (12.8 kcal mol^−1^) than the SET one. These computational findings shed light on the considerably higher ability for nitrene radicals to undergo HAT processes over the nitrene radical anion. Finally, both radicals can rapidly react in a barrierless process to form the final product **3b’**.

To complete this study, we then explored the potential of the activated photocatalyst to undergo direct HAT with cyclohexane, as TXO has been previously reported to act as HAT reagent.^[^ ^77^ ^]^ The obtained free energy barrier for this process (16.2 kcal mol^−1^) is lower than the one corresponding to the nitrene radical anion but considerably higher than the one for the nitrene HAT, clearly proved the intermediacy of the triplet nitrene in our operating reaction pathway.

To provide an additional experimental result validating DFT calculations, the reactivity of cyclohexane‐d_12_ was explored. When submitting deuterated cyclohexane to the standard reaction protocol, the desired amination took place, albeit the corresponding *d*_11_‐cyclohexylamine derivative **3b’‐*d*_11_** was isolated in a significantly lower yield of 32% (Figure 3d). The crude analysis of this reaction also indicated deuterium transfer from C to N‐atom. Two parallel reactions using substrates **1b** and **1b‐*d*_12_** were performed under identical conditions, and the measured kinetic isotope effect (KIE) was 3.03 (comparison of the initial reaction rates, for the details see Figure S11), indicating that C─H cleavage step can thus be considered as the rate‐determining step, as confirmed by the DFT calculations. Accordingly, it can be concluded that, based on experimental evidence and theoretical investigations, our transformation proceeds via an unprecedented cooperative energy transfer and HAT mechanism (Figure 3f).

To demonstrate the synthetic utility of this transformation, the reaction was conducted on larger scale (2.4 mmol) and the desired product could be isolated in comparable 52% yield. Subsequently, removal of the sulfamate group was investigated (Scheme 2a). Standard protocols using either pyridine‐mediated hydrolysis or Zn‐promoted deprotection performed well. Using this expedient synthetic approach, memantine hydrochloride **7**, an Alzheimer's drug, could thus be accessed in only two steps.

**Scheme 2:**
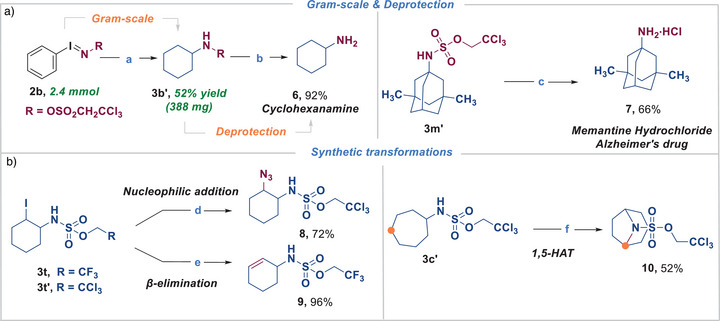
Synthetic applications of the amination protocol; a) Gram‐scale and Deprotection. ^a)^ **2b** (2.4 mmol), **1b** Cyclohexane (10.0 equiv.), Iodosobenzene (2.5 equiv.), and TXO (5.0 mol%) in dry CHCl_3_ (0.1 M), irradiation with LEDs (*λ*_max_ = 370 nm, 2 x 40 W at 100% intensity) under nitrogen atmosphere at room temperature for 16 h; ^b)^5.0 equiv. of Zn powder, CH_3_OH:AcOH 1:1, 40 °C, 16 h; ^c)^20.0 equiv. of Pyridine, 100 °C, 24 h. b) Synthetic transformations. ^d)^5.0 equiv. of NaN_3_, DMF (0.1 M), rt, 16 h; ^e)^CH_3_CN, 100 °C, 16 h; ^f)^4.0 equiv. of 1,3‐diiodo‐5,5‐dimethylhydantoin, CH_3_CN, 80 °C, 24 h. d.r. > 20:1.

Post‐modifications of the aminated compounds could also be conducted efficiently (Scheme 2b). The nucleophilic substitution of **3t′** with sodium azide to furnish 2,2,2‐trichloroethyl (2‐azidocyclohexyl)sulfamate **8** in an excellent yield of 72%. Alternatively, cyclohex‐2‐en‐1‐ylsulfamate **9** was efficiently accessed via thermal *β*‐elimination of intermediate **3t**. Moreover, additional HAT‐type C─H functionalization of substrate **3c’** delivered 8‐azabicyclo[3.2.1]octane‐8‐sulfonate **10** in 52% yield.

In conclusion, new reactivity of nitrene radicals in direct amination of hydrocarbons has been discovered. This photocatalytic reaction occurs smoothly in the presence of TXO photosensitizer and is particularly suitable to convert simple hydrocarbons into valuable amines. Detailed mechanistic studies demonstrate the unusual activation of iminoiodinanes via cooperative Energy Transfer / HAT mechanism, the key toward the desired reactivity.

## Supporting Information

Experimental details and characteristic of compounds are indicated in Supporting Information.

## Conflict of Interests

The authors declare no conflict of interest.

## Supporting information



Supporting Information

Supporting Information

## Data Availability

The data that support the findings of this study are available from the corresponding author upon reasonable request.
