# Supplementary material for: Harnessing Cooperative Energy Transfer and Hydrogen Atom Transfer for Direct Nitrogenations of Non‐Activated Alkanes
Source: Angew Chem Int Ed Engl. 2025 Nov 28;65(3):e18795. doi: 10.1002/anie.202518795 (PMC12811655; doi:10.1002/anie.202518795)
Supplement: Supplementary file 1 — Supporting Information [file ANIE-65-e18795-s002.docx]

*Supporting Information*

Harnessing Cooperative Energy Transfer and Hydrogen Atom Transfer for Direct Nitrogenations of non-activated Alkanes.

Sai Teja Kolla,^a^ Àlex Díaz-Jiménez, ^a^ Sven Trienes,^b^ Ignacio Funes-Ardoiz,^c*^ Joanna Wencel-Delord^a,d*^

1. Institute of Organic Chemistry, JMU Würzburg Am Hubland, Würzburg, Germany
2. Institut für Organische und Biomolekulare Chemie and Wöhler Research Institute for Sustainable Chemistry (WISCh), Georg-August-Universität, Tammannstraße 2, Göttingen 37077, Germany
3. Departamento de química, Instituto de Investigación Química de la Universidad de La Rioja (IQUR), Universidad de La Rioja, Madre de Dios 53, 26004, Logroño, Spain
4. Prof. J. Wencel-Delord, Laboratoire d’Innovation Moléculaire et d’Applications (CNRS, UMR 7042), Université de Strasbourg, 25 rue de Becquerel, Strasbourg, 67037 Strasbourg, France

Contents

[1. General Considerations 3](#_Toc211610573)

[2. Experimental procedures and characterization data 5](#_Toc211610574)

[2.1 Reagent synthesis 5](#_Toc211610575)

[2.1.1 General Procedure for the preparation of sulfamates (GP-A) 5](#_Toc211610576)

[2.1.2 Procedure for the preparation of alcohol derivatives: 7](#_Toc211610577)

[2.1.3 Sulfamates for Intra-molecular amination: 8](#_Toc211610578)

[2.1.4 Synthesis of iodosylbenzene: 8](#_Toc211610579)

[2.1.5 Synthesis of Iminoiodinane (GP-B): 9](#_Toc211610580)

[3. Optimization of the reaction conditions 10](#_Toc211610581)

[4. Substrate Scope 15](#_Toc211610582)

[4.1 General Procedure B (GP-C) 15](#_Toc211610583)

[4.2 Limitations (Unsuccessful substrates): 31](#_Toc211610584)

[5. Gram-Scale synthesis: 32](#_Toc211610585)

[6. Investigations of reaction conditions on Intra-molecular amination: 33](#_Toc211610586)

[6.1 General Procedure D (GP-D) 35](#_Toc211610587)

[6.2 Substrate Scope: 35](#_Toc211610588)

[7. Synthetic Transformations: 40](#_Toc211610589)

[8. X-ray analysis: 44](#_Toc211610590)

[9. Mechanistic investigations: 45](#_Toc211610591)

[9.1 UV/Visible absorption spectroscopy: 45](#_Toc211610592)

[9.2 Stern-Volmer luminescence quenching analysis: 46](#_Toc211610593)

[9.4 Table S11 Comparison of various triplet photocatalysts. 49](#_Toc211610594)

[9.5 Radical trapping experiments 50](#_Toc211610595)

[9.6 Radical quenching experiments:^[17]^ 51](#_Toc211610596)

[9.7 Light on/off experiments: 51](#_Toc211610597)

[9.8 Study of the reaction with deuterated substrate: 52](#_Toc211610598)

[9.9 Kinetic Isotopic effects: KIE is determined from two parallel reactions: 53](#_Toc211610599)

[9.9.1 Radical clock experiment: 54](#_Toc211610600)

[10. Computational studies 55](#_Toc211610601)

[10.1. Computational details: 55](#_Toc211610602)

[10.2 Free energy profile for the intermolecular amination of 1a’: 57](#_Toc211610603)

[10.3. Hydrogen atom transfer step for toluene: 58](#_Toc211610604)

[10.4. Free energy profile for the intramolecular amination of 4a’: 58](#_Toc211610605)

[10.5. Regioselectivity studies 59](#_Toc211610606)

[11. XYZ Coordinates 60](#_Toc211610607)

[12. References: 90](#_Toc211610608)

[13. Spectral data: 93](#_Toc211610609)

# 1. General Considerations

Unless otherwise stated, all reactions were carried out under atmosphere of nitrogen in oven-dried or flame-dried glassware. Prior to the reaction set-up, glassware was evacuated and backfilled with argon three times. The solvents chloroform, dichloromethane (DCM), methanol (MeOH), toluene, tetrahydrofuran (THF) and N,N-dimethylformamide (DMF) applied in synthesis were purified by solvent purification system (SPS) over standard drying materials with positive argon flow and stored under argon. Ethyl acetate (EtOAc) and acetonitrile (MeCN) were stored over molecular sieves under nitrogen.


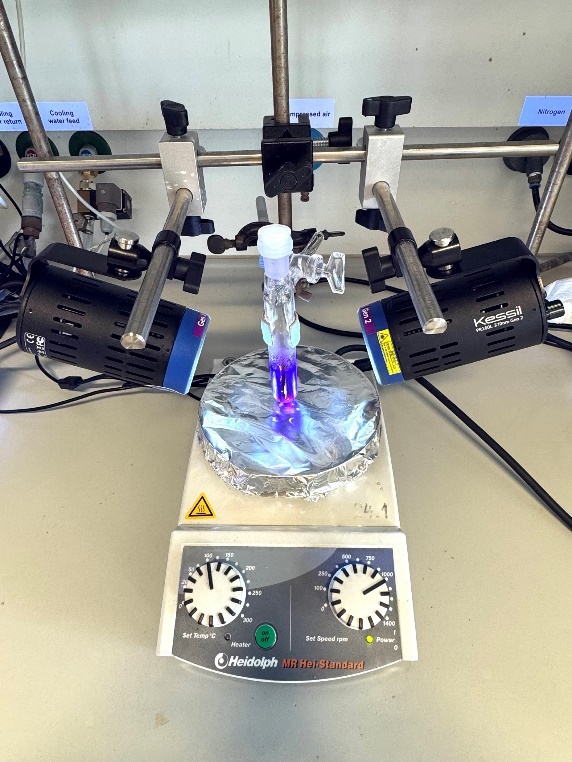

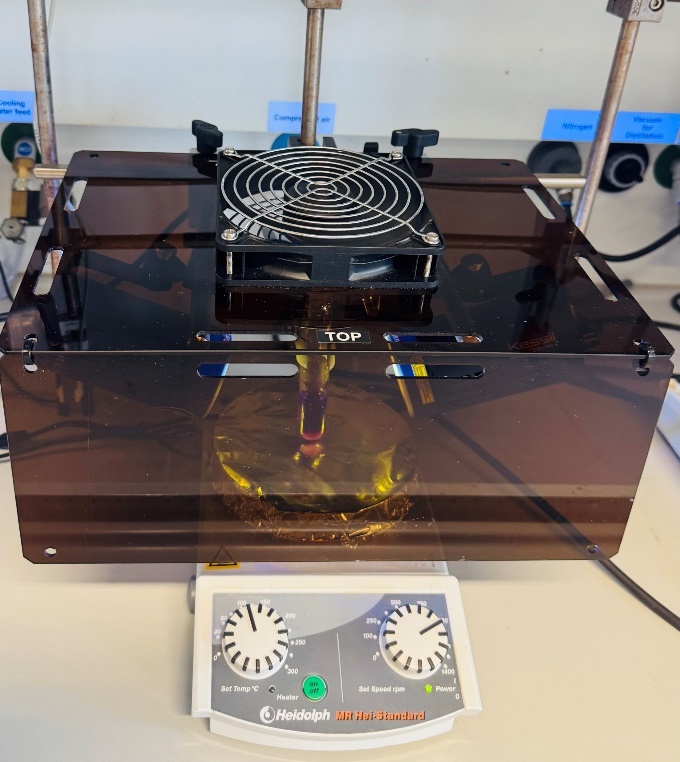
***Photochemical set-up and light sources*** Photochemical reactions were performed using two Kessil®PR16L-370-G2 LEDs (40 W, λ_max_ = 370 nm), one Kessil®PR16L-390 LEDs (40 W, λ_max_ = 390 nm), one EvoluChem™ HCK1012-010 LEDs (18 W, λ_max_ = 405 nm), EvoluChem™ HCK1012-002 LEDs (18 W, λ_max_ = 450 nm) and one Kessil®PR16L-467 LEDs (40 W, λ_max_ = 467 nm). To shield irradiation, the setup was covered with a PR160 Rig w/ Fan Kit with a powerful fan to blow air from the top efficiently. The reaction temperature was determined to be between 35 °C and 38 °C using this setup.

**Figure S1**: Experimental set-up for photochemical reactions.

**Column chromatography and solvents** Analytical thin layer chromatography (TLC) was performed using silica gel 60 F254 aluminum plates by Merck. TLC plates were visualized by exposure to short wave ultraviolet light (254 nm or 365 nm) and/or were dipped into a solution of KMnO_4_ (3.0 g) and K_2_CO_3_ (10.0 g) in H_2_O (300 mL). Column chromatography was carried out using silica gel (60 mesh) by Macherey-Nagel GmbH & Co. KG eluting with the below mentioned solvent system under positive pressurized air flow. Petroleum ether, chloroform, DCM and ethyl acetate for column chromatography or recrystallization were purchased of technical grade and further purified *via* distillation. Unless otherwise mentioned, dry solvents purchased from Thermo Fisher (HPLC grade) and used to perform the preparation of starting materials and catalytic reactions. The following solvents were purified using a custom solvent purification system (SPS) with activated alumina columns (built by the Dept. of Chemistry and Pharmacy, University of Wuerzburg”) and collected under positive argon pressure: THF, DMF, toluene, MeOH and DCM.

***NMR spectroscopy*** was recorded on Bruker Avance III HD 400 MHz and 600 MHz spectrometers. Chemical shift data for protons are reported in parts per million (ppm, δ scale) downfield from tetramethylsilane and referenced internally to the residual proton in the solvent (CDCl_3_: δ 7.26 ppm, DMSO-*d_6_*: δ 2.50). Chemical shift data for carbons are reported in parts per million (ppm, δ scale) downfield from tetramethylsilane, and referenced internally to the carbon resonance in the solvent (CDCl_3_: δ 77.20, DMSO-*d_6_*: δ 39.52). NMR data analysis is presented as following, s: singlet, d: doublet, t: triplet, m: multiplet, br: broad signal, coupling constant in Hertz (Hz), and integration. All the NMRs were processed using Mestrenova 14 applying standard phase and baseline corrections. Coupling constants (J) are quoted in Hz. Crude yields were determined by ^1^H using mesitylene as internal standard.

**High resolution mass spectra** were measured on a Bruker Daltonics micrOTOF-QIII focus instrument for high resolution ESI. As a note, we like to mention that the often-observed peak at 685.4 is attributable to Irganox, an additive of plastics that contaminates the samples upon dissolution from plastic vials for mass spectrometry.

**Single crystal X-ray analysis** was carried out on Bruker D8 Quest Kappa diffractometers with a PhotonII CMOS detector and multi-layered mirror monochromated CuKα radiation.

**Chemicals:** The alkanes were mostly all commercially available and purchased from TCI, Thermo fisher, BLD pharma, Sigma Aldrich and deuterated cyclohexane was purchased from ABCR chemicals and used as received. Photocatalysts such as thioxanthone and it’s derivatives, (Ir[dF(CF_3_)ppy]_2_(dtbbpy))PF_6_, (Ir-F), [Mes_2_Acr]ClO_4_, 4-CzIPN, and fac-Ir(ppy)_3_ were purchased from Sigma Aldrich or BLD pharma and used as received.

**Availability of starting materials:** Commercially available alkanes were purchased from Thermoscientific – Acros, BLD pharma, Sigma Aldrich, Apollo Scientific, Fluorochem, TCI and ABCR.

**Remarks**: Careful chromatographic purification was necessary due to the formation of closely related trace amounts of byproducts.

# 2. Experimental procedures and characterization data

## 2.1 Reagent synthesis

### 2.1.1 General Procedure for the preparation of sulfamates (GP-A)

A flask under argon was charged with chlorosulfonyl isocyanate (6.6 mmol, 2.00 eq.). The flask was cooled to 0 °C, and formic acid (HCO₂H, 6.6 mmol, 2.00 eq.) was added slowly with vigorous stirring, during which intense gas evolution was observed. The mixture solidified within 5 minutes. Anhydrous MeCN (2.5 mL) was added, and the resulting solution was stirred for 30 minutes before the ice bath was removed. After 3 h at room temperature, the mixture was cooled again to 0 °C, then a solution of alcohol (3.3 mmol, 1.00 eq.) in anhydrous DMA (5.0 mL) was added slowly over 30 minutes using syringe pump. The mixture was stirred at 0 °C for 30 minutes, and then the ice bath was removed. After stirring for overnight at room temperature, water (10.0 mL) was added. The solution was extracted three times with ethyl acetate, and the combined organic layers were washed with water, dried over Na₂SO₄, filtered, and concentrated. The residue was purified by column chromatography on silica gel (petroleum ether/EtOAc 7:3) to afford the expected product.

Sulfamates were synthesized according to the corresponding literature procedures. ^[1]^

**2,2,2-trifluoroethyl sulfamate (1) :** ([***See spectra***](#one))

Was synthesized from chlorosulfonyl isocyanate (0.57 mL, 6.6 mmol, 2.00 eq.) and 2,2,2-trifluoroethanol (0.24 mL, 3.3 mmol, 1.00 eq.) following [**GP-A**](#GPA). Purification by column chromatography on silica gel (petroleum ether/EtOAc 7:3) afforded the desired product (0.45 g, 2.53 mmol, 77%) as a white solid.

**TLC**: R*_f_* = 0.30 (80:20 Petroleum ether: EtOAc).

**^1^H NMR** (400 MHz, CDCl_3_) δ 4.99 (s, 2H), 4.49 (q, *J* = 8.0 Hz, 2H) ppm.

**^13^C NMR** (101 MHz, CDCl_3_) δ 123.5, 120.8, 118.0, 65.8 (q, *J* = 38.1 Hz) ppm.

**^19^F NMR** (376 MHz, CDCl_3_) δ -73.79 (t, *J* = 8.0 Hz) ppm.

**HRMS** (ESI) *m/z* calculated for C_2_H_3_F_3_NO_3_S [M-H]^-^ 177.9791, found 177.9783.

**2,2,2-trichloroethyl sulfamate (2) :** ([***See spectra***](#two))

Was synthesized from chlorosulfonyl isocyanate (0.57 mL, 6.6 mmol, 2.00 eq.) and 2,2,2-trifluoroethanol (0.32 mL, 3.3 mmol, 1.00 eq.) following [**GP-A**](#GPA). Purification by column chromatography on silica gel (petroleum ether/EtOAc 7:3) afforded the desired product (0.57 g, 2.52 mmol, 75%) as a white solid.

**TLC**: R*_f_* = 0.30 (80:20 Petroleum ether: EtOAc).

**^1^H NMR** (400 MHz, CDCl_3_) δ 5.09 (s, 2H), 4.70 (s, 2H) ppm.

**^13^C NMR** (101 MHz, CDCl_3_) δ 93.3, 78.9 ppm.

**HRMS** (ESI) *m/z* calculated for C_2_H_3_Cl_3_NO_3_S [M-H]^-^ 225.8904, found 225.8903.

### 2.1.2 Procedure for the preparation of alcohol derivatives:

A flame-dried flask under argon was charged with magnesium turnings (0.30 g, 12.3 mmol, 1.23 eq.) and anhydrous diethyl ether (10.0 mL). A solution of *n*-propyl bromide (1.54 g, 12.5 mmol, 1.25 eq.) in diethyl ether was added dropwise with stirring. The reaction mixture was stirred at room temperature for 2 h to complete formation of the Grignard reagent. In a separate flask, 3-methylbutanal (0.86 g, 10.0 mmol, 1.00 eq.) was dissolved in anhydrous diethyl ether (100 mL). This solution was added dropwise at 0 °C to the n-propyl-magnesium bromide (Grignard reagent) with vigorous stirring. After complete addition, the ice bath was removed, and the mixture was stirred at room temperature for 2 h. The reaction was quenched with 1.0 M HCl then the aqueous phase was extracted 3 times with Et₂O (3 × 50 mL), and the combined organic layers were washed with water, dried over Na₂SO₄, filtered, and concentrated under reduced pressure to afford the crude alcohol product used directly without purification for the next step.

All above alcohols were synthesized according to previously reported literature procedures.^[2]^

### 2.1.3 Sulfamates for Intra-molecular amination:

The corresponding alcohols were subjected to prepare various sulfamates for intramolecular cyclization using [**GP-A**](#GPA)

### 2.1.4 Synthesis of iodosylbenzene:

A round bottom flask was charged with bis(acetoxy)iodobenzene (PIDA) (10.0 g, 31.04 mmol) and aqueous NaOH (3 M, 220 mL) solution. The reaction mixture stirred vigorously for 16 hours at room temperature. Then, the precipitate formed was filtered off and washed with water until pH of water became neutral. Then the solid was washed (2 × 50 mL) with chloroform to remove impurities of PIDA. The obtained solid was dried at high vacuum without heating to yield iodosylbenzene [(PhIO)_n_] (6.68 g, 30.4 mmol, 98% yield) as yellow-tinted white solid which was directly used in the next step. *We found that heating the material resulted in a white solid which didn’t react with methanol. We attribute this difference to disproportionation resulting in PhIO_2_ and PhI. Heating of this compound is not recommended as this may explode.*

***Note****: The product was stored at −20 °C in an aluminium foil wrapped scintillation vial, purged with argon after usage and returned to storage as quickly as possible.*

Synthesized according to the corresponding literature procedures.^[3]^

### 2.1.5 Synthesis of Iminoiodinane (GP-B):

A round bottom flask was charged with sulfamate (5.0 mmol, 1.0 equiv.) and iodosylbenzene (1.21 g, 1.1 equiv., 5.5 mmol) in DCM (20 mL). The reaction mixture was stirred vigorously for 3 hours at room temperature. Then, the precipitate formed was filtered off and washed with DCM (2 × 20 mL). The obtained solid was dried at high vacuum for overnight to afford iminoiodinane as white solid.

**2,2,2-trifluoroethyl (phenyl-*λ*^3^-iodaneylidene) sulfamate** (**2a**): ([***See spectra***](#a2))

Was synthesized from 2,2,2-trifluoroethyl sulfamate (0.90 g, 5.0 mmol, 1.00 eq.) and iodosylbenzene (1.21 g, 5.5 mmol, 1.10 eq.) following [**GP-B**](#GPB) afforded 2,2,2-trifluoroethyl (phenyl-*λ*^3^-iodaneylidene) sulfamate (1.79 g, 4.68 mmol, 94%) as a white solid.

**^1^H NMR** (400 MHz, CD_3_OD) δ 8.09 – 7.97 (m, 2H), 7.74 – 7.50 (m, 3H), 4.51 (q, *J* = 8.5 Hz, 2H) ppm.

**^13^C NMR** (101 MHz, CD_3_OD) δ 133.3, 132.2, 132.0, 127.9 (q, *J* = 277.0 Hz), 122.6, 65.5 (q, *J* = 37.0 Hz) ppm.

**^19^F NMR** (376 MHz, CD_3_OD) δ -75.80 (t, *J* = 8.5 Hz) ppm.

**HRMS** (ESI) *m/z* calculated for C_8_H_7_F_3_INNaO_3_S [M+Na]^+^ 403.9035, found 403.9044.

***Note****: The product was stored at −20 °C in an aluminium foil wrapped scintillation vial, purged with argon after usage and returned to storage as quickly as possible.*

***Additional note****: The NMR is not clear due to decomposition of the compound during the analysis.*

**2,2,2-trichloroethyl (phenyl-*λ*^3^-iodaneylidene) sulfamate** (**2b**): ([***See spectra***](#b2))

Was synthesized from 2,2,2-trichloroethyl sulfamate (1.14 g, 5.0 mmol, 1.00 eq.) and iodosylbenzene (1.21 g, 5.5 mmol, 1.10 eq.) following [**GP-B**](#GPB) afforded 2,2,2-trichloroethyl (phenyl-*λ*^3^-iodaneylidene) sulfamate (1.96 g, 4.55 mmol, 91%) as a white solid.

**^1^H NMR** (400 MHz, CD_3_OD) δ 8.04 – 8.01 (m, 2H), 7.57 – 7.55 (m, 1H), 4.67 (s, 2H) ppm.

**^13^C NMR** (101 MHz, CD_3_OD) δ 133.3, 132.2, 132.0, 95.29, 79.0, 78.9 ppm.

**HRMS** (ESI) calculated for C_8_H_7_Cl_3_INNaO_3_S [M+Na]^+^ 451.8149, found 451.8157.

***Note****: The product was stored at −20 °C in an aluminium foil wrapped scintillation vial, purged with argon after usage and returned to storage as quickly as possible.*

***Additional note****: The NMR is not clear due to decomposition of the compound during the analysis.*

Synthesized according to the corresponding literature procedures.^[4]^

All reactions were carried out according to General Procedure A (**GP-B**) on a 0.5 mmol scale using iodosobenzene (0.55 mmol, 1.1 equiv.) in DCM (5 mL) at room temperature for 3 h.

# 3. Optimization of the reaction conditions

**Procedure**: All reactions were performed on 0.2 mmol scale. To a 10 mL Schlenk tube equipped with a Teflon coated magnetic stir bar, 2,2,2-trifluoroethyl (phenyl-*λ*^3^-iodaneylidene) sulfamate **2a** (54 mg, 0.14 mmol, 1.0 equiv.), cyclohexane **1b** (118.0 mg (153 µL), 1.4 mmol, 10.0 equiv.), additive (X equiv.) and photocatalyst (X equiv.) were added. The Schlenk tube was evacuated and backfilled with nitrogen three times. Subsequently, solvent (1.2 mL) was added against the positive flow of nitrogen. The reaction mixture was then stirred under irradiation with LEDs (40 W,

λ_max_ = 370 nm) at room temperature. Later, the solvent was removed *in* *vacuo*. Mesitylene (5.5 µL, 0.4 mmol) was added as internal standard, and the yield was determined by crude ^1^H NMR analysis.

**Table S1. Solvent Optimization**.^[a]^

| Entry | PC | Solvent | Yield of 3b (%)^[b]^ |
| --- | --- | --- | --- |
| 1 | Thioxanthone | DMF | n.d |
| 2 | Thioxanthone | THF | n.d |
| 3 | Thioxanthone | Acetone | n.d |
| 4 | Thioxanthone | MeOH | n.d |
| 5 | Thioxanthone | CCl_4_ | n.d |
| 6 | Thioxanthone | Toluene | n.d |
| 7 | Thioxanthone | MTBE | 17 |
| 8 | Thioxanthone | EtOAC | 19 |
| 9 | Thioxanthone | DCM | 37 |
| 10 | Thioxanthone | CHCl_3_ | 46 |

^[a]^**Reaction conditions**: 2,2,2-trifluoroethyl (phenyl-λ^3^-iodaneylidene) sulfamate (0.14 mmol), Cyclohexane (10.0 equiv.), and PC (5.0 mol%) in dry solvent (0.1 M), irradiation with LEDs (λ_max_ = 370 nm) under nitrogen atmosphere at room temperature for 16 h. ^[b]^Determined by crude NMR using mesitylene as internal standard. DMF, Dimethylformamide. THF, Tetrahydrofuran. MeOH, Methanol. CCl_4_, Carbon tetrachloride. MTBE, Methyl *tert*-butyl ether. EtOAc, Ethyl acetate. DCM, Dichloromethane and CHCl_3_, Chloroform.

**Table S2. LEDs optimization.**^[a]^

| Entry | LEDs | Intensity of LED | Yield of 3b (%)^[b]^ |
| --- | --- | --- | --- |
| 1 | 370 nm (40W) | 75% | 29 |
| 2 | 370 nm (40W) | 100% | 43 |
| 3 | 390 nm (40W) | 100% | 22 |
| 4 | 405 nm (18W) | 100% | 18 |
| 5 | 450 nm (18W) | 100% | 10 |
| 6 | 465 nm (40W) | 100% | <10 |

^[a^**]Reaction conditions**: 2,2,2-trifluoroethyl (phenyl-λ^3^-iodaneylidene) sulfamate (0.14 mmol), Cyclohexane (10.0 equiv.), and PC (5.0 mol%) in dry CHCl_3_ (0.1 M), irradiation with LEDs under nitrogen atmosphere at room temperature for 16 h. ^[b]^Determined by crude NMR using mesitylene as internal standard.

**Table S3. Additive optimization.**^[a]^

| Entry | Additive (equiv.) | Yield of 3b (%)^[b]^ |
| --- | --- | --- |
| 1 | PIDA (1.0) | 40 |
| 2 | PIFA (1.0) | 38 |
| 3 | BF_3_.OEt_2_ (1.0) | 20 |
| 4 | PhIO (1.0) | 48 |
| 5 | PhIO (2.0) | 51 |
| 6 | PhIO (2.5) | 58 |
| 7 | PhIO (3.0) | 53 |

^[a]^**Reaction conditions**: 2,2,2-trifluoroethyl (phenyl-λ^3^-iodaneylidene) sulfamate (0.14 mmol), Cyclohexane (10.0 equiv.), additive (1-3 equiv.) and TXO (5.0 mol%) in dry CHCl_3_ (0.1 M), irradiation with LEDs (λ_max_ = 370 nm) under nitrogen atmosphere at room temperature for 16 h. ^[b]^Determined by crude NMR using mesitylene as internal standard.

**Table S4. Thioxanthone derivatives optimization.**^[a]^

| Entry | PC | Yield of 3b (%)^[b]^ |
| --- | --- | --- |
| 1 | 2-CF_3_TXO | 18 |
| 2 | 2,2’-BrTXO | 21 |
| 3 | 2,2’-OMeTXO | 32 |
| 4 | 2-ITXO | 38 |
| 5 | 4-OMeTXO | 41 |
| 6 | TXO | 58 |

^[a]^**Reaction conditions**: 2,2,2-trifluoroethyl (phenyl-λ^3^-iodaneylidene) sulfamate (0.14 mmol), Cyclohexane (10.0 equiv.), PhIO (2.5 equiv.) and PC (5.0 mol%) in dry CHCl_3_ (0.1 M), irradiation with LEDs (λ_max_ = 370 nm) under nitrogen atmosphere at room temperature for 16 h. ^[b]^Determined by crude NMR using mesitylene as internal standard.

**Table S5. Deviation from standard conditions.**^[a]^

| Entry | Variation from the standard conditions | Yield of 3b (%)^[b]^ |
| --- | --- | --- |
| 1 | None | 58 |
| 2 | TXO (10 mol%) | 38 |
| 3 | EtOAc (0.1M) | 20 |
| 4 | DCM (0.1M) | 48 |
| 5 | 4CzIPN (5 mol%) | ~10 |
| 6 | TBADT (5 mol%) | 40 |
| 7 | 390, 405, 450 nm | 51, 28, 20 |
| 8 | 2,2’-BrTXO, 2,2’-OMeTXO, 2-ITXO, 4-OMeTXO | 21, 32, 38, 41 |
| 9 | without *hv* | 0 |
| 10 | without PC | 0 |
| 11 | only heat, no *hv* (60^o^C) | 0 |

^[a]^**Reaction conditions**: 2,2,2-trifluoroethyl (phenyl-λ^3^-iodaneylidene) sulfamate (0.14 mmol), Cyclohexane (10.0 equiv.), PhIO (2.5 equiv.) and TXO (5.0 mol%) in dry CHCl_3_ (0.1 M), irradiation with LEDs (λ_max_ = 370 nm) under nitrogen atmosphere at room temperature for 16 h. ^[b]^Determined by crude NMR using mesitylene as internal standard.

*As noted from* ***Table S5****, the reaction scheme works well with organic metal-free catalyst thioxanthone, however TXO proves to be the better catalyst. On conducting experiments without visible light, photocatalyst or only heat, no desired product formation was detected.*

**Comparison of the reactivity using 4CzIPN under various reaction conditions**

| S.No | Catalyst | Solvent | Lamp (nm) | Conversion (%) |
| --- | --- | --- | --- | --- |
| 1 | 4-CzIPN | Dry CHCl_3_ | 370 | ~10 | |
| 2 | 4-CzIPN | Dry CH_3_CN | 370 | 12 | |
| 3 | 4-CzIPN | Dry CHCl_3_ | 450 | 23 | |

**Reaction with sulfamate (*in situ*)**:

***Note:*** *When the reaction was performed using sulfamate with in situ generation of (phenyl-λ³-iodaneylidene)sulfamate, a 52% yield was obtained. In contrast, using pre-formed (phenyl-λ³-iodaneylidene)sulfamate gave a higher yield of 58%. Therefore, we opted to prepare the reagent in advance and proceeded with it for scope studies.*

# 4. Substrate Scope

## 4.1 General Procedure B (GP-C)

To an 10 mL Schlenk tube equipped with a Teflon coated magnetic stir bar was added (phenyl-*λ*^3^-iodaneylidene) sulfamate (0.14 mmol), iodosylbenzene (0.35 mmol, 2.5 equiv.) and 9*H*-thioxanthen-9-one (5 mol%) in dry CHCl_3_ (0.1 M). The resulting mixture was degassed *via* ‘freeze-pump-thaw’ procedure (3 times). Subsequently, alkane (5.0 equiv. or 10 equiv.) (if alkane was a solid, it was added prior solvent addition) was added against nitrogen positive flow. The reaction mixture was then stirred under irradiation with LEDs (2 x 40 W, λ_max_ = 370 nm) at room temperature (35 °C to 38 °C) for 16 h. Upon completion of the reaction, the crude product was washed with sodium thiosulfate solution (2 x 10 mL) and the organic layer was dried over MgSO_4_. The solvent was removed by vacuum and the crude product was purified by column chromatography on silica gel; eluent: petroleum ether/ethyl acetate.

**2,2,2-trifluoroethyl cyclopentylsulfamate** (**3a**): ([***See spectra***](#a3))

**TLC**: R*_f_* = 0.25 (90:10 Petroleum ether: EtOAc).

The title compound **3a** was synthesized according to [**GP-C**](#GPC) using 2,2,2-trifluoroethyl *N*-(phenyl-λ²-iodanyl)sulfamate **2a** (54 mg, 0.14 mmol, 1.0 equiv.), cyclopentane **1a** (100 mg (130 µL), 1.4 mmol, 10.0 equiv.), iodosylbenzene (77 mg, 0.35 mmol, 2.5 equiv.) and 9*H*-thioxanthen-9-one (1.5 mg, 0.007 mmol, 5 mol%) in dry CHCl_3_ (1.5 mL, 0.1 M). The product was isolated by silica gel column chromatography using a gradient of petroleum ether/ethyl acetate (10:1) (22 mg, 0.089 mmol, 63%) as a colourless oil.

**^1^H NMR** (400 MHz, CDCl_3_) δ 4.93 – 4.86 (bs, 1H), 4.39 (q, *J* = 8.0 Hz, 2H), 3.82 (h, *J* = 6.9 Hz, 1H), 2.08 – 1.92 (m, 2H), 1.78 – 1.46 (m, 6H) ppm.

**^13^C NMR** (101 MHz, CDCl_3_) δ 122.3 (q, *J* = 277.5 Hz), 65.1 (q, *J* = 37.8 Hz), 56.6, 33.2, 23.2 ppm.

**^19^F NMR** (376 MHz, CDCl_3_) δ -73.79 (t, *J* = 8.0 Hz) ppm.

**HRMS** (ESI) *m/z* calculated for C_7_H_12_F_3_NNaO_3_S [M+Na]^+^ 270.0710, found 270.0711.

**2,2,2-trichloroethyl cyclopentylsulfamate** (**3a’**): ([***See spectra***](#aa3))

**TLC**: R*_f_* = 0.25 (90:10 Petroleum ether: EtOAc).

The title compound **3a’** was synthesized according to [**GP-C**](#GPC) using 2,2,2-trichloroethyl *N*-(phenyl-λ²-iodanyl)sulfamate **2b** (60 mg, 0.14 mmol, 1.0 equiv.), cyclopentane **1a** (100 mg (130 µL), 1.4 mmol, 10.0 equiv.), iodosylbenzene (77 mg, 0.35 mmol, 2.5 equiv.) and 9*H*-thioxanthen-9-one (1.5 mg, 0.007 mmol, 5 mol%) in dry CHCl_3_ (1.5 mL, 0.1 M). The product was isolated by silica gel column chromatography using a gradient of petroleum ether/ethyl acetate (10:1) (23 mg, 0.078 mmol, 56%) as a white solid.

**^1^H NMR** (400 MHz, CDCl_3_) δ 4.64 (bs, 1H), 4.62 (s, 2H), 3.99 – 3.84 (m, 1H), 2.11 – 1.98 (m, 2H), 1.73 – 1.54 (m, 6H) ppm.

**^13^C NMR** (101 MHz, CDCl_3_) δ 93.7, 78.3, 56.7, 33.5, 23.4 ppm.

**HRMS** (ESI) *m/z* calculated for C_7_H_12_Cl_3_NNaO_3_S [M+Na]^+^ 317.9495, found 317.9495.

**2,2,2-trifluoroethyl cyclohexylsulfamate** (**3b**): ([***See spectra***](#b3))

**TLC**: R*_f_* = 0.25 (90:10 Petroleum ether: EtOAc).

The title compound **3b** was synthesized according to [**GP-C**](#GPC) using 2,2,2-trifluoroethyl *N*-(phenyl-λ²-iodanyl)sulfamate **2a** (54 mg, 0.14 mmol, 1.0 equiv.), cyclohexane **1b** (118 mg (153 µL), 1.4 mmol, 10.0 equiv.), iodosylbenzene (77 mg, 0.35 mmol, 2.5 equiv.) and 9*H*-thioxanthen-9-one (1.5 mg, 0.007 mmol, 5 mol%) in dry CHCl_3_ (1.5 mL, 0.1 M). The product was isolated by silica gel column chromatography using a gradient of petroleum ether/ethyl acetate (10:1) (21 mg, 0.080 mmol, 58%) as a white solid.

**^1^H NMR** (400 MHz, CDCl_3_) δ 4.59 (d, *J* = 7.8 Hz, 1H), 4.39 (q, *J* = 8.0 Hz, 2H), 3.37 (dddd, *J* = 14.2, 10.2, 7.7, 3.9 Hz, 1H), 2.06 – 1.99 (m, 2H), 1.74 (dt, *J* = 13.0, 3.9 Hz, 2H), 1.63 – 1.57 (m, 1H), 1.40 – 1.16 (m, 5H) ppm.

**^13^C NMR** (101 MHz, CDCl_3_) δ 122.3 (q, *J* = 277.5 Hz), 65.1 (q, *J* = 37.7 Hz), 54.4, 33.6, 25.2, 24.8 ppm.

**^19^F NMR** (376 MHz, CDCl_3_) δ -73.79 (t, *J* = 8.0 Hz) ppm.

**HRMS** (ESI) *m/z* calculated for C_8_H_14_F_3_NNaO_3_S [M+Na]^+^ 283.9975, found 283.9981.

**2,2,2-trichloroethyl cyclohexylsulfamate** (**3b’**): ([***See spectra***](#bb3))

**TLC**: R*_f_* = 0.25 (90:10 Petroleum ether: EtOAc).

The title compound **3b’** was synthesized according to [**GP-C**](#GPC) using 2,2,2-trichloroethyl *N*-(phenyl-λ²-iodanyl)sulfamate **2b** (60 mg, 0.14 mmol, 1.0 equiv.), cyclohexane **1b** (118 mg (153 µL), 1.4 mmol, 10.0 equiv.), iodosylbenzene (77 mg, 0.35 mmol, 2.5 equiv.) and 9*H*-thioxanthen-9-one (1.5 mg, 0.007 mmol, 5 mol%) in dry CHCl_3_ (1.5 mL, 0.1 M). The product was isolated by silica gel column chromatography using a gradient of petroleum ether/ethyl acetate (10:1) (24 mg, 0.077 mmol, 55%) as a white solid.

**^1^H NMR** (400 MHz, CDCl_3_) δ 4.70 (d, *J* = 7.9 Hz, 1H), 4.61 (s, 2H), 3.49 – 3.37 (m, 1H), 2.11 – 2.02 (m, 2H), 1.78 – 1.70 (m, 2H), 1.63 – 1.57 (m, 1H), 1.37 – 1.24 (m, 5H) ppm.

**^13^C NMR** (101 MHz, CDCl_3_) δ 93.7, 78.3, 54.3, 33.7, 25.2, 24.8 ppm.

**HRMS** (ESI) *m/z* calculated for C_8_H_14_Cl_3_NNaO_3_S [M+Na]^+^ 331.9652, found 331.9653.

Spectral data were consistent with the literature.^[5]^

**2,2,2-trifluoroethyl cycloheptylsulfamate** (**3c**): ([***See spectra***](#c3))

**TLC**: R*_f_* = 0.25 (90:10 Petroleum ether: EtOAc).

The title compound **3c** was synthesized according to [**GP-C**](#GPC) using 2,2,2-trifluoroethyl *N*-(phenyl-λ²-iodanyl)sulfamate **2a** (54 mg, 0.14 mmol, 1.0 equiv.), cycloheptane **1c** (138 mg (170 µL), 1.4 mmol, 10.0 equiv.), iodosylbenzene (77 mg, 0.35 mmol, 2.5 equiv.) and 9*H*-thioxanthen-9-one (1.5 mg, 0.007 mmol, 5 mol%) in dry CHCl_3_ (1.5 mL, 0.1 M). The product was isolated by silica gel column chromatography using a gradient of petroleum ether/ethyl acetate (10:1) (21 mg, 0.076, 55%) as a white solid.

**^1^H NMR** (400 MHz, CDCl_3_) δ 4.74 (d, *J* = 7.8 Hz, 1H), 4.38 (q, *J* = 8.0 Hz, 2H), 3.58 (m, *J* = 8.7, 7.8, 4.4 Hz, 1H), 2.06 – 1.98 (m, 2H), 1.65 – 1.45 (m, 10H) ppm.

**^13^C NMR** (101 MHz, CDCl_3_) δ 122.3 (q, *J* = 277.5 Hz), 65.1 (q, *J* = 37.7 Hz), 56.7, 35.6, 28.0, 23.6 ppm.

**^19^F NMR** (376 MHz, CDCl_3_) δ -73.84 (t, *J* = 8.0 Hz) ppm.

**HRMS** (ESI) *m/z* calculated for C_9_H_16_F_3_NNaO_3_S [M+Na]^+^ 298.0695, found 298.0698.

**2,2,2-trichloroethyl cycloheptylsulfamate** (**3c’**): ([***See spectra***](#cc3))

**TLC**: R*_f_* = 0.25 (90:10 Petroleum ether: EtOAc).

The title compound **3c’** was synthesized according to [**GP-C**](#GPC) using 2,2,2-trichloroethyl *N*-(phenyl-λ²-iodanyl)sulfamate **2b** (60 mg, 0.14 mmol, 1.0 equiv.), cycloheptane **1c** (138 mg (170 µL), 1.4 mmol, 10.0 equiv.), iodosylbenzene (77 mg, 0.35 mmol, 2.5 equiv.) and 9*H*-thioxanthen-9-one (1.5 mg, 0.007 mmol, 5 mol%) in dry CHCl_3_ (1.5 mL, 0.1 M). The product was isolated by silica gel column chromatography using a gradient of petroleum ether/ethyl acetate (10:1) (24 mg, 0.074 mmol, 53%) as a white solid.

**^1^H NMR** (400 MHz, CDCl_3_) δ 4.72 (d, *J* = 8.9 Hz, 1H), 4.61 (s, 2H), 3.64 (m, *J* = 8.5, 4.4 Hz, 1H), 2.11 – 2.03 (m, 2H), 1.65 – 1.46 (m, 10H) ppm.

**^13^C NMR** (101 MHz, CDCl_3_) δ 93.7, 78.3, 56.6, 35.7, 28.0, 23.7 ppm.

**HRMS** (ESI) *m/z* calculated for C_9_H_16_Cl_3_NNaO_3_S [M+Na]^+^ 345.9808, found 345.9793.

**2,2,2-trifluoroethyl cyclooctylsulfamate** (**3d**): ([***See spectra***](#d3))

**TLC**: R*_f_* = 0.25 (90:10 Petroleum ether: EtOAc).

The title compound **3d** was synthesized according to [**GP-C**](#GPC) using 2,2,2-trifluoroethyl *N*-(phenyl-λ²-iodanyl)sulfamate **2a** (54 mg, 0.14 mmol, 1.0 equiv.), cyclooctane **1d** (157 mg (190 µL), 1.4 mmol, 10.0 equiv.), iodosylbenzene (77 mg, 0.35 mmol, 2.5 equiv.) and 9*H*-thioxanthen-9-one (1.5 mg, 0.007 mmol, 5 mol%) in dry CHCl_3_ (1.5 mL, 0.1 M). The product was isolated by silica gel column chromatography using a gradient of petroleum ether/ethyl acetate (10:1) (25 mg, 0.086, 62%) as a colourless oil.

**^1^H NMR** (400 MHz, CDCl_3_) δ 4.47 (d, *J* = 7.9 Hz, 1H), 4.33 (q, *J* = 8.0 Hz, 2H), 3.62 – 3.51 (m, 1H), 1.93 – 1.84 (m, 2H), 1.64 – 1.55 (m, 4H), 1.48 (m, 8H) ppm.

**^13^C NMR** (101 MHz, CDCl_3_) δ 122.3 (q, *J* = 277.6 Hz), 65.1 (q, *J* = 37.7 Hz), 55.8, 32.4, 27.3, 25.3, 23.2 ppm.

**^19^F NMR** (376 MHz, CDCl_3_) δ -73.80 (t, *J* = 8.0 Hz) ppm.

**HRMS** (ESI) *m/z* calculated for C_10_H_18_F_3_NNaO_3_S [M+Na]^+^ 311.9961, found 311.9960.

**2,2,2-trichloroethyl cyclooctylsulfamate** (**3d’**): ([***See spectra***](#dd3))

**TLC**: R*_f_* = 0.25 (90:10 Petroleum ether: EtOAc).

The title compound **3d’** was synthesized according to [**GP-C**](#GPC) using 2,2,2-trichloroethyl *N*-(phenyl-λ²-iodanyl)sulfamate **2b** (60 mg, 0.14 mmol, 1.0 equiv.), cyclooctane **1d** (157 mg (190 µL), 1.4 mmol, 10.0 equiv.), iodosylbenzene (77 mg, 0.35 mmol, 2.5 equiv.) and 9*H*-thioxanthen-9-one (1.5 mg, 0.007 mmol, 5 mol%) in dry CHCl_3_ (1.5 mL, 0.1 M). The product was isolated by silica gel column chromatography using a gradient of petroleum ether/ethyl acetate (10:1) (33 mg, 0.097 mmol, 69%) as a colourless oil.

**^1^H NMR** (400 MHz, CDCl_3_) δ 4.89 – 4.75 (m, 1H), 4.60 (s, 2H), 3.74 – 3.61 (m, 1H), 2.06 – 1.92 (m, 2H), 1.77 – 1.61 (m, 4H), 1.53 – 1.43 (m, 8H) ppm.

**^13^C NMR** (101 MHz, CDCl_3_) δ 93.7, 78.2, 55.8, 32.5, 27.3, 25.3, 23.3 ppm.

**HRMS** (ESI) *m/z* calculated for C_10_H_18_Cl_3_KNO_3_S [M+K]^+^ 375.9704, found 375.9703.

**2,2,2-trifluoroethyl (1-methylcyclopentyl)sulfamate** (**3e**): ([***See spectra***](#e3))

**TLC**: R*_f_* = 0.25 (90:10 Petroleum ether: EtOAc).

The title compound **3e** was synthesized according to [**GP-C**](#GPC) using 2,2,2-trifluoroethyl *N*-(phenyl-λ²-iodanyl)sulfamate **2a** (54 mg, 0.14 mmol, 1.0 equiv.), methylcyclopentane **1e** (59 mg (79 µL), 0.7 mmol, 5.0 equiv.), iodosylbenzene (77 mg, 0.35 mmol, 2.5 equiv.) and 9*H*-thioxanthen-9-one (1.5 mg, 0.007 mmol, 5 mol%) in dry CHCl_3_ (1.5 mL, 0.1 M). The product was isolated by silica gel column chromatography using a gradient of petroleum ether/ethyl acetate (10:1). It was obtained as a mixture (18 mg, 0.069 mmol, 49%) as a colourless liquid.

**^1^H NMR** (400 MHz, CDCl_3_) δ 4.72 (s, 1H), 4.42 – 4.36 (m, 2H), 1.98 – 1.88 (m, 2H), 1.80 – 1.62 (m, 6H), 1.45 (s, 3H) ppm.

**^13^C NMR** (101 MHz, CDCl_3_) δ 122.4 (q, *J* = 277.5 Hz), 65.9, 65.1 (q, *J* = 37.8 Hz), 39.9, 25.9, 23.2 ppm.

**^19^F NMR** (376 MHz, CDCl_3_) δ -73.67 (t, *J* = 8.1 Hz) ppm.

**HRMS** (ESI) *m/z* calculated for C_8_H_14_F_3_NNaO_3_S [M+Na]^+^ 284.0571, found 284.0569.

***Comment****: We observed predominant 3°-amination (70%), with the remaining 30% corresponding to regioisomeric products bearing 2°-amination at various positions. These regioisomers could not be separated by column chromatography.*

**2,2,2-trifluoroethyl ((1r,4r)-1,4-dimethylcyclohexyl)sulfamate** (**3f**): ([***See spectra***](#h3))

**TLC**: R*_f_* = 0.25 (90:10 Petroleum ether: EtOAc).

The title compound **3f** was synthesized according to [**GP-C**](#GPC) using 2,2,2-trifluoroethyl *N*-(phenyl-λ²-iodanyl)sulfamate **2a** (54 mg, 0.14 mmol, 1.0 equiv.), (1*s*,4*s*)-1,4-dimethylcyclohexane **1f** (79 mg (102 µL), 0.7 mmol, 5.0 equiv.), iodosylbenzene (77 mg, 0.35 mmol, 2.5 equiv.) and 9*H*-thioxanthen-9-one (1.5 mg, 0.007 mmol, 5 mol%) in dry CHCl_3_ (1.5 mL, 0.1 M). The product was isolated by silica gel column chromatography using a gradient of petroleum ether/ethyl acetate (10:1) (19 mg, 0.066 mmol, 47%) as a colourless oil.

**^1^H NMR** (400 MHz, CDCl_3_) δ 4.73 (d, *J* = 14.6 Hz, 1H), 4.38 (q, *J* = 8.0 Hz, 2H), 1.85 (m, 2H), 1.62 (m, 4H), 1.46-1.38 (m, 4H), 1.16 – 1.05 (m, 2H), 0.91 (d, *J* = 6.6 Hz, 3H) ppm.

**^13^C NMR** (101 MHz, CDCl_3_) δ 122.4 (q, *J* = 277.5 Hz), 64.9 (q, *J* = 37.6 Hz), 58.7, 37.6, 31.7, 31.0, 22.8, 21.8 ppm.

**^19^F NMR** (376 MHz, CDCl_3_) δ -73.56 (t, *J* = 8.1 Hz) ppm.

**HRMS** (ESI) *m/z* calculated for C_10_H_18_F_3_NNaO_3_S [M+Na]^+^ 312.0850, found 312.0831.

**2,2,2-trichloroethyl ((1r,4r)-1,4-dimethylcyclohexyl)sulfamate** (**3f’**): ([***See spectra***](#h3))

**TLC**: R*_f_* = 0.25 (90:10 Petroleum ether: EtOAc).

The title compound **3f’** was synthesized according to [**GP-C**](#GPC) using 2,2,2-trichloroethyl *N*-(phenyl-λ²-iodanyl)sulfamate **2a** (60 mg, 0.14 mmol, 1.0 equiv.), (1*s*,4*s*)-1,4-dimethylcyclohexane **1f** (79 mg (102 µL), 0.7 mmol, 5.0 equiv.), iodosylbenzene (77 mg, 0.35 mmol, 2.5 equiv.) and 9*H*-thioxanthen-9-one (1.5 mg, 0.007 mmol, 5 mol%) in dry CHCl_3_ (1.5 mL, 0.1 M). The product was isolated by silica gel column chromatography using a gradient of petroleum ether/ethyl acetate (10:1) (20 mg, 0.066 mmol, 49%) as a colourless oil.

**^1^H NMR** (400 MHz, CDCl_3_) δ 4.61 (s, 2H), 4.56 (s, 1H), 1.95 – 1.81 (m, 2H), 1.71 – 1.59 (m, 4H), 1.43 (t, *J* = 0.7 Hz, 4H), 1.17 – 1.06 (m, 2H), 0.91 (d, *J* = 6.6 Hz, 3H) ppm.

**^13^C NMR** (101 MHz, CDCl_3_) δ 93.7, 78.3, 58.7, 37.8, 31.7, 31.0, 23.1, 21.8 ppm.

**HRMS** (ESI) *m/z* calculated for C_10_H_18_Cl_3_NNaO_3_S [M+Na]^+^ 312.0849, found 312.0852.

**2,2,2-trifluoroethyl ((1s,4s)-1,4-dimethylcyclohexyl)sulfamate** (**3g**): ([***See spectra***](#i3))

**TLC**: R*_f_* = 0.25 (90:10 Petroleum ether: EtOAc).

The title compound **3g** was synthesized according to [**GP-C**](#GPC) using 2,2,2-trifluoroethyl *N*-(phenyl-λ²-iodanyl)sulfamate **2a** (54 mg, 0.14 mmol, 1.0 equiv.), (1*r*,4*r*)-1,4-dimethylcyclohexane **1g** (79 mg (102 µL), 0.7 mmol, 5.0 equiv.), iodosylbenzene (77 mg, 0.35 mmol, 2.5 equiv.) and 9*H*-thioxanthen-9-one (1.5 mg, 0.007 mmol, 5 mol%) in dry CHCl_3_ (1.5 mL, 0.1 M). The product was isolated by silica gel column chromatography using a gradient of petroleum ether/ethyl acetate (10:1) (18 mg, 0.062 mmol, 45%) as a colourless oil. (d.r = 1.2:1 from crude ^1^H-NMR)

**^1^H NMR** (400 MHz, CDCl_3_) δ 4.52 (bs, 1H), 4.40 (q, *J* = 8.1 Hz, 2H), 1.66 – 1.51 (m, 1H), 1.40 (s, 3H), 1.33 (dd, *J* = 14.0, 3.6 Hz, 1H), 1.24 – 1.02 (m, 1H), 0.92 (d, *J* = 6.6 Hz, 3H) ppm.

**^13^C NMR** (101 MHz, CDCl_3_) δ 122.4 (q, *J* = 277.5 Hz), 64.8 (q, *J* = 37.7 Hz), 57.3, 37.6, 31.7, 30.1, 28.2, 22.2 ppm.

**^19^F NMR** (376 MHz, CDCl_3_) δ -73.62 (t, *J* = 8.0 Hz) ppm.

**HRMS** *m/z* calculated for C_10_H_18_F_3_NNaO_3_S [M+Na]^+^ 312.0849, found 312.0835.

**2,2,2-trifluoroethyl ((1r,4r)-1,4-dimethylcyclohexyl)sulfamate** (**3h**): ([***See spectra***](#g3))

**TLC**: R*_f_* = 0.25 (90:10 Petroleum ether: EtOAc).

The title compound **3h** was synthesized according to [**GP-C**](#GPC) using 2,2,2-trifluoroethyl *N*-(phenyl-λ²-iodanyl)sulfamate **2a** (54 mg, 0.14 mmol, 1.0 equiv.), *cis and trans* mixture of 1,4-dimethylcyclohexane **1h** (79 mg (102 µL), 0.7 mmol, 5.0 equiv.), iodosylbenzene (77 mg, 0.35 mmol, 2.5 equiv.) and 9*H*-thioxanthen-9-one (1.5 mg, 0.007 mmol, 5 mol%) in dry CHCl_3_ (1.5 mL, 0.1 M). The product was isolated by silica gel column chromatography using a gradient of petroleum ether/ethyl acetate (10:1) (16 mg, 0.055 mmol, 40%) as a colourless oil. (d.r = 3.4:1 from crude ^1^H-NMR)

**^1^H NMR** (400 MHz, CDCl_3_) δ 4.66 (s, 1H), 4.38 (q, *J* = 8.1 Hz, 2H), 1.90 – 1.81 (m, 2H), 1.63 (m, 4H), 1.48-1.35 (s, 4H), 1.16 – 1.06 (m, 2H), 0.91 (d, *J* = 6.6 Hz, 3H) ppm.

**^13^C NMR** (101 MHz, CDCl_3_) δ 122.4 (q, *J* = 277.5 Hz), 64.9 (q, *J* = 37.6 Hz), 58.7, 37.6, 31.7, 31.0, 22.8, 21.8 ppm.

**^19^F NMR** (376 MHz, CDCl_3_) δ -73.55 (t, *J* = 8.1 Hz) ppm.

**HRMS** (ESI) *m/z* calculated for C_10_H_18_F_3_NNaO_3_S [M+Na]^+^ 312.0851, found 312.0838.

**2,2,2-trifluoroethyl (1,3,5-trimethylcyclohexyl)sulfamate** (**3i**): ([***See spectra***](#j3))

**TLC**: R*_f_* = 0.25 (90:10 Petroleum ether: EtOAc).

The title compound **3i** was synthesized according to [**GP-C**](#GPC) using 2,2,2-trifluoroethyl *N*-(phenyl-λ²-iodanyl)sulfamate **2a** (54 mg, 0.14 mmol, 1.0 equiv.), 1,3,5-trimethylcyclohexane **1i** (88 mg (103 µL), 0.7 mmol, 5.0 equiv.), iodosylbenzene (77 mg, 0.35 mmol, 2.5 equiv.) and 9*H*-thioxanthen-9-one (1.5 mg, 0.007 mmol, 5 mol%) in dry CHCl_3_ (1.5 mL, 0.1 M). The product was isolated by silica gel column chromatography using a gradient of petroleum ether/ethyl acetate (10:1) (21 mg, 0.069 mmol, 50%) as a colourless oil. (d.r = 4:1 from crude ^1^H-NMR)

**^1^H NMR** (400 MHz, CDCl_3_) δ 4.56 (s, 1H), 4.39 (q, *J* = 8.1 Hz, 2H), 1.88 – 1.83 (m, 2H), 1.67 (m, 1H), 1.58 (m, 3H), 1.40 (s, 3H), 1.11 (m, 2H), 0.90 (d, *J* = 6.4 Hz, 6H) ppm.

**^13^C NMR** (101 MHz, CDCl_3_) δ 120.9 (q, *J* = 277.5 Hz), 64.8 (q, *J* = 37.6 Hz), 59.5, 46.1, 43.2, 29.7, 28.8, 23.5, 22.2 ppm.

**^19^F NMR** (376 MHz, CDCl_3_) δ -73.48 (t, *J* = 8.1 Hz) ppm.

**HRMS** (ESI) *m/z* calculated for C_11_H_20_F_3_NNaO_3_S [M+Na]^+^ 326.1008, found 326.0997.

**2,2,2-trifluoroethyl ((1R,2S,4S)-bicyclo[2.2.1]heptan-2-yl)sulfamate** (**3j**): ([***See spectra***](#k3))

**TLC**: R*_f_* = 0.25 (90:10 Petroleum ether: EtOAc).

The title compound **3j** was synthesized according to [**GP-C**](#GPC) using 2,2,2-trifluoroethyl *N*-(phenyl-λ²-iodanyl)sulfamate **2a** (54 mg, 0.14 mmol, 1.0 equiv.), bicyclo[2.2.1]heptane **1j** (67 mg (78 µL), 0.7 mmol, 5.0 equiv.), iodosylbenzene (77 mg, 0.35 mmol, 2.5 equiv.) and 9*H*-thioxanthen-9-one (1.5 mg, 0.007 mmol, 5 mol%) in dry CHCl_3_ (1.5 mL, 0.1 M). The product was isolated by silica gel column chromatography using a gradient of petroleum ether/ethyl acetate (10:1) (15 mg, 0.056 mmol, 40%) as a colourless oil.

**^1^H NMR** (400 MHz, CDCl_3_) δ 4.66 – 4.46 (m, 1H), 4.40 (q, *J* = 8.0 Hz, 2H), 3.39 (m, 1H), 2.38 (m, 1H), 2.31 (m, 1H), 1.83 (m, 1H), 1.59 – 1.45 (m, 2H), 1.36 – 1.30 (m, 1H), 1.25 (m, 2H), 1.20 – 1.09 (m, 2H) ppm.

**^13^C NMR** (101 MHz, CDCl_3_) δ 122.3 (q, *J* = 277.6 Hz), 65.1 (q, *J* = 37.7 Hz), 58.1, 42.4, 40.5, 35.8, 35.3, 28.1, 26.3 ppm.

**^19^F NMR** (376 MHz, CDCl_3_) δ -73.77 (t, *J* = 8.0 Hz) ppm.

**HRMS** (ESI) *m/z* calculated for C_9_H_13_F_3_NO_3_S [M-H]^-^ 272.0573, found 272.0576.

**2,2,2-trifluoroethyl ((2R,4aR,8aR)-decahydronaphthalen-2-yl)sulfamate** (**3k**): ([***See spectra***](#l3))

**TLC**: R*_f_* = 0.25 (90:10 Petroleum ether: EtOAc).

The title compound **3k** was synthesized according to [**GP-C**](#GPC) using 2,2,2-trifluoroethyl *N*-(phenyl-λ²-iodanyl)sulfamate **2a** (54 mg, 0.14 mmol, 1.0 equiv.), (4*as*,8*as*)-decahydronaphthalene **1k** (97 mg (108 µL), 0.7 mmol, 5.0 equiv.), iodosylbenzene (77 mg, 0.35 mmol, 2.5 equiv.) and 9*H*-thioxanthen-9-one (1.5 mg, 0.007 mmol, 5 mol%) in dry CHCl_3_ (1.5 mL, 0.1 M). The product was isolated by silica gel column chromatography using a gradient of petroleum ether/ethyl acetate (10:1) (29 mg, 0.092 mmol, 66%) as a white solid.

**^1^H NMR** (400 MHz, CDCl_3_) δ 4.47 (d, *J* = 7.7 Hz, 1H), 4.39 (q, *J* = 8.0 Hz, 2H), 3.36 (m, 1H), 2.14 – 2.07 (m, 1H), 1.98 (ddt, *J* = 12.0, 5.0, 2.4 Hz, 1H), 1.74 – 1.58 (m, 5H), 1.28 – 1.17 (m, 3H), 1.12 – 0.82 (m, 6H) ppm.

**^13^C NMR** (101 MHz, CDCl_3_) δ 122.3 (q, *J* = 277.6 Hz), 65.1 (q, *J* = 37.7 Hz), 54.6, 42.8, 41.8, 41.0, 33.9, 33.7, 33.2, 32.4, 26.5, 26.3 ppm.

**^19^F NMR** (376 MHz, CDCl_3_) δ -73.74 (t, *J* = 8.0 Hz) ppm.

**HRMS** (ESI) *m/z* calculated for C_12_H_19_F_3_NO_3_S [M-H]^-^ 314.0255, found 314.0252.

**2,2,2-trifluoroethyl ((3s,5s,7s)-adamantan-1-yl)sulfamate** (**3l**): ([***See spectra***](#m3))

**TLC**: R*_f_* = 0.25 (90:10 Petroleum ether: EtOAc).

The title compound **3l** was synthesized according to [**GP-C**](#GPC) using 2,2,2-trifluoroethyl *N*-(phenyl-λ²-iodanyl)sulfamate **2a** (54 mg, 0.14 mmol, 1.0 equiv.), adamantane **1l** (95 mg, 0.7 mmol, 5.0 equiv.), iodosylbenzene (77 mg, 0.35 mmol, 2.5 equiv.) and 9*H*-thioxanthen-9-one (1.5 mg, 0.007 mmol, 5 mol%) in dry CHCl_3_ (1.5 mL, 0.1 M). The product was isolated by silica gel column chromatography using a gradient of petroleum ether/ethyl acetate (10:1) (22 mg, 0.075 mmol, 54%) as a white solid.

**^1^H NMR** (400 MHz, CDCl_3_) δ 4.58 (s, 1H), 4.38 (q, *J* = 8.1 Hz, 2H), 2.16 – 2.10 (m, 3H), 1.94 (m, 6H), 1.71 – 1.63 (m, 6H) ppm.

**^13^C NMR** (101 MHz, CDCl_3_) δ 122.4 (q, *J* = 277.6 Hz), 65.0 (q, *J* = 37.7 Hz), 56.3, 42.5, 35.9, 29.7 ppm.

**^19^F NMR** (376 MHz, CDCl_3_) δ -73.48 (t, *J* = 8.1 Hz) ppm.

**HRMS** (ESI) *m/z* calculated for C_12_H_19_F_3_NO_3_S [M+H]^+^ 314.0956, found 314.0959.

**2,2,2-trichloroethyl ((3s,5s,7s)-adamantan-1-yl)sulfamate** (**3l’**): ([***See spectra***](#mm3))

**TLC**: Rf = 0.25 (90:10 Petroleum ether: EtOAc).

The title compound **3l’** was synthesized according to [**GP-C**](#GPC) using 2,2,2-trichloroethyl *N*-(phenyl-λ²-iodanyl)sulfamate **2b** (60 mg, 0.14 mmol, 1.0 equiv.), adamantane **1l** (95 mg, 0.7 mmol, 5.0 equiv.), iodosylbenzene (77 mg, 0.35 mmol, 2.5 equiv.) and 9*H*-thioxanthen-9-one (1.5 mg, 0.007 mmol, 5 mol%) in dry CHCl_3_ (1.5 mL, 0.1 M). The product was isolated by silica gel column chromatography using a gradient of petroleum ether/ethyl acetate (10:1) (26 mg, 0.075 mmol, 54%) as a white solid.

**^1^H NMR** (400 MHz, CDCl_3_) δ 4.62 (s, 2H), 4.49 (s, 1H), 2.17 – 2.09 (m, 3H), 1.99 (m, 6H), 1.72 – 1.63 (m, 6H) ppm.

**^13^C NMR** (101 MHz, CDCl_3_) δ 93.7, 78.3, 56.3, 42.7, 36.0, 29.7 ppm.

**HRMS** (ESI) *m/z* calculated for C_12_H_18_Cl_3_NNaO_3_S [M+Na]^+^ 384.6911, found 384.6920.

Spectral data were consistent with the literature.^[7]^

**2,2,2-trifluoroethyl ((1r,3R,5S,7r)-3,5-dimethyladamantan-1-yl)sulfamate** (**3m**): ([***See spectra***](#n3))

**TLC**: R*_f_* = 0.25 (90:10 Petroleum ether: EtOAc).

The title compound **3m** was synthesized according to [**GP-C**](#GPC) using 2,2,2-trifluoroethyl *N*-(phenyl-λ²-iodanyl)sulfamate **2a** (54 mg, 0.14 mmol, 1.0 equiv.), 1,3-dimethyladamantane **1m** (115 mg (130 µL), 0.7 mmol, 5.0 equiv.), iodosylbenzene (77 mg, 0.35 mmol, 2.5 equiv.) and 9*H*-thioxanthen-9-one (1.5 mg, 0.007 mmol, 5 mol%) in dry CHCl_3_ (1.5 mL, 0.1 M). The product was isolated by silica gel column chromatography using a gradient of petroleum ether/ethyl acetate (10:1) (25 mg, 0.073 mmol, 52%) as a colourless oil.

**^1^H NMR** (400 MHz, CDCl_3_) δ 4.82 – 4.66 (m, 1H), 4.37 (qd, *J* = 8.1, 1.1 Hz, 2H), 2.18 (p, *J* = 3.2 Hz, 1H), 1.78 (dd, *J* = 3.4, 1.3 Hz, 2H), 1.61 – 1.51 (m, 4H), 1.37 – 1.28 (m, 4H), 1.15 (t, *J* = 1.6 Hz, 2H), 0.86 (s, 6H) ppm.

**^13^C NMR** (101 MHz, CDCl_3_) δ 122.4 (q, *J* = 277.6 Hz), 65.0 (q, *J* = 37.6 Hz), 57.7, 50.2, 48.4, 42.2, 40.9, 32.9, 30.3, 30.0 ppm.

**^19^F NMR** (376 MHz, CDCl_3_) δ -73.45 (t, *J* = 8.0 Hz) ppm.

**HRMS** (ESI) *m/z* calculated for C_14_H_22_F_3_NNaO_3_S [M+Na]^+^ 364.1164, found 364.1154.

**2,2,2-trichloroethyl ((1r,3R,5S,7r)-3,5-dimethyladamantan-1-yl)sulfamate** (**3m’**):

([***See spectra***](#nn3))

**TLC**: R*_f_* = 0.25 (90:10 Petroleum ether: EtOAc).

The title compound **3m’** was synthesized according to [**GP-C**](#GPC) using 2,2,2-trichloroethyl *N*-(phenyl-λ²-iodanyl)sulfamate **2b** (60 mg, 0.14 mmol, 1.0 equiv.), 1,3-dimethyladamantane **1m** (115 mg (130 µL), 0.7 mmol, 5.0 equiv.), iodosylbenzene (77 mg, 0.35 mmol, 2.5 equiv.) and 9*H*-thioxanthen-9-one (1.5 mg, 0.007 mmol, 5 mol%) in dry CHCl_3_ (1.5 mL, 0.1 M). The product was isolated by silica gel column chromatography using a gradient of petroleum ether/ethyl acetate (10:1) (26 mg, 0.067 mmol, 48%) as a colourless oil.

**^1^H NMR** (400 MHz, CDCl_3_) δ 4.67 (s, 1H), 4.61 (s, 2H), 2.19 (p, *J* = 3.2 Hz, 1H), 1.87 – 1.79 (m, 2H), 1.67 – 1.57 (m, 4H), 1.37 – 1.28 (m, 4H), 1.18 – 1.12 (m, 2H), 0.86 (s, 6H) ppm.

**^13^C NMR** (101 MHz, CDCl_3_) δ 93.7, 78.3, 57.8, 50.2, 48.7, 42.3, 41.1, 32.9, 30.3, 30.1 ppm.

**HRMS** (ESI) *m/z* calculated for C_14_H_22_Cl_3_NNaO_3_S [M+Na]^+^ 412.0248, found 412.0251.

Spectral data were consistent with the literature.^[8]^

**2,2,2-trifluoroethyl ((1r,3s,5R,7S)-3-chloroadamantan-1-yl)sulfamate** (**3n**): ([***See spectra***](#o3))

**TLC**: R*_f_* = 0.25 (90:10 Petroleum ether: EtOAc).

The title compound **3n** was synthesized according to [**GP-C**](#GPC) using 2,2,2-trifluoroethyl *N*-(phenyl-λ²-iodanyl)sulfamate **2a** (54 mg, 0.14 mmol, 1.0 equiv.), 1-chloroadamantane **1n** (119 mg, 0.7 mmol, 5.0 equiv.), iodosylbenzene (77 mg, 0.35 mmol, 2.5 equiv.) and 9*H*-thioxanthen-9-one (1.5 mg, 0.007 mmol, 5 mol%) in dry CHCl_3_ (1.5 mL, 0.1 M). The product was isolated by silica gel column chromatography using a gradient of petroleum ether/ethyl acetate (10:1) (20 mg, 0.058 mmol, 41%) as a colourless oil.

**^1^H NMR** (400 MHz, CDCl_3_) δ 4.57 (s, 1H), 4.41 (q, *J* = 8.1 Hz, 2H), 2.51 (s, 2H), 2.33 – 2.21 (m, 6H), 1.95 (d, *J* = 3.2 Hz, 4H), 1.69 – 1.62 (m, 2H) ppm.

**^13^C NMR** (101 MHz, CDCl_3_) δ 122.3 (q, *J* = 277.6 Hz), 65.1 (q, *J* = 37.7 Hz), 61.3, 57.8, 53.3, 47.4, 40.5, 34.0, 32.4 ppm.

**^19^F NMR** (376 MHz, CDCl_3_) δ -73.41 (t, *J* = 8.1 Hz) ppm.

**HRMS** (ESI) *m/z* calculated for C_12_H_17_ClF_3_NNaO_3_S [M+Na]^+^ 370.0579, found 370.0580.

**2,2,2-trifluoroethyl ((1r,3s,5R,7S)-3-bromoadamantan-1-yl)sulfamate** (**3o**): ([***See spectra***](#p3))

**TLC**: R*_f_* = 0.25 (90:10 Petroleum ether: EtOAc).

The title compound **3o** was synthesized according to [**GP-C**](#GPC) using 2,2,2-trifluoroethyl *N*-(phenyl-λ²-iodanyl)sulfamate **2a** (54 mg, 0.14 mmol, 1.0 equiv.), 1-bromoadamantane **1o** (150 mg, 0.7 mmol, 5.0 equiv.), iodosylbenzene (77 mg, 0.35 mmol, 2.5 equiv.) and 9*H*-thioxanthen-9-one (1.5 mg, 0.007 mmol, 5 mol%) in dry CHCl_3_ (1.5 mL, 0.1 M). The product was isolated by silica gel column chromatography using a gradient of petroleum ether/ethyl acetate (10:1) (28 mg, 0.071 mmol, 51%) as a brown oil.

**^1^H NMR** (400 MHz, CDCl_3_) δ 4.62 (s, 1H), 4.40 (q, *J* = 8.1 Hz, 2H), 2.50 (s, 2H), 2.30 – 2.20 (m, 6H), 1.94 (d, *J* = 3.1 Hz, 4H), 1.68 – 1.61 (m, 2H) ppm.

**^13^C NMR** (101 MHz, CDCl_3_) δ 122.1 (q, *J* = 277.6 Hz), 64.9 (q, *J* = 37.8 Hz), 61.2, 57.6, 53.1, 47.2, 40.3, 33.8, 32.2, 14.1 ppm.

**^19^F NMR** (376 MHz, CDCl_3_) δ -73.36 (t, *J* = 8.1 Hz) ppm.

**HRMS** (ESI) *m/z* calculated for C_12_H_17_BrF_3_NNaO_3_S [M+Na]^+^ 414.0095, found 414.0090.

**2,2,2-trichloroethyl ((1r,3s,5R,7S)-3-bromoadamantan-1-yl)sulfamate** (**3o’**): ([***See spectra***](#p3))

**TLC**: R*_f_* = 0.25 (90:10 Petroleum ether: EtOAc).

The title compound **3o’** was synthesized according to [**GP-C**](#GPC) using 2,2,2-trifluoroethyl *N*-(phenyl-λ²-iodanyl)sulfamate **2b** (60 mg, 0.14 mmol, 1.0 equiv.), 1-bromoadamantane **1o** (150 mg, 0.7 mmol, 5.0 equiv.), iodosylbenzene (77 mg, 0.35 mmol, 2.5 equiv.) and 9*H*-thioxanthen-9-one (1.5 mg, 0.007 mmol, 5 mol%) in dry CHCl_3_ (1.5 mL, 0.1 M). The product was isolated by silica gel column chromatography using a gradient of petroleum ether/ethyl acetate (10:1) (34 mg, 0.077 mmol, 55%) as a pale brown solid.

**^1^H NMR** (400 MHz, CDCl_3_) δ 4.92 (bs, 1H), 4.62 (s, 2H), 2.57 (s, 2H), 2.31 – 2.19 (m, 6H), 2.02 – 1.96 (m, 4H), 1.68 – 1.61 (m, 2H) ppm.

**^13^C NMR** ^13^C NMR (101 MHz, CDCl_3_) δ 93.5, 78.4, 61.7, 57.8, 53.4, 47.4, 40.6, 34.0, 32.3 ppm.

**HRMS** (ESI) *m/z* calculated for C_12_H_17_BrCl_3_NNaO_3_S [M+Na]^+^ 462.0119, found 462.0122.

**2,2,2-trifluoroethyl ((1R,2R)-2-iodocyclopentyl)sulfamate** (**3p**): ([***See spectra***](#q3))

**TLC**: R*_f_* = 0.15 (90:10 Petroleum ether: EtOAc).

The title compound **3p** was synthesized according to [**GP-C**](#GPC) using 2,2,2-trifluoroethyl *N*-(phenyl-λ²-iodanyl)sulfamate **2a** (54 mg, 0.14 mmol, 1.0 equiv.), iodocyclopentane **1p** (137 mg, 0.7 mmol, 5.0 equiv.), iodosylbenzene (77 mg, 0.35 mmol, 2.5 equiv.) and 9*H*-thioxanthen-9-one (1.5 mg, 0.007 mmol, 5 mol%) in dry CHCl_3_ (1.5 mL, 0.1 M). The product was isolated by silica gel column chromatography using a gradient of petroleum ether/ethyl acetate (20:1) (34 mg, 0.091 mmol, 65%) as a white solid.

**^1^H NMR** (400 MHz, CDCl_3_) δ 4.90 (d, *J* = 9.2 Hz, 1H), 4.58 – 4.55 (m, 1H), 4.38 (qt, *J* = 8.0, 4.1 Hz, 2H), 3.04 – 2.96 (m, 1H), 2.23 (m, 2H), 2.02 – 1.90 (m, 2H), 1.72 – 1.62 (m, 2H) ppm.

**^13^C NMR** (101 MHz, CDCl_3_) δ 122.2 (q, *J* = 277.6 Hz), 65.4 (q, *J* = 37.8 Hz), 59.2, 40.0, 35.5, 29.1, 19.8 ppm.

**^19^F NMR** (376 MHz, CDCl_3_) δ -73.73 (t, *J* = 8.0 Hz) ppm.

**HRMS** (APCI) *m/z* calculated for C_7_H_12_F_3_INNaO_3_S [M+H]^+^ 373.9491, found 373.9490.

**2,2,2-trifluoroethyl ((1R,2R)-2-(trifluoromethyl)cyclohexyl)sulfamate** (**3q**): ([***See spectra***](#r3))

**TLC**: R*_f_* = 0.25 (90:10 Petroleum ether: EtOAc).

The title compound **3q** was synthesized according to [**GP-C**](#GPC) using 2,2,2-trifluoroethyl *N*-(phenyl-λ²-iodanyl)sulfamate **2a** (54 mg, 0.14 mmol, 1.0 equiv.), (trifluoromethyl)cyclohexane **1q** (106 mg (97 µL), 0.7 mmol, 5.0 equiv.), iodosylbenzene (77 mg, 0.35 mmol, 2.5 equiv.) and 9*H*-thioxanthen-9-one (1.5 mg, 0.007 mmol, 5 mol%) in dry CHCl_3_ (1.5 mL, 0.1 M). The product was isolated by silica gel column chromatography using a gradient of petroleum ether/ethyl acetate (10:1) (14 mg, 0.043 mmol, 30%) as a colourless oil.

**^1^H NMR** (400 MHz, CDCl_3_) δ 4.81 (bs, 1H), 4.51 (q, *J* = 7.9 Hz, 1H), 3.37 (m, 1H), 2.33 (m, 1H), 2.18 – 2.10 (m, 2H), 1.98 – 1.89 (m, 2H), 1.41 – 1.28 (m, 2H), 1.23 – 1.18 (m, 2H) ppm.

**^13^C NMR** (101 MHz, CDCl_3_) δ 126.9 (q, *J* = 278.5 Hz), 122.2 (q, *J* = 277.5 Hz), 65.3 (q, *J* = 37.8 Hz), 53.2, 41.3 (q, *J* = 27.5 Hz), 32.9, 32.2 (q, *J* = 2.7 Hz), 23.9 (q, *J* = 2.5 Hz), 23.4 ppm.

**^19^F NMR** (376 MHz, CDCl_3_) δ -73.66 (t, *J* = 8.1 Hz) ppm.

**HRMS** (ESI) *m/z* calculated for C_9_H_12_F_6_NO_3_S [M-H]^-^ 328.0447, found 328.0454.

***Note****: The product is unstable during [NMR/HRMS/etc.] analysis and shows signs of decomposition.*

**2,2,2-trifluoroethyl ((1R,2R)-2-chlorocyclohexyl)sulfamate** (**3r**): ([***See spectra***](#s3))

**TLC**: R*_f_* = 0.15 (90:10 Petroleum ether: EtOAc).

The title compound **3r** was synthesized according to [**GP-C**](#GPC) using 2,2,2-trifluoroethyl *N*-(phenyl-λ²-iodanyl)sulfamate **2a** (54 mg, 0.14 mmol, 1.0 equiv.), chlorocyclohexane **1r** (83 mg (83 µL), 0.7 mmol, 5.0 equiv.), iodosylbenzene (77 mg, 0.35 mmol, 2.5 equiv.) and 9*H*-thioxanthen-9-one (1.5 mg, 0.007 mmol, 5 mol%) in dry CHCl_3_ (1.5 mL, 0.1 M). The product was isolated by silica gel column chromatography using a gradient of petroleum ether/ethyl acetate (20:1) (21 mg, 0.071 mmol, 51%) as a white solid.

**^1^H NMR** (400 MHz, CDCl_3_) δ 4.93 (m, 1H), 4.81 (d, *J* = 9.3 Hz, 1H), 4.71 – 4.63 (m, 2H), 2.70 (ddt, *J* = 12.7, 9.2, 3.5 Hz, 1H), 2.23 – 2.16 (m, 1H), 1.94 – 1.76 (m, 1H)., 1.74 – 1.59 (m, 3H), 1.46 – 1.37 (m, 1H) ppm.

**^13^C NMR** (101 MHz, CDCl_3_) δ 122.2 (q, *J* = 277.6 Hz), 65.4 (q, *J* = 37.8 Hz), 56.4, 35.1, 30.3, 24.4, 21.4 ppm.

**^19^F NMR** (376 MHz, CDCl_3_) δ -73.72 (t, *J* = 8.0 Hz) ppm.

**HRMS** (APCI) *m/z* calculated for C_8_H_14_ClF_3_NO_3_S [M+H]^+^ 296.0335, found 296.0340.

**2,2,2-trifluoroethyl ((1R,2R)-2-bromocyclohexyl)sulfamate** (**3s**): ([***See spectra***](#t3))

**TLC**: R*_f_* = 0.15 (90:10 Petroleum ether: EtOAc).

The title compound **3s** was synthesized according to [**GP-C**](#GPC) using 2,2,2-trifluoroethyl *N*-(phenyl-λ²-iodanyl)sulfamate **2a** (54 mg, 0.14 mmol, 1.0 equiv.), bromocyclohexane **1s** (114 mg (86 µL), 0.7 mmol, 5.0 equiv.), iodosylbenzene (77 mg, 0.35 mmol, 2.5 equiv.) and 9*H*-thioxanthen-9-one (1.5 mg, 0.007 mmol, 5 mol%) in dry CHCl_3_ (1.5 mL, 0.1 M). The product was isolated by silica gel column chromatography using a gradient of petroleum ether/ethyl acetate (20:1) (18 mg, 0.053 mmol, 38%) as a brown solid.

**^1^H NMR** (400 MHz, CDCl_3_) δ 4.58 (d, *J* = 7.9 Hz, 1H), 4.52 (d, *J* = 3.3 Hz, 1H), 4.42 (d, *J* = 8.0 Hz, 2H), 3.45 (td, *J* = 6.1, 3.0 Hz, 1H), 2.16 – 2.09 (m, 2H), 1.97 – 1.84 (m, 6H) ppm.

**^13^C NMR** (101 MHz, CDCl_3_) δ 122.2 (q, *J* = 277.6 Hz), 65.4 (q, *J* = 37.8 Hz), 56.4, 35.2, 30.4, 24.8, 21.5, 18.7, 11.4 ppm.

**^19^F NMR** (376 MHz, CDCl_3_) δ -73.74 (t, *J* = 8.0 Hz) ppm.

**HRMS** (APCI) *m/z* calculated for C_8_H_14_BrF_3_NO_3_S [M+H]^+^ 339.9916, found 339.9918.

**2,2,2-trifluoroethyl ((1R,2R)-2-iodocyclohexyl)sulfamate** (**3t**): ([***See spectra***](#u3))

**TLC**: R*_f_* = 0.15 (90:10 Petroleum ether: EtOAc).

The title compound **3t** was synthesized according to [**GP-C**](#GPC) using 2,2,2-trifluoroethyl *N*-(phenyl-λ²-iodanyl)sulfamate **2a** (54 mg, 0.14 mmol, 1.0 equiv.), iodocyclohexane **1t** (147 mg (90 µL), 0.7 mmol, 5.0 equiv.), iodosylbenzene (77 mg, 0.35 mmol, 2.5 equiv.) and 9*H*-thioxanthen-9-one (1.5 mg, 0.007 mmol, 5 mol%) in dry CHCl_3_ (1.5 mL, 0.1 M). The product was isolated by silica gel column chromatography using a gradient of petroleum ether/ethyl acetate (20:1) (30 mg, 0.077 mmol, 55%) as a white solid.

**^1^H NMR** (400 MHz, CDCl_3_) δ 4.93 – 4.80 (m, 2H), 4.44 (qd, *J* = 8.0, 4.8 Hz, 2H), 2.69 – 2.60 (m, 1H), 2.19 (dddd, *J* = 13.4, 5.5, 3.4, 2.0 Hz, 1H), 1.82 – 1.74 (m, 3H), 1.70 – 1.59 (m, 3H), 1.43 (m, 1H) ppm.

**^13^C NMR** (101 MHz, CDCl_3_) δ 122.2 (q, *J* = 277.6 Hz), 65.4 (q, *J* = 37.8 Hz), 56.4, 35.1, 30.3, 24.7, 21.5, 18.6 ppm.

**^19^F NMR** (376 MHz, CDCl_3_) δ -73.70 (t, *J* = 8.0 Hz) ppm.

**HRMS** (APCI) *m/z* calculated for C_8_H_14_F_3_INO_3_S [M+H]^+^ 387.9799, found 387.9801.

**2,2,2-trichloroethyl ((1R,2R)-2-iodocyclohexyl)sulfamate** (**3t’**): ([***See spectra***](#uu3))

The molecular structure was confirmed by X-ray crystallography (**CCDC 2190559**)

**TLC**: R*_f_* = 0.15 (90:10 Petroleum ether: EtOAc).

The title compound **3t’** was synthesized according to [**GP-C**](#GPC) using 2,2,2-trichloroethyl *N*-(phenyl-λ²-iodanyl)sulfamate **2b** (60 mg, 0.14 mmol, 1.0 equiv.), iodocyclohexane **1t** (147 mg (90 µL), 0.7 mmol, 5.0 equiv.), iodosylbenzene (77 mg, 0.35 mmol, 2.5 equiv.) and 9*H*-thioxanthen-9-one (1.5 mg, 0.007 mmol, 5 mol%) in dry CHCl_3_ (1.5 mL, 0.1 M). The product was isolated by silica gel column chromatography using a gradient of petroleum ether/ethyl acetate (20:1) (35 mg, 0.080 mmol, 57%) as a white solid.

**^1^H NMR** (400 MHz, CDCl_3_) δ 4.93 (m, 1H), 4.81 (d, *J* = 9.0 Hz, 1H), 4.71 – 4.63 (m, 2H), 2.80 – 2.62 (m, 1H), 2.26 – 2.15 (m, 1H), 1.90 – 1.74 (m, 1H), 1.71 – 1.59 (m, 3H), 1.46 – 1.36 (m, 1H) ppm.

**^13^C NMR** (101 MHz, CDCl_3_) δ 93.5, 78.5, 56.4, 44.4, 35.2, 30.5, 24.8, 21.5 ppm.

**HRMS** (APCI) *m/z* calculated for C_8_H_14_Cl_3_INO_3_S [M+H]^+^ 435.9221, found 435.9220.

**2,2,2-trifluoroethyl pentanylsulfamate** (**3u**): ([***See spectra***](#uu3))

**TLC**: R*_f_* = 0.25 (90:10 Petroleum ether: EtOAc).

The title compound **3u** was synthesized according to [**GP-C**](#GPC) using 2,2,2-trifluoroethyl *N*-(phenyl-λ²-iodanyl)sulfamate **2a** (191 mg, 0.5 mmol, 1.0 equiv.), pentane **1u** (360 mg (580 µL), 5.0 mmol, 10.0 equiv.), iodosylbenzene (275 mg, 1.25 mmol, 2.5 equiv.) and 9*H*-thioxanthen-9-one (5.3 mg, 0.025 mmol, 5 mol%) in dry CHCl_3_ (5.0 mL, 0.1 M). The product was isolated by silica gel column chromatography using a gradient of petroleum ether/ethyl acetate (10:1) (45 mg, 0.18 mmol, 36%) as a colourless oil.

**^1^H NMR** (400 MHz, CDCl_3_) δ 4.57 – 4.45 (m, 1H), 4.40 (qd, *J* = 8.1, 2.1 Hz, 2H), 3.62 – 3.49 (m, 1H), 1.70 – 1.31 (m, 5H), 1.25 (d, *J* = 6.6 Hz, 3H), 0.94 (dt, *J* = 8.1, 7.3 Hz, 4H) ppm.

**^13^C NMR** (101 MHz, CDCl_3_) δ 122.3 (q, *J* = 277.6 Hz), 67.9 – 61.9 (m), 58.7, 51.8, 39.3, 29.9, 27.1, 21.4, 19.0, 13.8 ppm.

**^19^F NMR** (376 MHz, CDCl_3_) δ -73.8 (t, *J* = 8.0 Hz), -73.9 (t, *J* = 8.0 Hz) ppm.

**HRMS** (APCI) *m/z* calculated for C_7_H_15_F_3_NO_3_S [M+H]^+^ 250.0861, found 250.0862.

***Note****: Amination of pentane gave a mixture of inseparable isomers; the reaction was conducted on a 0.5 mmol scale due to purification challenges.*

**2,2,2-trifluoroethyl hexanylsulfamate** (**3v**): ([***See spectra***](#uu3))

**TLC**: R*_f_* = 0.25 (90:10 Petroleum ether: EtOAc).

The title compound **3v** was synthesized according to [**GP-C**](#GPC) using 2,2,2-trifluoroethyl *N*-(phenyl-λ²-iodanyl)sulfamate **2a** (191 mg, 0.5 mmol, 1.0 equiv.), pentane **1v** (430 mg (650 µL), 5.0 mmol, 10.0 equiv.), iodosylbenzene (275 mg, 1.25 mmol, 2.5 equiv.) and 9*H*-thioxanthen-9-one (5.3 mg, 0.025 mmol, 5 mol%) in dry CHCl_3_ (5.0 mL, 0.1 M). The product was isolated by silica gel column chromatography using a gradient of petroleum ether/ethyl acetate (10:1) (40 mg, 0.16 mmol, 32%) as a colourless oil.

**^1^H NMR** (400 MHz, CDCl_3_) δ 4.68 – 4.49 (m, 1H), 4.40 (qd, *J* = 8.1, 0.8 Hz, 2H), 3.54 (ddq, *J* = 13.3, 8.5, 6.6 Hz, 1H), 1.69 – 1.41 (m, 4H), 1.41 – 1.27 (m, 4H), 1.27 – 1.20 (m, 3H), 1.00 – 0.80 (m, 5H) ppm.

**^13^C NMR** (101 MHz, CDCl_3_) δ 122.2 (q, *J* = 277.5 Hz), 64.9 (qd, *J* = 37.8, 3.9 Hz), 60.5, 57.1, 51.9, 36.7, 36.3, 29.7, 27.5, 21.2, 21.1, 18.7, 14.2, 13.8, 9.5 ppm.

**^19^F NMR** (376 MHz, CDCl_3_) δ -73.75 – 73.98 (m) ppm.

**HRMS** (APCI) *m/z* calculated for C_8_H_17_F_3_NO_3_S [M+H]^+^ 264.0922, found 264.0925.

***Note****: Amination of hexane gave a mixture of inseparable isomers; the reaction was conducted on a 0.5 mmol scale due to purification challenges.*

## 4.2 Limitations (Unsuccessful substrates):

# 5. Gram-Scale synthesis:

To a 100 mL round bottom flask equipped with a Teflon-coated magnetic stir bar was added iminoiodinane (1.03 g, 2.4 mmol, 1.0 equiv.), iodosyl benzene (1.32 g, 6.0 mmol, 2.5 equiv.) and 9*H*-thioxanthen-9-one (25.5 mg, 0.12 mmol, 5 mol%) in dry CHCl_3_ (24 mL, 0.1 M). The resulting mixture was degassed *via* ‘freeze-pump-thaw’ procedure (3 times). Subsequently, cyclohexane (2.62 mL, 24.0 mmol, 10.0 equiv.) was added against the positive flow of nitrogen. The reaction mixture was then stirred under irradiation with LEDs (2 x 40 W, λ_max_ = 370 nm) at room temperature (35 °C to 38 °C) for 16 h. Upon completion of the reaction, the crude product was washed with sodium thiosulfate solution (2 x 20 mL) and the organic layer was dried over MgSO_4_. The solvent was removed by vacuum and the crude product was purified by column chromatography on silica gel silica: 60; eluant: petroleum ether/ethyl acetate (10:1) to provide pure product as a white solid (388 mg, 1.25 mmol, 52%).


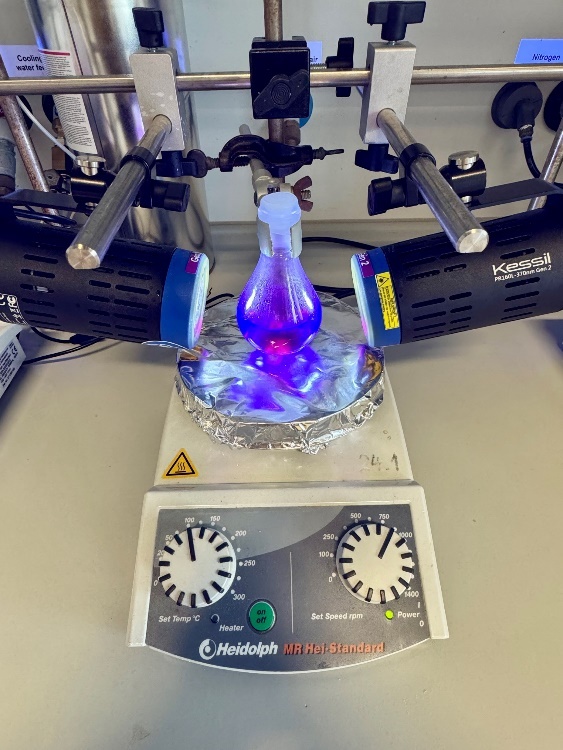


**Figure S2**: Reaction setup for gram-scale synthesis

# 6. Investigations of reaction conditions on Intra-molecular amination:

**Initial reaction with sulfamate and Iodosobenzene:**

**Reaction with isopentyl (phenyl-λ^3^-iodaneylidene)sulfamate and PhIO:**

***Comment:*** *We observed that performing the reaction with isopentyl (phenyl-λ³-iodaneylidene)sulfamate resulted in a 63% yield, while generating phenyl-λ³-iodaneylidene in situ gave a comparable 62% yield. Given the similar outcomes, we proceeded with the direct use of the sulfamate.*

**Procedure**: All reactions were performed on 0.2 mmol scale. To an 10 mL Schlenk tube equipped with a Teflon coated magnetic stir bar, 3-methylbutyl sulfamate **4a** (33.4 mg, 0.2 mmol, 1.0 equiv.), Iodosyl benzene (176 mg, 0.8 mmol, 4.0 equiv.) and photocatalyst (2.1 mg, 0.01 mmol, 5 mol%) were added. The Schlenk tube was evacuated and backfilled with nitrogen three times. Subsequently, solvent (2 mL) was added against the positive flow of nitrogen. The reaction mixture was then stirred under irradiation with LEDs (2 x 40 W, λ_max_ = 370 nm). Later, the solvent was removed in *vacuo*. Mesitylene (27.71 µL, 0.2 mmol) was added as internal standard, and the yield was determined by crude ^1^H NMR analysis.

**Table S6. Intra-molecular reaction optimiztion.**^[a]^

| Entry | Conditions | Solvent | Yield of 5a (%)^[b]^ |
| --- | --- | --- | --- |
| 1^[c]^ | *fac*-Ir(ppy)_3_ | CHCl_3_ | <5 |
| 2^[c]^ | Ir[dFFppy]_2_-(4,4′-dCF_3_bpy)PF_6_ | CHCl_3_ | <10 |
| 3 | 4CzIPN | CHCl_3_ | <10 |
| 4 | Eosin Y | CHCl_3_ | <10 |
| 5 | Ru(bpy)_3_Cl_2_ | CHCl_3_ | <10 |
| 6 | Mes.Acr.MeClO_4_ | CHCl_3_ | <10 |
| 7 | TBADT | CHCl_3_ | 44 |
| 8 | 2-CF_3_TXO | CHCl_3_ | 22 |
| 9 | 2,2’-BrTXO | CHCl_3_ | 20 |
| 10 | 2,2’-OMeTXO | CHCl_3_ | 25 |
| 11 | 2-ITXO | CHCl_3_ | 39 |
| 12 | 4-OMeTXO | CHCl_3_ | 40 |
| 13 | **TXO** | **CHCl_3_** | **62** |
| 14 | TXO | DCM | 56 |
| 15^[d]^ | TXO | CHCl_3_ | 33 |
| 16^[e]^ | TXO | CHCl_3_ | 49 |
| 17 | w/o TXO | CHCl_3_ | n.d |
| 18 | w/o TXO in dark | CHCl_3_ | n.d |

^[a]^**Reaction conditions**: **4a** Sulfamate (0.2 mmol), Iodosyl benzene (4.0 equiv.), and PC (5.0 mol%) in dry solvent (0.1 M), irradiation with LEDs (λ_max_ = 370 nm) under nitrogen atmosphere at room temperature for 16 h. ^[b]^Determined by crude NMR using mesitylene as internal standard. ^[c]^2.0 mol% has been used. ^[d]^2.0 equiv. of PhIO has been used. ^[e]^3.0 equiv. of PhIO has been used.

## 6.1 General Procedure D (GP-D)

To an 25 mL Schlenk tube equipped with a Teflon coated magnetic stir bar was added sulfamate (0.5 mmol, 1.0 equiv.), iodosylbenzene (2.0 mmol, 4.0 equiv.) and 9*H*-thioxanthen-9-one (5 mol%) in dry CHCl_3_ (0.1 M). The resulting mixture was degassed *via* ‘freeze-pump-thaw’ procedure (3 times). The reaction mixture was then stirred for 15 minutes at room temperature then irradiate with LEDs (2 x 40 W, λ_max_ = 370 nm) at room temperature (35 °C to 38 °C) for 16 h. Upon completion of the reaction, the crude product was washed with sodium thiosulfate solution (2 x 10 mL) and the organic layer was dried over MgSO_4_. The solvent was removed by vacuum and the crude product was purified by column chromatography on silica gel silica: 60; eluant: petroleum ether/ethyl acetate.

## 6.2 Substrate Scope:

**4,4-Dimethyl-1,2,3-oxathiazinane 2,2-dioxide** (**5a**): ([***See spectra***](#a5))

**TLC**: R*_f_* = 0.25 (80:20 Petroleum ether: EtOAc).

The title compound **5a** was synthesized according to [**GP-D**](#GPD) using 3-methylbutyl sulfamate **4a** (84 mg, 0.5 mmol, 1.0 equiv.), iodosylbenzene (440 mg, 2.0 mmol, 4.0 equiv.) and 9*H*-thioxanthen-9-one (5.3 mg, 0.025 mmol, 5 mol%) in dry CHCl_3_ (5.0 mL, 0.1 M). The product was isolated by silica gel column chromatography using a gradient of petroleum ether/ethyl acetate (9:1) (51 mg, 0.31 mmol, 62%) as a colourless oil.

**^1^H NMR** (400 MHz, CDCl_3_) δ 5.22 – 4.49 (m, 2H), 4.25 (s, 1H), 1.86 – 1.65 (m, 2H), 1.41 (s, 6H) ppm.

**^13^C NMR** (101 MHz, CDCl_3_) δ 69.4, 56.6, 35.5, 28.3 ppm.

**HRMS** (ESI) *m/z* calculated for C_5_H_11_NNaO_3_S [M+Na]^+^ 188.0351, found 188.0350.

Spectral data were consistent with the literature.^[12]^

**4,4-Dimethyl-6-propyl-1,2,3-oxathiazinane 2,2-dioxide** (**5b**): ([***See spectra***](#b5))

**TLC**: R*_f_* = 0.25 (80:20 Petroleum ether: EtOAc).

The title compound **5b** was synthesized according to [**GP-D**](#GPD) using 2-methylheptan-4-yl sulfamate **4b** (105 mg, 0.5 mmol, 1.0 equiv.), iodosylbenzene (440 mg, 2.0 mmol, 4.0 equiv.) and 9*H*-thioxanthen-9-one (5.3 mg, 0.025 mmol, 5 mol%) in dry CHCl_3_ (5.0 mL, 0.1 M). The product was isolated by silica gel column chromatography using a gradient of petroleum ether/ethyl acetate (9:1) (61 mg, 0.29 mmol, 59%) as a colourless oil.

**^1^H NMR** (400 MHz, CDCl_3_) δ 4.76 (tdd, *J* = 8.9, 6.1, 2.9 Hz, 1H), 4.24 (bs, 1H), 1.73 – 1.63 (m, 1H), 1.63 – 1.53 (m, 2H), 1.53 – 1.24 (m, 6H), 1.23 (m, 3H), 0.91 – 0.85 (m, 3H) ppm.

**^13^C NMR** (101 MHz, CDCl_3_) δ 81.2, 55.9, 41.4, 37.3, 31.8, 25.2, 17.9, 13.7 ppm.

**HRMS** (ESI) *m/z* calculated for C_8_H_17_NNaO_3_S [M+Na]^+^ 230.0936, found 230.0936.

Spectral data were consistent with the literature.^[13]^

**6-Butyl-4,4-dimethyl-1,2,3-oxathiazinane 2,2-dioxide** (**5c**): ([***See spectra***](#c5))

**TLC**: R*_f_* = 0.25 (80:20 Petroleum ether: EtOAc).

The title compound **5c** was synthesized according to [**GP-D**](#GPD) using 2-methyloctan-4-yl sulfamate **4c** (111 mg, 0.5 mmol, 1.0 equiv.), iodosylbenzene (440 mg, 2.0 mmol, 4.0 equiv.) and 9*H*-thioxanthen-9-one (5.3 mg, 0.025 mmol, 5 mol%) in dry CHCl_3_ (5.0 mL, 0.1 M). The product was isolated by silica gel column chromatography using a gradient of petroleum ether/ethyl acetate (9:1) (53 mg, 0.24 mmol, 48%) as a colourless oil.

**^1^H NMR** (400 MHz, CDCl_3_) δ 4.76 (m, 1H), 4.24 (bs, 1H), 1.73 – 1.63 (m, 1H), 1.63 – 1.53 (m, 3H), 1.53 – 1.24 (m, 7H), 1.23 (m, 3H), 0.91 – 0.85 (m, 3H) ppm.

**^13^C NMR** (101 MHz, CDCl_3_) δ 81.5, 56.0, 41.5, 35.0, 31.9, 26.8, 25.3, 22.4, 14.0 ppm.

**HRMS** (ESI) *m/z* calculated for C_9_H_19_NNaO_3_S [M+Na]^+^ 244.1255, found 244.1257.

Spectral data were consistent with the literature.^[14]^

**(4*aR*,7*S*,8a*S*)-4,4,7-trimethyloctahydrobenzo[*e*][1,2,3]oxathiazine 2,2-dioxide** (**5d**): ([***See spectra***](#d5))

**TLC**: R*_f_* = 0.25 (80:20 Petroleum ether: EtOAc).

The title compound **5c** was synthesized according to [**GP-D**](#GPD) using 2-methyloctan-4-yl sulfamate **4c** (118 mg, 0.5 mmol, 1.0 equiv.), iodosylbenzene (440 mg, 2.0 mmol, 4.0 equiv.) and 9*H*-thioxanthen-9-one (5.3 mg, 0.025 mmol, 5 mol%) in dry CHCl_3_ (5.0 mL, 0.1 M). The product was isolated by silica gel column chromatography using a gradient of petroleum ether/ethyl acetate (9:1) (71 mg, 0.30 mmol, 61%) as a white solid.

**^1^H NMR** (400 MHz, CDCl_3_) δ 4.59 (td, *J* = 10.9, 4.5 Hz, 1H), 4.20 (s, 1H), 2.11 (m, 1H), 1.83 – 1.73 (m, 2H), 1.60 – 1.52 (m, 1H), 1.45 (ddd, *J* = 12.0, 10.5, 3.3 Hz, 1H), 1.38 (s, 3H), 1.31 – 1.22 (m, 4H), 1.14 – 1.03 (m, 1H), 1.03 – 0.95 (m, 4H) ppm.

**^13^C NMR** (101 MHz, CDCl_3_) δ 82.4, 59.2, 49.1, 40.5, 34.2, 31.5, 29.8, 25.3, 21.9, 21.2 ppm.

**HRMS** (ESI) *m/z* calculated for C_10_H_20_NO_3_S [M+H]^+^ 234.1160, found 234.1160.

Spectral data were consistent with the literature.^[15]^

**6-(but-3-en-1-yl)-4,4-dimethyl-1,2,3-oxathiazinane 2,2-dioxide (5e):** ([***See spectra***](#g5))

**TLC**: R*_f_* = 1.00 (80:20 Petroleum ether: EtOAc).

**^1^H NMR** (400 MHz, CDCl_3_) δ 5.80 (m, 1H), 5.33 – 5.24 (m, 2H), 4.88 – 4.80 (m, 1H), 4.33 – 4.24 (m, 1H), 4.05 (br, 1H), 1.90 – 1.78 (m, 2H), 1.73 (m, 1H), 1.53 (dt, *J* = 14.4, 11.9 Hz, 1H), 1.39 (m, 1H), 0.94 (m, 6H) ppm.

**^13^C NMR** (101 MHz, CDCl_3_) δ 135.1, 117.4, 82.7, 56.4, 44.3, 35.6, 23.9, 23.0, 22.0 ppm.

**HRMS** (ESI) *m/z* calculated for C_9_H_17_NNaO_3_S [M+Na]^+^ 242.0896, found 242.0899.

**6-isobutyl-4-vinyl-1,2,3-oxathiazinane 2,2-dioxide (5e’):** ([***See spectra***](#gg5))

**TLC**: R*_f_* = 0.80 (80:20 Petroleum ether: EtOAc).

**^1^H NMR** (400 MHz, CDCl_3_) δ 5.79 (ddt, *J* = 16.9, 10.2, 6.7 Hz, 1H), 5.12 – 5.01 (m, 2H), 4.87 – 4.80 (m, 1H), 4.11 (br, 1H), 2.31 – 2.14 (m, 2H), 1.89 – 1.80 (m, 1H), 1.72 – 1.60 (m, 3H), 1.49 (s, 3H), 1.30 (s, 3H) ppm.

**^13^C NMR** (101 MHz, CDCl_3_) δ 136.8, 116.2, 80.6, 56.0, 41.6, 34.5, 32.1, 28.8, 25.3 ppm.

**HRMS** (ESI) *m/z* calculated for C_9_H_17_NNaO_3_S [M+Na]^+^ 242.0890, found 242.0890.

**6-(cyclobutylmethyl)-4,4-Dimethyl-1,2,3-oxathiazinane 2,2-dioxide (5f)** and **8-Isobutyl-7-oxa-6-thia-5-azaspiro[3.5]nonane 6,6-dioxide** (**5f’**): ([***See spectra***](#f5))

**TLC**: R*_f_* = 0.25 (80:20 Petroleum ether: EtOAc).

The title compound **5f** and **5f’** was synthesized according to [**GP-D**](#GPD) using 1-cyclobutyl-4-methylpentan-2-yl sulfamate **4f** (117 mg, 0.5 mmol, 1.0 equiv.), iodosylbenzene (440 mg, 2.0 mmol, 4.0 equiv.) and 9*H*-thioxanthen-9-one (5.3 mg, 0.025 mmol, 5 mol%) in dry CHCl_3_ (5.0 mL, 0.1 M). The product was isolated by silica gel column chromatography using a gradient of petroleum ether/ethyl acetate (9:1) and obtained as a mixture in a 1:0.8 ratio, with an overall yield of 59%, as a colourless oil.

**^1^H NMR** (400 MHz, CDCl_3_) δ 4.71 – 4.64 (m, 1H), 4.52 – 4.45 (m, 2H), 3.88 (s, 2H), 3.38 (td, *J* = 10.9, 3.4 Hz, 2H), 2.19 – 2.06 (m, 3H), 1.92 – 1.70 (m, 15H), 1.56 – 1.36 (m, 2H), 1.04 – 1.01 (m, 7H), 0.97 (d, *J* = 6.6 Hz, 3H), 0.90 (d, *J* = 7.1 Hz, 5H) ppm.

**^13^C NMR** (101 MHz, CDCl_3_) δ 93.2, 86.5, 72.5, 64.2, 47.8, 44.5, 38.7, 32.3, 30.0, 29.9, 28.0, 25.3, 24.5, 23.7, 23.6, 22.1, 19.3, 18.3, 15.8 ppm.

**HRMS** (ESI) *m/z* calculated for C_10_H_19_NNaO_3_S [M+Na]^+^ 256.1255, found 256.1256.

***Note****: The mixture of 2-regioisomers are inseparable by column chromatography.*

**6-(cyclohexylmethyl)-4,4-Dimethyl-1,2,3-oxathiazinane 2,2-dioxide** (**5g**): ([***See spectra***](#e5)) **and** **4-Isobutyl-3-oxa-2-thia-1-azaspiro[5.5]undecane 2,2-dioxide** (**5g’**): ([***See spectra***](#ee5))

**TLC**: R*_f_* = 0.25 (80:20 Petroleum ether: EtOAc).

The title compound **5e** and **5e’** was synthesized according to [**GP-D**](#GPD) using 1-cyclohexyl-4-methylpentan-2-yl sulfamate **4e** (131 mg, 0.5 mmol, 1.0 equiv.), iodosylbenzene (440 mg, 2.0 mmol, 4.0 equiv.) and 9*H*-thioxanthen-9-one (5.3 mg, 0.025 mmol, 5 mol%) in dry CHCl_3_ (5.0 mL, 0.1 M). The product was isolated by silica gel column chromatography using a gradient of petroleum ether/ethyl acetate (9:1) **5e** (69 mg, 0.26 mmol, 53%) as a colourless oil and **5e’** (29 mg, 0.11 mmol, 22%) as a colourless oil.

**^1^H NMR** (400 MHz, CDCl_3_) δ 4.93 (m, 1H), 3.98 (s, 1H), 1.81 (ddq, *J* = 14.5, 3.8, 1.8 Hz, 1H), 1.74 – 1.63 (m, 5H), 1.61 – 1.52 (m, 3H), 1.50 (s, 3H), 1.37 (ddd, *J* = 14.1, 8.3, 4.2 Hz, 1H), 1.29 (s, 3H), 1.28 – 1.12 (m, 3H), 0.99 – 0.85 (m, 2H) ppm.

**^13^C NMR** (101 MHz, CDCl_3_) δ 79.3, 56.0, 43.1, 42.2, 33.8, 33.2, 32.7, 32.2, 26.5, 26.3, 26.2, 25.3 ppm.

**HRMS** (ESI) *m/z* calculated for C_12_H_23_NNaO_3_S [M+Na]^+^ 284.1468, found 284.1470.

Spectral data were consistent with the literature.^[14]^

**^1^H NMR** (400 MHz, CDCl_3_) δ 4.96 – 4.84 (m, 1H), 3.84 (s, 1H), 2.46 (dd, *J* = 12.0, 3.0 Hz, 1H), 1.96 – 1.79 (m, 1H), 1.77 – 1.55 (m, 5H), 1.56 – 1.43 (m, 5H), 1.44 – 1.23 (m, 3H), 0.94 (dd, *J* = 7.9, 6.6 Hz, 6H) ppm.

**^13^C NMR** (101 MHz, CDCl_3_) δ 78.9, 58.1, 44.4, 41.8, 40.7, 33.0, 25.8, 23.9, 23.0, 22.0, 21.3, 21.0 ppm.

**HRMS** (ESI) *m/z* calculated for C_12_H_23_NNaO_3_S [M+Na]^+^ 284.1470, found 284.1470.

Spectral data were consistent with the literature.^[11]^

**4-cyclopropyl-4-methyl-1,2,3-oxathiazinane 2,2-dioxide (5h): (**[***See Spectra***](#cruded12spectra)**)**

**^1^H NMR** (400 MHz, CDCl_3_) δ 4.80 (td, *J* = 11.8, 2.6 Hz, 1H), 4.57 (ddd, *J* = 12.0, 4.6, 3.7 Hz, 1H), 4.21 – 4.09 (m, 1H), 1.80 – 1.72 (m, 1H), 1.47 – 1.42 (m, 1H), 1.39 (s, 3H), 1.17 (ddd, *J* = 8.5, 5.7, 3.0 Hz, 1H), 0.61 – 0.49 (m, 2H), 0.49 – 0.43 (m, 2H) ppm.

**^13^C NMR** (101 MHz, CDCl_3_) δ 68.8, 58.9, 32.0, 29.9, 22.6, 22.6, 1.1 ppm.

**HRMS** (ESI) *m/z* calculated for C_7_H_13_NNaO_3_S [M+Na]^+^ 191.0514, found 191.0519.

***Comment****: This result suggests that a freely diffusing radical at this position is unlikely and is consistent with either a concerted pathway or a short-lived/caged radical.*

# 7. Synthetic Transformations:

**Deprotection of 3b’ to afford cyclohexanamine (6):** ([***See spectra***](#seven))

A suspension of powdered Zn (53 mg, 0.8 mmol, 5.0 equiv) in 2 mL of a 1:1 MeOH/AcOH solution was treated with 2,2,2-trichloroethyl cyclohexylsulfamate (50 mg, 0.16 mmol). The reaction mixture was stirred vigorously at 40 °C for 16 hours. After cooling to room temperature (23 °C), the mixture was diluted with 1 mL of MeOH and filtered through a small pad of Celite. The reaction flask and Celite pad were rinsed with an additional 5 mL of MeOH. The combined filtrates were concentrated under reduced pressure to afford a white solid residue. Water (10 mL) was added to the residue, and the mixture was extracted with DCM (2 × 10 mL). The combined organic layers were dried over MgSO₄, filtered, and concentrated under reduced pressure to yield the desired product as a pale brick red oil (14.7 mg, 0.15 mmol, 92%), sufficiently pure.

**TLC**: R*_f_* = 0.25 (80:20 Petroleum ether: EtOAc).

**^1^H NMR** (400 MHz, CDCl_3_) δ 2.61 (tt, *J* = 10.6, 3.9 Hz, 1H), 1.85 – 1.76 (m, 2H), 1.70 (dt, *J* = 13.2, 3.5 Hz, 2H), 1.62 – 1.55 (m, 1H), 1.43 (s, 2H), 1.31 – 1.19 (m, 2H), 1.16 – 0.98 (m, 3H) ppm.

**^13^C NMR** (101 MHz, CDCl_3_) δ 50.6, 37.1, 25.8, 25.3 ppm.

**HRMS** *m/z* calculated for C_6_H_14_N [M+H]^+^ 100.1130, found 100.1119.

Spectral data were consistent with the literature.^[9]^

**Deprotection of 3m’ to afford Memantine** **hydrochloride (7): (**[***See spectra***](#six)**)**

To a solution of 2,2,2-trichloroethyl ((1*r*,3*R*,5*S*,7*r*)-3,5-dimethyladamantan-1-yl)sulfamate **3m’** (69 mg, 0.2 mmol, 1.00 eq.) in a mixture of acetonitrile-water (2:1, 3.0 mL) was added pyridine (0.32 mL, 4 mmol, 20.0 equiv.). The mixture was stirred at 100 °C for 24 h. H_2_O (5 mL) was then added to the mixture followed by 10.0 mL of EtOAc. The content was transferred to a separatory funnel. The organic layer was quenched with 5.0 mL of a solution of NaOH 1M then washed 5 times with water to remove the pyridine. Then HCl 1M (3 x 5 mL) was added and the aqueous phase was extracted from the organic phase containing the 2,2,2-trifluoroethan-1-ol. The aqueous phases were treated with (3 x 5 mL) of NaOH 1M and the organic phase was extracted with EtOAc then dried over MgSO_4_ and filtered. The filtrate was concentrated under reduced pressure, and subsequent addition of heptane and concentration was repeated until complete removal of the remaining pyridine. The desired product was obtained sufficiently pure as a white solid (28 mg, 0.13 mmol, 66%).

**^1^H NMR** (400 MHz, CDCl_3_) δ 2.24 – 2.18 (m, 1H), 1.91 – 1.82 (m, 2H), 1.68 (q, *J* = 11.8 Hz, 4H), 1.41 – 1.37 (m, 2H), 1.33 – 1.28 (m, 3H), 1.20 – 1.13 (m, 2H), 0.86 (s, 6H) ppm.

**^13^C NMR** (101 MHz, CDCl_3_) δ 54.4, 49.9, 46.6, 42.0, 39.4, 32.8, 29.9, 29.8 ppm.

**HRMS** *m/z* calculated for C_12_H_21_N [M]^+^ 179.3408, found 179.3420.

Spectral data were consistent with the literature.^[10]^

**Nucleophilic addition of 3t’ (8, Azidation):** ([***See spectra***](#eight))

To a 25 mL round bottom flask equipped with a Teflon-coated magnetic stir bar was added 2,2,2-trichloroethyl ((1R,2R)-2-iodocyclohexyl)sulfamate **3t’** (100 mg, 0.23 mmol, 1.0 equiv.) in dry DMF (2 mL) followed by sodium azide (75 mg, 1.15 mmol, 5.0 equiv.). The resulting mixture was degassed *via* ‘freeze-pump-thaw’ procedure (3 times) then stirred at room temperature for 16 h. Upon completion of the reaction, the crude product was washed with sodium thiosulfate solution (2 x 5 mL) and the organic layer was dried over MgSO_4_. The solvent was removed by vacuum and the crude product was purified by column chromatography on silica gel silica: 60; eluant: petroleum ether/ethyl acetate (9:1) to provide pure product as a brown solid (58 mg, 0.16 mmol, 72%).

**TLC**: R*_f_* = 0.10 (80:20 Petroleum ether: EtOAc).

**^1^H NMR** (400 MHz, CDCl_3_) δ 4.90 (d, *J* = 4.7 Hz, 1H), 4.69 (q, *J* = 10.7 Hz, 2H), 3.32 – 3.13 (m, 2H), 2.36 (m, 1H), 2.21 – 2.13 (m, 1H), 1.87 – 1.73 (m, 2H), 1.52 – 1.45 (m, 1H), 1.37 – 1.29 (m, 3H) ppm.

**^13^C NMR** (101 MHz, CDCl_3_) δ 93.5, 78.5, 63.4, 58.4, 32.2, 30.5, 24.1, 23.9 ppm.

**HRMS** (ESI) *m/z* calculated for C_8_H_12_Cl_3_N_4_O_3_S [M-H]^-^ 348.9695, found 348.9701.

**An E2-type *β*-elimination of 3t (9):** ([***See spectra***](#nine))

To a 25 mL round bottom flask equipped with a Teflon-coated magnetic stir bar was added 2,2,2-trifluoroethyl ((1R,2R)-2-iodocyclohexyl)sulfamate **3t** (100 mg, 0.26 mmol, 1.0 equiv.) in CH_3_CN (3 mL). The resulting mixture was degassed *via* ‘freeze-pump-thaw’ procedure (3 times) then stirred at 100^o^C for 16 h. Upon completion of the reaction, the crude product was washed with sodium thiosulfate solution (2 x 5 mL) and the organic layer was dried over MgSO_4_. The solvent was removed by vacuum and the crude product was purified by column chromatography on silica gel silica: 60; eluant: petroleum ether/ethyl acetate (10:1) to provide pure product as a pale-yellow solid (65 mg, 0.25 mmol, 96%).

**TLC**: R*_f_* = 0.50 (90:10 Petroleum ether: EtOAc).

**^1^H NMR** (400 MHz, CDCl_3_) δ 5.94 (m, 1H), 5.71 – 5.64 (m, 1H), 4.60 (d, *J* = 8.6 Hz, 1H), 4.41 (q, *J* = 7.9 Hz, 2H), 4.04 (m, 1H), 2.03 – 1.97 (m, 2H), 1.78 – 1.62 (m, 4H) ppm.

**^13^C NMR** (101 MHz, CDCl_3_) δ 133.0, 126.0, 122.3 (q, *J* = 277.6 Hz), 65.2 (q, *J* = 37.7 Hz), 50.6, 24.7, 19.2 ppm.

**^19^F NMR** (376 MHz, CDCl_3_) δ -73.80 (t, *J* = 8.0 Hz) ppm.

**HRMS** (ESI) *m/z* calculated for C_8_H_12_F_3_NO_3_S [M]^+^ 259.0155, found 259.0210.

Spectral data were consistent with the literature.^[11]^

**1,5-HAT cyclization of 3c’ (10):** ([***See spectra***](#ten))

To a 25 mL round bottom flask equipped with a Teflon-coated magnetic stir bar was added 2,2,2-trifluoroethyl (2-iodocyclohexyl) sulfamate **3c’** (162 mg, 0.5 mmol, 1.0 equiv.) and 1,3-diiodo-5,5- dimethylhydantoin (760 mg, 2.0 mmol, 4.0 equiv.) in CH_3_CN (5 mL). The resulting mixture was degassed *via* ‘freeze-pump-thaw’ procedure (3 times) then stirred at 80^o^C for 24 h. Upon completion of the reaction, the crude product was washed with sodium thiosulfate solution (2 x 10 mL) and the organic layer was dried over MgSO_4_. The solvent was removed by vacuum and the crude product was purified by column chromatography on silica gel silica: 60; eluant: petroleum ether/ethyl acetate (8:2) to provide pure product as a colorless oil (71 mg, 0.26 mmol, 52%).

**TLC**: R*_f_* = 0.25 (90:10 Petroleum ether: EtOAc).

**^1^H NMR** (400 MHz, CDCl_3_) δ 4.69 – 4.58 (m, 2H), 4.52 – 4.47 (m, 1H), 4.36 – 4.29 (m, 1H), 2.53 – 2.38 (m, 5H), 1.94 – 1.83 (m, 3H), 0.90 – 0.82 (m, 2H) ppm.

**^13^C NMR** (101 MHz, CDCl_3_) δ 93.5, 78.2, 67.2, 58.3, 32.2, 30.2, 29.9, 28.1, 27.9 ppm.

**HRMS** (ESI) *m/z* calculated for C_9_H_14_Cl_3_NO_3_S [M+H]^+^ 321.0461, found 321.0457.

# 8. X-ray analysis:

**Table S7**. Crystal data and structure refinement for **3t’**.

A suitable crystal of **3t’** was prepared from CHCl_3_ by slow evaporation of the solvent.

**CCDC Number**: 2190559

**Bond precision**: C-C = 0.0104 A Wavelength=1.54178

**Cell**: a=9.467(2) b=21.885(4) c=7.419(1)

alpha=90 beta=100.759(11) gamma=90

**Temperature**: 293 K

**Calculated** **Reported**

**Volume** 1510.1(5) 1510.1(5)

**Space** **group** P 21/c P 21/c

**Hall** **group** -P 2ybc -P 2ybc

**Moiety formula** C_8_ H_13_ Cl_3_ I N O_3_ S C_8_ H_13_ Cl_3_ I N O_3_ S

**Sum formula** C_8_ H_13_ Cl_3_ I N O_3_ S C_8_ H_13_ Cl_3_ I N O_3_ S

**Mr** 436.50 436.50

**Dx,g cm-3** 1.920 1.920

**Z** 4 4

**Mu (mm-1)** 22.834 22.834

**F000** 848.0 848.0

**F000’** 854.52

**h,k,lmax** 11,27,9 11,27,9

**Nref** 2983 2930

**Tmin,Tmax** 0.014,0.023 0.283,0.754

**Tmin’**  0.001

Correction method= # Reported T Limits: Tmin=0.283 Tmax=0.754

AbsCorr = MULTI-SCAN

Data completeness= 0.982 Theta(max)= 72.126

R(reflections)= 0.0704( 2743)

wR2(reflections)= 0.1943( 2930)

S = 1.060 Npar= 182

**
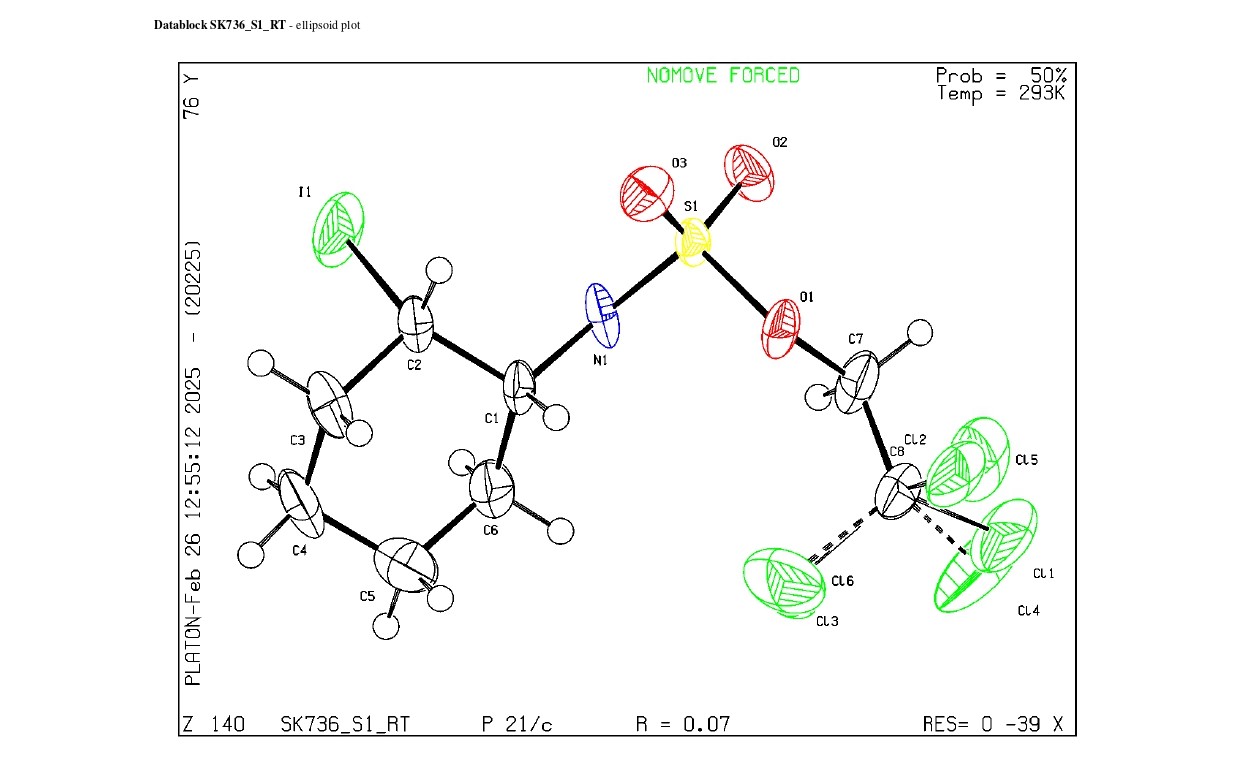
**

**Figure S3**: Single-crystal X-ray crystal structure of compound **3t’**

# 9. Mechanistic investigations:

## 9.1 UV/Visible absorption spectroscopy:

To determine the absorption spectra of **thioxanthone**, of various components, compounds **2a** and **PhIO**, were dissolved in dry CHCl_3_ (0.1 mM). The corresponding sample solutions were filled in a quartz cuvette (10 mm width) and the spectra were obtained using a Jasco spectrophotometer V-770.

**Figure S4**: UV/Vis absorption spectrum of **Thioxanthone**, **2a** and **PhIO** in CHCl_3_.

## 9.2 Stern-Volmer luminescence quenching analysis:

The Stern-Volmer luminescence quenching study has been performed using a Jasco spectrofluorometer FP-8500. The corresponding fluorescence emission spectra for thioxanthone were measured employing the following parameters: excitation wavelength: 370 nm, excitation bandwidth = 5 nm, data interval = 1 nm, scan speed = 1000 nm/min, response time = 0.1 s. Sample solutions were filled in a quartz cuvette with a width of 10 mm.

For the quenching studies, a stock solution of thioxanthone in a solvent mixture of CHCl_3_/MeOH (2:1, 0.05 M) has been prepared. The stock solution has been used to dissolve increasing amounts of the quencher, as described in more detail in the following tables. The Stern-Volmer plot was obtained by plotting the *I_0_*/*I* ration against the quencher concentration after determining the intensity of the fluorescence emission spectrum of thioxanthone at 429 nm.

***Note***: Due to low solubility of compound **2a** and **PhIO**, a solvent mixture of CHCl_3_/MeOH (2:1) instead of CHCl_3_ has been used instead.

**Table S8**: Luminescence quenching data for **2a** (**Iminoiodinane**) in CHCl_3_/MeOH (2:1)

| [**2a**] (mM) | 0 | 2.5 | 5 | 10 | 20 | 50 |
| --- | --- | --- | --- | --- | --- | --- |
| *I* (a.u.) | 9232 | 9058 | 8863 | 8800 | 7940 | 5333 |
| *I_0_*/*I* | 1 | 1.01 | 1.04 | 1.04 | 1.16 | 1.73 |

**Table S9**: Luminescence quenching data for **PhIO** in CHCl_3_/MeOH (2:1)

| [**PhIO**] (mM) | 0 | 2.5 | 5 | 10 | 20 | 50 |
| --- | --- | --- | --- | --- | --- | --- |
| *I* (a.u.) | 9232 | 9164 | 8888 | 8319 | 8024 | 7507 |
| *I_0_*/*I* | 1 | 1.01 | 1.03 | 1.11 | 1.15 | 1.23 |

**Table S10**: Luminescence quenching data for **1b** (**Cyclohexane**) in CHCl_3_/MeOH (2:1)

| [**1b**] (mM) | 0 | 2.5 | 5 | 10 | 20 | 50 |
| --- | --- | --- | --- | --- | --- | --- |
| *I* (a.u.) | 9232 | 9396 | 9347 | 9247 | 9205 | 8811 |
| *I_0_*/*I* | 1 | 0.98 | 0.99 | 1.00 | 1.00 | 1.05 |

[**2a**] = 0 mM

[**2a**] = 2.5 mM

[**2a**] = 5 mM

[**2a**] = 10 mM

[**2a**] = 20 mM

[**2a**] = 50 mM

**Figure S5**: Fluorescence emission spectra for thioxanthone with increasing concentrations of compound **2a**.

[**PhIO**] = 0 mM

[**PhIO**] = 2.5 mM

[**PhIO**] = 5 mM

[**PhIO**] = 10 mM

[**PhIO**] = 20 mM

[**PhIO**] = 50 mM

**Figure S6**: Fluorescence emission spectra for thioxanthone with increasing concentrations of compound **PhIO**.

**Figure S7**: Fluorescence emission spectra for thioxanthone with increasing concentrations of compound **cyclohexane 1b**.

**Figure S8**: Stern-Volmer plot for thioxanthone with compounds **2a (**Blue line**)**; **PhIO** (Green line); and cyclohexane **1b** (Orange line).

For each of these plots, the slope corresponds to the Stern–Volmer constant (K_SV_), as defined in Equation 1. Comparison of these constants indicates that energy transfer occurs between the excited photocatalyst and iminoiodinane **2a**.^[16]^

I^0^ /I = 1 + K_SV_[Q] [Eq. 1]

***Comment:*** *Stern–Volmer quenching experiments revealed that only iminoiodinane* ***2a*** *efficiently quenches the excited state of thioxanthone, while iodosylbenzene showed minimal effect and cyclohexane showed none. These results suggest that* ***2a*** *directly interacts with the excited photocatalyst, initiating the photochemical process.*

## 9.4 Table S11 Comparison of various triplet photocatalysts.

| Entry | PC (1 mol%) | *E*_1/2_(PC^+^/PC^*^) | *E*_1/2_(PC^*^/PC^-^) | *E*_T_(kcal/mol) | Yield of 3b |
| --- | --- | --- | --- | --- | --- |
| 1 | --- | --- | --- | --- | --- |
| 2 | [Mes-Acr](ClO_4_) | +2.81 V | --- | 44.7 | 10% |
| 3 | Eosin Y | +1.18 V | -1.60 V | 45.4 | 8% |
| 4 | Ru(bpy)_3_Cl_2_ | +0.77 V | -0.81 V | 46 | ~10% |
| 5 | *fac*-Ir(ppy)_3_ | +0.31 V | -1.73 V | 58.1 | <5% |
| 6 | 4-CzIPN | +1.43 V | -1.18 V | 61.6 | ~10% |
| 7 | Ir[dFFppy]_2_(4,4′-dCF_3_bpy)PF_6_ | +1.21 V | -0.89 V | 61.8 | <5% |
| 8 | TBADT | +2.44 V | -1.40 V | 61.8 | 40% |
| 9 | TXO (5 mol%) | +1.18 V | -1.11 V | 65.5 | 58% |
|  |  |  |  |  |  |

To a 10 mL Schlenk tube equipped with a Teflon coated magnetic stir bar was 2,2,2-trifluoroethyl (phenyl-λ^3^-iodaneylidene) sulfamate **2a** (53 mg, 0.14 mmol, 1.0 equiv.), Iodosyl benzene (77 mg, 0.35 mmol, 2.5 equiv.) and photocatalyst (5 mol%) in dry CHCl_3_ (2.0 mL) were added. The Schlenk tube was evacuated and backfilled with nitrogen three times. Subsequently, cyclohexane **1b** (117 mg (153 µL), 1.4 mmol, 10.0 equiv.) was added against the positive flow of nitrogen. The reaction mixture was then stirred under irradiation with LEDs (2 x 40 W, λ_max_ = 370 nm). Later, the solvent was removed in vacuo. Mesitylene (19 µL, 0.14 mmol) was added as internal standard, and the yield was determined by crude ^1^H-NMR analysis.

9.5 Radical trapping experiments**:**

**Conditions**: **1b** Cyclohexane (1.4 mmol, 10 equiv.), **2a** Iminoiodinane (0.14 mmol), Iodosylbenzene (0.35 mmol, 2.5 equiv.), Radical trapping reagent (X equiv.) and TXO (1.5 mg, 0.007 mmol, 5.0 mol%) in dry CHCl_3_ (0.1 M), irradiation with LEDs (λ_max_ = 370 nm, 2 x 40W at 100% intensity) under nitrogen atmosphere at room temperature for 16 h.

**
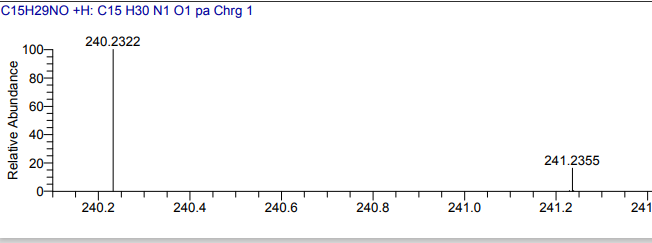
**

**Figure S9**: HRMS analysis of the reaction with TEMPO.

## **9.6 Radical quenching experiments:[**^17]^

***Comment****: These results indicate that the* ***triplet nitrene species*** *generated via energy transfer is effectively quenched by* ***2,5-dimethylhexa-2,4-diene****, consistent with its role as a triplet-state quencher and supporting the involvement of a triplet nitrene pathway.*

## 9.7 Light on/off experiments:

To an 50 mL round bottom flask equipped with a Teflon coated magnetic stir bar was 2,2,2-trifluoroethyl (phenyl-λ^3^-iodaneylidene) sulfamate **2a** (381 mg, 1.0 mmol, 1.0 equiv.), Iodosyl benzene (550 mg, 2.5 mmol, 2.5 equiv.) and photocatalyst (11.0 mg, 0.05 mmol, 5 mol%) in dry CDCl_3_ (10.0 mL, 0.1 M) were added. The round bottom flask was evacuated and backfilled with nitrogen three times. Subsequently, cyclohexane **1b** (841 mg (1.1 mL), 10.0 mmol, 10.0 equiv.) was added against the positive flow of nitrogen. The reaction mixture was then stirred under irradiation with LEDs (40 W, λ_max_ = 370 nm). Later, 0.5 mL of aliquot was taken after every 1 h and used to determine the conversion by crude ^1^H-NMR using mesitylene as internal standard. After a total of 10 h, the determined yields were plotted against the reaction time.

**Figure S10**: Light on/off experiments.

## 9.8 Study of the reaction with deuterated substrate:

To a 10 mL Schlenk tube equipped with a Teflon coated magnetic stir bar was 2,2,2-trichloroethyl (phenyl-λ^3^-iodaneylidene) sulfamate **2b** (76 mg, 0.2 mmol, 1.0 equiv.), Iodosyl benzene (110 mg, 0.5 mmol, 2.5 equiv.) and TXO (2.1 mg, 0.01 mmol, 5 mol%) in dry CHCl_3_ (2.0 mL, 0.1 M) were added. The Schlenk tube was evacuated and backfilled with nitrogen three times. Subsequently, cyclohexane-*d_12_* (192 mg (215 µL), 2.0 mmol, 10.0 equiv.) was added against the positive flow of nitrogen. The reaction mixture was then stirred under irradiation with LEDs (2 x 40 W, λ_max_ = 370 nm) at room temperature (35 °C to 38 °C) for 16 h. Upon completion of the reaction, the crude product was washed with sodium thiosulfate solution (2 x 5 mL) and the organic layer was dried over MgSO_4_. The solvent was removed by vacuum and the crude product was purified by column chromatography on silica gel silica: 60; eluant: petroleum ether/ethyl acetate (10:1) (**3b’-*d_11_***, 17 mg, 32%) as a white solid.

**^1^H NMR** (400 MHz, CDCl_3_) δ 4.62 (s, 2H), 4.51 (s, 1H) ppm.

**^13^C NMR** (101 MHz, CDCl_3_) δ 93.7, 78.3 ppm.

***Note*:** *We have observed 39% of N-Deuteration from the crude ^1^H-NMR mixture* [***(See spectra)***](#cruded12spectra) *and 24% of N-Deuteration after the purification.*

***Note****: In the ^13^C-NMR spectrum of* ***2,2,2-trichloroethyl deuterium(cyclohexyl-d₁₁)sulfamate****, only the trichloroethyl CH₂ carbon and carbon from CCl_3_ signals were observed. The absence of signals from the cyclohexyl ring is attributed to deuterium substitution, which leads to loss of Nuclear Overhauser Enhancement (NOE) and signal broadening due to ^13^C–²H coupling, resulting in significantly reduced signal intensity.*

Crude mixture ^1^H NMR Spectrum of **Deuteration experiment 3b’- *d_12_*** (400 MHz, CDCl_3_)

^1^H NMR Spectrum of **3b’- *d_12_*** (400 MHz, CDCl_3_)

^13^C NMR Spectrum of **3b’-*d_12_*** (101 MHz, CDCl_3_)

## 9.9 Kinetic Isotopic effects: KIE is determined from two parallel reactions:

To two separate 10 mL Schlenk tubes were equipped with Teflon-coated magnetic stir bars were added 2,2,2-trichloroethyl (phenyl-λ³-iodaneylidene) sulfamate **2b** (431 mg, 1.0 mmol, 1.0 equiv.), iodosylbenzene (550 mg, 2.5 mmol, 2.5 equiv.), and photocatalyst (10.6 mg, 0.05 mmol, 5 mol%) in dry CDCl₃ (10.0 mL, 0.1 M). Each Schlenk tube was evacuated and backfilled with nitrogen three times. Subsequently, **cyclohexane 1b** (841 mg (1.1 mL), 10.0 mmol, 10.0 equiv.) was added to one flask and **cyclohexane-*d₁₂* 1b′** (962 mg, (1.08 mL), 10.0 mmol, 10.0 equiv.) was added to the other under a positive flow of nitrogen. The reaction mixtures were then stirred separately under irradiation with LEDs (2 × 40 W, λ_max_ = 370 nm) at room temperature (35–38 °C). Later, 0.5 mL of aliquot was taken after every 30 minutes and used to determine the conversion by crude ^1^H-NMR using mesitylene as internal standard. After a total of 5 h, the determined yields were plotted against the reaction time.

| S.No | Time (Min) | 3b‘ (%) | 3b‘-*d_11_* (%) | P_H_/P_D_ |
| --- | --- | --- | --- | --- |
| 1 | 30 | 5 | 0 | 0 |
| 2 | 60 | 7 | 1.5 | 4.60 |
| 3 | 90 | 14 | 3 | 4.60 |
| 4 | 120 | 16 | 4 | 4.00 |
| 5 | 150 | 19 | 5 | 3.80 |
| 6 | 180 | 21 | 5.5 | 3.81 |
| 7 | 210 | 24 | 7 | 3.42 |
| 8 | 240 | 26 | 9 | 2.88 |
| 9 | 270 | 29 | 10.5 | 2.76 |
| 10 | 300 | 30 | 11 | 2.72 |


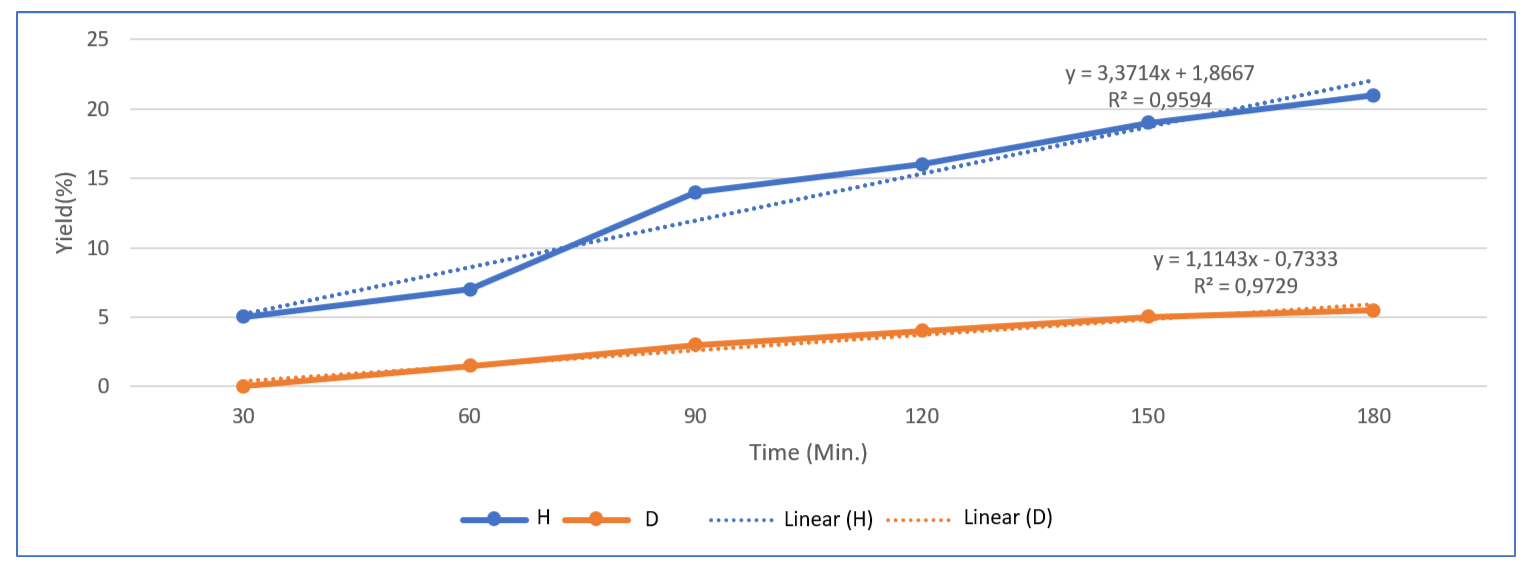


**Figure S11**: Initial rates of cyclohexane (blue line) and cyclohexane-*d_11_* (orange line).

$$KIE=\frac{k_{H}}{k_{D}}= \frac{3.3714}{1.1143}= \approx3.03$$

***Comment****: After 30 min, the reaction became homogeneous; conversion rose from 7% to 14% (60–90 min) and then progressed slowly (~2-4% per 30 min).*

## 9.9.1 Radical clock experiment: ([*See spectra*](#six))

To a 25 mL round bottom flask equipped with a Teflon-coated magnetic stir bar was added 3-cyclopropylbutyl sulfamate (48 mg, 0.25 mmol, 1.0 equiv.), iodosylbenzene (220 mg, 1.0 mmol, 4.0 equiv.) and 9*H*-thioxanthen-9-one (3.7 mg, 0.013 mmol, 5 mol%) in dry CHCl_3_ (2.5 mL, 0.1 M). The resulting mixture was degassed *via* ‘freeze-pump-thaw’ procedure (3 times). The reaction mixture was then stirred for 15 minutes at room temperature then irradiate with LEDs (2 x 40 W, λ_max_ = 370 nm) at room temperature (35 °C to 38 °C) for 16 h. Upon completion of the reaction, the crude product was washed with sodium thiosulfate solution (2 x 10 mL) and the organic layer was dried over MgSO_4_. The solvent was removed by vacuum, and the crude product was purified by column chromatography on silica gel: silica 60; eluant: petroleum ether/ethyl acetate (10:1 to 8:2) afforded **5h** in 28% yield (13 mg) as colorless oil.

***Comment****: This result suggests that the formation of a freely diffusing radical is unlikely and is instead consistent with either a concerted mechanism or a short-lived, caged radical intermediate.*

**4-cyclopropyl-4-methyl-1,2,3-oxathiazinane 2,2-dioxide (5h): (**[***See Spectra***](#cruded12spectra)**)**

**^1^H NMR** (400 MHz, CDCl_3_) δ 4.80 (td, *J* = 11.8, 2.6 Hz, 1H), 4.57 (ddd, *J* = 12.0, 4.6, 3.7 Hz, 1H), 4.21 – 4.09 (m, 1H), 1.80 – 1.72 (m, 1H), 1.47 – 1.42 (m, 1H), 1.39 (s, 3H), 1.17 (ddd, *J* = 8.5, 5.7, 3.0 Hz, 1H), 0.61 – 0.49 (m, 2H), 0.49 – 0.43 (m, 2H) ppm.

**^13^C NMR** (101 MHz, CDCl_3_) δ 68.8, 58.9, 32.0, 29.9, 22.6, 22.6, 1.1 ppm.

**HRMS** (ESI) *m/z* calculated for C_7_H_13_NNaO_3_S [M+Na]^+^ 191.0514, found 191.0519.

***Comment****: This result suggests that a freely diffusing radical at this position is unlikely and is consistent with either a concerted pathway or a short-lived/caged radical.*

# 10. Computational studies

## 10.1. Computational details:

DFT computational studies were carried out using Gaussian16 program package.^[18]^ All the structures were optimized using the ωB97XD^[19]^ functional and Def2SVP^[20]^ as the basis set. Frequency calculations were calculated at standard conditions, and all the stationary points were characterized as minima (0 imaginary frequencies) or transition states (1 imaginary frequency). Relaxation to reactants and products and IRC calculations were performed when required to further characterize the PES. In addition, single point energy calculations were recalculated with the Def2TZVPP basis set to further refine the potential energies. Solvation was included using the SMD implicit solvent model^[21]^ (chloroform as the solvent) in both optimizations/frequencies and single point calculations. Free energies (G) were calculated by the sum of E(Def2TZVPP) and the G correction (Def2SVP). The standard state was corrected from 1atm to 1M by adding 1.89 kcal/mol when needed. All the 3D structures were drawn using CYLview 1.0 software. ^[22]^

**Calculation of Single Electron Transfer (SET) steps by Marcus Theory**

To gain more insights into SET processes, we estimated the barrier of SET using the Marcus theory,^[23]^ which can be calculated using the following formulas, as previously reported by Maseras^[24]^ :

$$\Delta G^{\ddagger}=\frac{{(\lambda+\Delta G^{0})}^{2}}{4\lambda}$$

Where $\Delta G^{0}$ is the standard free energy of the reaction step (difference between reactants and products of the SET step), and $\lambda$ is the reorganization energy of all nuclei and solvent molecules involved in the step. The reorganization term $\lambda$ is computed as the sum of the nuclear reorganization $\lambda$_N_ and the solvent reorganization $\lambda$_S_.

$$\lambda= \lambda_{N}+\lambda_{S}$$

The nuclear reorganization $\lambda$_N_ is obtained by calculating the gas phase energy difference between the Donor (D) and Acceptor (A) species involved in the SET. Numbers 1 and 2 are referred to the initial and final structure, and α and β to the electronic state of each molecule:

$$\lambda_{N}=D2\alpha+A2\beta-D1\alpha-A1\beta$$

The solvent reorganization $\lambda$_S_ is calculated using the continuum solvent model, maintaining the same geometry for D1 and A1 and by comparing the energy of the solvation cage between the initial and final electronic states (δ - ε and $\alpha$ – β respectively):

$$\lambda_{S}=D1\delta+A1\varepsilon-D1\alpha-A1\beta$$

**Table S12:** Calculated free energy barriers (kcal/mol) of SET step.

|  | $\boldsymbol{\lambda}_{\boldsymbol{N}}$ | $\boldsymbol{\lambda}_{\boldsymbol{S}}$ | $\boldsymbol{\lambda}$ | $\boldsymbol{\Delta}\boldsymbol{G}^{\boldsymbol{0}}$ | $\boldsymbol{\Delta}\boldsymbol{G}^{\boldsymbol{\ddagger}}$ |
| --- | --- | --- | --- | --- | --- |
| **SET 1a’** | 61.592 | 4.379 | 65.970 | -15.731 | 9.565 |

**Calculation of Energy Transfer (ET) steps by asymmetric Marcus Theory**

The barrier of ET can be estimated by applying the asymmetric version of the Marcus Theory as recently reported by Maseras,^[25]^ using the following formulas:

$$\Delta G^{\ddagger}=\lambda_{R}\left( \frac{-\lambda_{P}+ \sqrt{\lambda_{R}\lambda_{P}+(\lambda_{R}-\lambda_{P})\Delta G^{0}}}{\lambda_{R}-\lambda_{P}} \right)^{2}$$

Where $\Delta G^{0}$ is the standard free energy of the reaction step (difference between reactants and products of the SET step).

$\lambda_{P}$ and $\lambda_{R}$ are defined as the reorganization energies and can be obtained using the following formulas:

$$\lambda_{R}= V_{A*}\left( q_{A} \right)-V_{A*}\left( q_{A*} \right)+V_{B}\left( q_{B*} \right)-V_{B}\left( q_{B} \right)$$

$$\lambda_{P}= V_{A}\left( q_{A*} \right)-V_{A}\left( q_{A} \right)+V_{B*}\left( q_{B} \right)-V_{B*}\left( q_{B*} \right)$$

Where V corresponds to the potential electronic energy at the equilibrium nuclear (A*, B, A and B*) and solvent configurations of their counterparts (A, B*, A* and B).

**Table S14:** Calculated free energy barriers (kcal/mol) of ET steps.

|  | $\boldsymbol{\lambda}_{\boldsymbol{P}}$ | $\boldsymbol{\lambda}_{\boldsymbol{R}}$ | $\boldsymbol{\Delta}\boldsymbol{G}^{\boldsymbol{0}}$ | $\boldsymbol{\Delta}\boldsymbol{G}^{\boldsymbol{\ddagger}}$ |
| --- | --- | --- | --- | --- |
| **ET1a’** | 73.061 | 57.049 | -41.968 | 2.711 |
| **ET4a’** | 50.943 | 27.413 | -46.313 | 0.06 |

## 10.2 Free energy profile for the intermolecular amination of 1a’:

**Figure S12**: Free energy profile of the intermolecular reaction mechanism at the ωB97xD/Def2TZVPP//ωB97xD/Def2SVP level of theory. Energies in kcal/mol.

## 10.3. Hydrogen atom transfer step for toluene:

**Figure S13**: ΔG^‡^ values for the HAT step with toluene at the ωB97xD/Def2TZVPP//ωB97xD/Def2SVP level of theory. Energies in kcal/mol.

## 10.4. Free energy profile for the intramolecular amination of 4a’:

**Figure S14**: Free energy profile of the intramolecular reaction mechanism at the ωB97xD/Def2TZVPP//ωB97xD/Def2SVP level of theory. Energies in kcal/mol.

## 10.5. Regioselectivity studies

**Figure S15**: Free energy profile of the HAT step for cis- and trans-1,4-dimethylcyclohexane at the ωB97xD/Def2TZVPP//ωB97xD/Def2SVP level of theory. Energies in kcal/mol.

**Figure S16**: ΔG^‡^ values for the HAT step with norbornane, adamantane and chloroadamantane at the ωB97xD/Def2TZVPP//ωB97xD/Def2SVP level of theory. Energies in kcal/mol.

# 11. XYZ Coordinates

|  | **Cyclohexane**  E (Def2TZVPP): -235.9048056 A.U.  G_corr_ (Def2SVP): 0.142255 A.U.  6 0.007113000 -1.508631000 -0.198517000  6 1.267755000 -0.770385000 0.256697000  6 1.269853000 0.683211000 -0.221271000  6 0.000884000 1.418773000 0.214700000  6 -1.259718000 0.680597000 -0.240683000  6 -1.261902000 -0.773007000 0.237262000  1 2.163792000 1.207708000 0.153438000  1 1.317185000 -0.787414000 1.360691000  1 2.168396000 -1.292996000 -0.104356000  1 0.005177000 -2.539102000 0.192343000  1 -0.007650000 1.501643000 1.316783000  1 0.002820000 2.449324000 -0.175948000  1 -2.160375000 1.203224000 0.120306000  1 -1.309028000 0.697666000 -1.344681000  1 -1.328249000 -0.790169000 1.340361000  1 -2.155813000 -1.297463000 -0.137575000  1 1.336047000 0.700375000 -1.324383000  1 0.015663000 -1.591749000 -1.300578000 |
| --- | --- |
|  | **3b‘**  E (Def2TZVPP): -2372.605229 A.U.  G_corr_ (Def2SVP): 0.098649 A.U.  16 1.084387000 -0.587563000 -0.569056000  8 0.199231000 -0.897741000 -1.664952000  8 1.287784000 -1.505825000 0.531316000  7 2.529329000 -0.157719000 -1.153876000  8 0.530738000 0.818247000 0.089087000  6 0.000275000 0.867540000 1.388291000  1 0.393014000 1.773072000 1.873003000  1 0.291044000 -0.014295000 1.978567000  6 -1.529785000 0.965829000 1.369754000  17 -2.250225000 -0.511580000 0.683921000  17 -2.050170000 2.375683000 0.409867000  17 -2.069345000 1.162051000 3.062324000  1 3.208901000 -0.014652000 -0.410124000  6 2.093784000 1.748591000 -5.037446000  6 2.262426000 2.633093000 -3.802209000  6 1.922941000 1.876322000 -2.517542000  6 2.747947000 0.590170000 -2.407162000  6 2.582699000 -0.300348000 -3.641051000  6 2.922456000 0.469511000 -4.917597000  1 2.377107000 2.304120000 -5.945462000  1 1.028118000 1.481102000 -5.148818000  1 1.629630000 3.530829000 -3.880154000  1 3.307106000 2.988994000 -3.749910000  1 2.102048000 2.508043000 -1.633694000  1 0.850519000 1.619086000 -2.514770000  1 3.810555000 0.876536000 -2.343400000  1 3.225717000 -1.187832000 -3.534310000  1 1.542375000 -0.657092000 -3.687953000  1 2.761565000 -0.177662000 -5.793830000  1 3.996022000 0.731044000 -4.913533000 |
|  | **TXO**  E (Def2TZVPP): -973.6664128 A.U.  G_corr_ (Def2SVP): 0.13669 A.U.  6 -9.136694000 -0.380628000 0.007948000  6 -7.753329000 -0.456777000 0.020269000  6 -6.980386000 0.717382000 0.015310000  6 -7.609645000 1.973198000 -0.002021000  6 -9.016150000 2.021196000 -0.014266000  6 -9.776691000 0.865444000 -0.009477000  6 -6.882082000 3.271335000 -0.008346000  6 -5.393895000 3.274703000 0.003538000  6 -4.602713000 2.113977000 0.020773000  6 -3.200787000 2.218704000 0.030530000  1 -2.588720000 1.313288000 0.043561000  6 -2.594831000 3.464752000 0.023416000  6 -3.372318000 4.629925000 0.006476000  6 -4.751952000 4.527087000 -0.003291000  1 -9.725133000 -1.300812000 0.011852000  1 -7.258633000 -1.431177000 0.033717000  1 -9.486359000 3.005789000 -0.027660000  1 -10.866742000 0.924168000 -0.019207000  1 -1.504641000 3.531408000 0.031130000  1 -2.893762000 5.611074000 0.000975000  1 -5.383936000 5.416519000 -0.016522000  16 -5.244223000 0.483674000 0.031629000  8 -7.499577000 4.322771000 -0.022735000 |
|  | **TXO^3^**  E (Def2TZVPP): -973.5570718 A.U.  G_corr_ (Def2SVP): 0.130159 A.U.  6 -9.127873000 -0.406662000 0.008236000  6 -7.743396000 -0.463203000 0.020604000  6 -6.993365000 0.724940000 0.015420000  6 -7.609167000 2.015688000 -0.002174000  6 -9.021822000 2.027437000 -0.014415000  6 -9.755767000 0.851848000 -0.009338000  6 -6.880598000 3.268820000 -0.007907000  6 -5.431293000 3.295179000 0.003265000  6 -4.603017000 2.129305000 0.020700000  6 -3.200062000 2.206975000 0.030488000  1 -2.606470000 1.288920000 0.043282000  6 -2.576328000 3.444392000 0.023566000  6 -3.370594000 4.605070000 0.006759000  6 -4.754605000 4.535270000 -0.003194000  1 -9.717197000 -1.324555000 0.012002000  1 -7.229220000 -1.427860000 0.034122000  1 -9.514204000 3.000001000 -0.027991000  1 -10.847252000 0.906348000 -0.019255000  1 -1.487722000 3.512998000 0.031065000  1 -2.887636000 5.585414000 0.001331000  1 -5.365415000 5.438136000 -0.016210000  16 -5.271058000 0.529719000 0.031124000  8 -7.529136000 4.372921000 -0.023977000 |
|  | **TXO^+^**  E (Def2TZVPP): -973.4330594A.U.  G_corr_ (Def2SVP): 0.135395 A.U.  6 -9.112688000 -0.401396000 0.005826000  6 -7.727703000 -0.455194000 0.025992000  6 -6.988073000 0.747565000 0.021883000  6 -7.635336000 2.006963000 0.001227000  6 -9.024975000 2.026318000 -0.018991000  6 -9.760742000 0.835834000 -0.017162000  6 -6.906051000 3.311623000 0.000330000  6 -5.411293000 3.314094000 0.009064000  6 -4.625657000 2.136189000 0.024637000  6 -3.215047000 2.197402000 0.028495000  1 -2.625861000 1.277240000 0.039169000  6 -2.588028000 3.433787000 0.017186000  6 -3.353890000 4.601884000 0.003472000  6 -4.751984000 4.537594000 -0.000497000  1 -9.689656000 -1.327192000 0.007573000  1 -7.210935000 -1.417458000 0.042126000  1 -9.530140000 2.993198000 -0.034770000  1 -10.851315000 0.878251000 -0.033093000  1 -1.498402000 3.487075000 0.019372000  1 -2.861847000 5.575947000 -0.004205000  1 -5.350716000 5.449725000 -0.011213000  16 -5.283383000 0.551697000 0.040270000  8 -7.519478000 4.355857000 -0.009094000 |
|  | **PhI**  E (Def2TZVPP): -529.4392186 A.U.  G_corr_ (Def2SVP): 0.059409 A.U  6 2.637528000 -1.206549000 -0.000001000  6 1.242165000 -1.214299000 0.000000000  6 0.553525000 -0.000002000 0.000000000  6 1.242163000 1.214298000 0.000000000  6 2.637524000 1.206552000 -0.000001000  6 3.336854000 0.000002000 -0.000001000  1 3.178706000 -2.155655000 -0.000001000  1 0.699200000 -2.161415000 0.000000000  1 0.699193000 2.161412000 0.000000000  1 3.178701000 2.155659000 -0.000001000  1 4.429172000 0.000004000 -0.000002000  53 -1.556726000 -0.000003000 0.000001000 |
|  | **1a‘**  E (Def2TZVPP): -2666.042855 A.U.  G_corr_ (Def2SVP): 0.098649 A.U.  16 -1.433126000 1.647355000 0.772521000  8 -2.258907000 2.814567000 0.986528000  8 -1.413549000 0.587099000 1.777970000  7 -1.588513000 1.119472000 -0.751814000  53 -1.849519000 -0.845157000 -0.976101000  6 0.096535000 -1.603140000 -0.692271000  6 2.680018000 -2.467465000 -0.254963000  6 1.027036000 -1.492313000 -1.725839000  6 0.426898000 -2.132710000 0.555144000  6 1.734044000 -2.570369000 0.764705000  6 2.329842000 -1.930675000 -1.494524000  1 0.749399000 -1.064020000 -2.690963000  1 -0.311182000 -2.186192000 1.357164000  1 2.013031000 -2.984268000 1.735775000  1 3.074661000 -1.847624000 -2.288647000  1 3.703972000 -2.804396000 -0.080202000  8 0.073901000 2.312345000 0.769801000  6 1.155547000 1.450655000 0.555033000  1 1.048034000 0.514554000 1.127287000  1 1.269127000 1.210696000 -0.515530000  6 2.431431000 2.136753000 1.032379000  17 2.707589000 3.652408000 0.135180000  17 2.337746000 2.489445000 2.776369000  17 3.791850000 1.014762000 0.726932000 |
|  | **1a‘^3^**  E (Def2TZVPP): -2666.000410 A.U.  G_corr_ (Def2SVP): 0.092133 A.U.  16 0.186399000 0.977910000 0.379366000  8 -0.039535000 -0.180547000 1.202380000  8 1.340448000 1.108726000 -0.484667000  7 -1.182186000 1.327862000 -0.518709000  53 -1.270715000 -0.994360000 -2.322138000  6 0.189087000 -2.218950000 -1.427154000  6 2.115789000 -3.799185000 -0.208331000  6 1.535760000 -1.994433000 -1.712730000  6 -0.209797000 -3.218036000 -0.538866000  6 0.764780000 -4.009341000 0.067647000  6 2.497766000 -2.794088000 -1.095911000  1 1.837674000 -1.202017000 -2.399002000  1 -1.265826000 -3.377222000 -0.313903000  1 0.460917000 -4.792669000 0.765549000  1 3.554622000 -2.622678000 -1.312025000  1 2.874564000 -4.419765000 0.273333000  8 0.150407000 2.224913000 1.408162000  6 0.734678000 3.467657000 1.063752000  1 1.226196000 3.423455000 0.080674000  1 -0.052448000 4.234933000 1.051151000  6 1.781670000 3.846304000 2.113793000  17 1.032891000 3.960639000 3.727069000  17 3.087762000 2.636304000 2.154026000  17 2.444963000 5.436368000 1.648466000 |
|  | **INT1^ET^**  E (Def2TZVPP): -2136.554018 A.U.  G_corr_ (Def2SVP): 0.015473 A.U.  16 1.834637000 -0.085071000 -0.062905000  8 1.700918000 -1.437715000 -0.527638000  8 2.089468000 0.250311000 1.317641000  7 3.027764000 0.663858000 -0.993952000  8 0.564721000 0.723878000 -0.613767000  6 -0.003400000 1.813513000 0.103424000  1 -0.044818000 2.674764000 -0.576408000  1 0.596722000 2.063272000 0.990253000  6 -1.423420000 1.451959000 0.545736000  17 -1.389413000 0.051365000 1.646940000  17 -2.435777000 1.069128000 -0.868355000  17 -2.084810000 2.873809000 1.393356000 |
|  | **TS1^ET^**  E (Def2TZVPP): -2372.452748 A.U.  G_corr_ (Def2SVP): 0.172089 A.U.  6 -0.602512000 -1.407403000 -0.161832000  6 0.829632000 -1.893690000 -0.208768000  6 1.849883000 -0.864102000 -0.638832000  6 1.691065000 0.454003000 0.120657000  6 0.250551000 0.965022000 0.072520000  6 -0.733845000 -0.083185000 0.593637000  1 2.869689000 -1.261441000 -0.526616000  1 1.075508000 -2.165066000 0.993989000  1 0.958810000 -2.881282000 -0.676385000  1 -1.255279000 -2.178779000 0.275598000  1 1.989243000 0.291589000 1.170835000  1 2.383376000 1.206513000 -0.287502000  1 0.158618000 1.893743000 0.657672000  1 -0.010309000 1.223442000 -0.969684000  1 -0.539155000 -0.263465000 1.665728000  1 -1.768162000 0.287323000 0.521393000  1 1.699625000 -0.689690000 -1.722222000  1 -0.938439000 -1.272536000 -1.208463000  16 2.871399000 -2.823588000 2.409426000  8 3.027133000 -4.133460000 1.823231000  8 3.696311000 -1.707697000 1.990540000  7 1.256626000 -2.454712000 2.342677000  8 3.078093000 -3.037228000 4.000918000  6 3.731162000 -2.075945000 4.804437000  1 4.343785000 -1.393970000 4.196592000  1 4.381282000 -2.631369000 5.494301000  6 2.739692000 -1.254118000 5.635392000  17 1.688598000 -2.322446000 6.594483000  17 1.728174000 -0.220352000 4.586616000  17 3.703849000 -0.218093000 6.723110000 |
|  | **Cyclohexyl radical**  E (Def2TZVPP): -235.2352413 A.U.  G_corr_ (Def2SVP): 0.126481 A.U.  6 0.000012000 -1.462101000 -0.168005000  6 1.286785000 -0.772284000 0.148806000  6 1.262036000 0.712317000 -0.242357000  6 -0.000012000 1.405775000 0.272044000  6 -1.262049000 0.712296000 -0.242357000  6 -1.286772000 -0.772306000 0.148806000  1 2.163667000 1.217156000 0.139726000  1 1.475926000 -0.837319000 1.243572000  1 2.135736000 -1.286314000 -0.329801000  1 0.000022000 -2.541702000 -0.348993000  1 -0.000012000 1.392241000 1.377555000  1 -0.000021000 2.465951000 -0.028348000  1 -2.163689000 1.217119000 0.139727000  1 -1.293524000 0.796017000 -1.342882000  1 -1.475912000 -0.837345000 1.243572000  1 -2.135714000 -1.286350000 -0.329801000  1 1.293509000 0.796039000 -1.342883000 |
|  | **NitreneHAT**  E (Def2TZVPP): -2137.220889 A.U.  G_corr_ (Def2SVP): 0.027267 A.U.  1 1.080373000 -2.131838000 1.413322000  16 2.892538000 -2.825398000 2.405089000  8 3.062520000 -4.124595000 1.801143000  8 3.678470000 -1.686394000 1.983684000  7 1.269952000 -2.514603000 2.356325000  8 3.094645000 -3.047051000 3.984633000  6 3.736646000 -2.086239000 4.801067000  1 4.369541000 -1.412255000 4.205451000  1 4.364104000 -2.647028000 5.506802000  6 2.729928000 -1.253596000 5.602382000  17 1.652852000 -2.308902000 6.545752000  17 1.749100000 -0.226486000 4.518522000  17 3.674199000 -0.212458000 6.701558000 |
|  | **INT1^SET^**  E (Def2TZVPP): -2666.192616 A.U.  G_corr_ (Def2SVP): 0.094092 A.U.  7 -1.022130000 -1.951118000 -2.171323000  53 1.952577000 1.650686000 1.466904000  6 0.619327000 2.089448000 -0.109846000  6 -1.180670000 2.641822000 -2.155939000  6 0.400222000 1.137162000 -1.104875000  6 -0.047786000 3.315795000 -0.125160000  6 -0.947183000 3.587060000 -1.156623000  6 -0.508441000 1.421002000 -2.126185000  1 0.893481000 0.162017000 -1.077517000  1 0.125314000 4.054254000 0.660170000  1 -1.473436000 4.544642000 -1.170476000  1 -0.701593000 0.655200000 -2.880883000  1 -1.894505000 2.854324000 -2.955054000  16 -0.294869000 -2.742402000 -1.053367000  8 -0.403794000 -4.194023000 -1.143499000  8 1.031090000 -2.157475000 -0.787516000  8 -1.107746000 -2.509729000 0.427004000  6 -1.235315000 -1.182116000 0.804811000  1 -0.355492000 -0.818397000 1.365873000  1 -1.391298000 -0.528578000 -0.072161000  6 -2.457564000 -1.003153000 1.699033000  17 -2.550370000 0.727813000 2.155593000  17 -3.948801000 -1.461365000 0.833939000  17 -2.317233000 -1.988321000 3.184014000 |
|  | **INT2^SET^**  E (Def2TZVPP): -2136.744790 A.U.  G_corr_ (Def2SVP): 0.015892 A.U.  7 -1.022130000 -1.951118000 -2.171323000  53 1.952577000 1.650686000 1.466904000  6 0.619327000 2.089448000 -0.109846000  6 -1.180670000 2.641822000 -2.155939000  6 0.400222000 1.137162000 -1.104875000  6 -0.047786000 3.315795000 -0.125160000  6 -0.947183000 3.587060000 -1.156623000  6 -0.508441000 1.421002000 -2.126185000  1 0.893481000 0.162017000 -1.077517000  1 0.125314000 4.054254000 0.660170000  1 -1.473436000 4.544642000 -1.170476000  1 -0.701593000 0.655200000 -2.880883000  1 -1.894505000 2.854324000 -2.955054000  16 -0.294869000 -2.742402000 -1.053367000  8 -0.403794000 -4.194023000 -1.143499000  8 1.031090000 -2.157475000 -0.787516000  8 -1.107746000 -2.509729000 0.427004000  6 -1.235315000 -1.182116000 0.804811000  1 -0.355492000 -0.818397000 1.365873000  1 -1.391298000 -0.528578000 -0.072161000  6 -2.457564000 -1.003153000 1.699033000  17 -2.550370000 0.727813000 2.155593000  17 -3.948801000 -1.461365000 0.833939000  17 -2.317233000 -1.988321000 3.184014000 |
|  | **TS1^SET^**  E (Def2TZVPP): -2372.633274 A.U.  G_corr_ (Def2SVP): 0.174964 A.U.  6 -0.055017000 -1.357006000 -0.477753000  6 1.346230000 -1.638114000 0.026660000  6 2.174209000 -0.396280000 0.285779000  6 1.431819000 0.605519000 1.173619000  6 0.047156000 0.927446000 0.609108000  6 -0.781372000 -0.343165000 0.411033000  1 3.146244000 -0.670176000 0.727248000  1 1.146116000 -2.243212000 1.146473000  1 1.880458000 -2.407537000 -0.548546000  1 -0.628489000 -2.296479000 -0.534080000  1 1.312061000 0.174278000 2.182673000  1 2.026062000 1.526168000 1.291667000  1 -0.482400000 1.626111000 1.277252000  1 0.160975000 1.445409000 -0.361554000  1 -0.969340000 -0.806119000 1.394626000  1 -1.765782000 -0.096713000 -0.019482000  1 2.407857000 0.086765000 -0.684476000  1 0.002835000 -0.961365000 -1.511934000  16 2.021749000 -3.814762000 2.598923000  8 1.617215000 -5.035602000 3.291760000  8 3.069960000 -3.940438000 1.572718000  7 0.825696000 -2.892409000 2.232037000  8 2.877151000 -3.066869000 3.894974000  6 3.383085000 -1.818708000 3.603785000  1 2.853955000 -1.349514000 2.756231000  1 4.462573000 -1.846006000 3.367141000  6 3.203500000 -0.874893000 4.794046000  17 4.067101000 -1.493887000 6.232882000  17 1.479492000 -0.675059000 5.193818000  17 3.893304000 0.720632000 4.346821000 |
|  | **NitreneAnionHAT**  E (Def2TZVPP): -2137.418197 A.U.  G_corr_ (Def2SVP): 0.029051 A.U.  16 2.211627000 0.189669000 -0.121359000  8 2.299177000 -1.196794000 -0.582827000  8 2.911442000 0.527855000 1.120020000  7 2.378614000 1.311525000 -1.174419000  8 0.555601000 0.186540000 0.363751000  6 0.072939000 1.409600000 0.785188000  1 0.636614000 2.244044000 0.328664000  1 0.102852000 1.520903000 1.884777000  6 -1.384894000 1.571992000 0.353938000  17 -2.409573000 0.299495000 1.081652000  17 -1.535840000 1.488019000 -1.423233000  17 -1.964377000 3.172690000 0.916911000  1 1.949418000 0.972317000 -2.038963000 |
|  | **Cyclohexyl cation**  E (Def2TZVPP): -235.057128 A.U.  G_corr_ (Def2SVP): 0.1272 A.U.  6 0.000012000 -1.425666000 0.012851000  6 1.267437000 -0.747281000 0.049922000  6 1.260909000 0.747343000 -0.254304000  6 -0.000013000 1.424256000 0.281938000  6 -1.260922000 0.747321000 -0.254305000  6 -1.267425000 -0.747303000 0.049924000  1 2.166237000 1.202761000 0.169158000  1 1.508564000 -0.953298000 1.130346000  1 2.045127000 -1.344324000 -0.456227000  1 0.000021000 -2.522411000 0.070646000  1 -0.000013000 1.389121000 1.385111000  1 -0.000022000 2.486919000 0.002507000  1 -2.166258000 1.202724000 0.169156000  1 -1.321907000 0.878342000 -1.346234000  1 -1.508546000 -0.953321000 1.130348000  1 -2.045106000 -1.344360000 -0.456224000  1 1.321892000 0.878367000 -1.346233000 |
|  | **TS1^TXO^**  E (Def2TZVPP): -1209.452915 A.U.  G_corr_ (Def2SVP): 0.289279 A.U.  6 0.800253000 -2.101721000 0.232222000  6 1.799007000 -0.958261000 0.243359000  6 1.215342000 0.378858000 -0.180780000  6 -0.074582000 0.701206000 0.575358000  6 -1.093287000 -0.432668000 0.453913000  6 -0.510194000 -1.758582000 0.943663000  1 1.960058000 1.178747000 -0.044339000  1 2.182668000 -0.779117000 1.342235000  1 2.727270000 -1.207485000 -0.294964000  1 1.249858000 -3.010886000 0.660905000  1 0.167246000 0.856978000 1.640946000  1 -0.502486000 1.646134000 0.204956000  1 -2.004233000 -0.187713000 1.022858000  1 -1.399631000 -0.534912000 -0.603197000  1 -0.327125000 -1.691728000 2.029518000  1 -1.234960000 -2.575668000 0.801579000  1 1.003546000 0.331709000 -1.266357000  1 0.586441000 -2.336941000 -0.828582000  6 2.479845000 -5.288403000 3.395559000  6 1.446621000 -4.786198000 4.181543000  6 1.205870000 -3.411615000 4.243309000  6 2.005183000 -2.498755000 3.504997000  6 3.067095000 -3.040483000 2.735051000  6 3.296523000 -4.404090000 2.679132000  6 1.782479000 -1.083759000 3.539780000  6 0.935642000 -0.424734000 4.487394000  6 0.073831000 -1.143499000 5.359856000  6 -0.716990000 -0.470313000 6.293419000  1 -1.370049000 -1.041188000 6.958653000  6 -0.685844000 0.919771000 6.375120000  6 0.138662000 1.647508000 5.508000000  6 0.933185000 0.991095000 4.583528000  1 2.655948000 -6.365016000 3.352979000  1 0.818031000 -5.469362000 4.758639000  1 3.714000000 -2.352683000 2.189447000  1 4.124647000 -4.788047000 2.079314000  1 -1.310103000 1.433706000 7.108737000  1 0.156600000 2.738493000 5.557911000  1 1.576832000 1.555518000 3.907652000  16 -0.150918000 -2.884415000 5.232646000  8 2.491038000 -0.285758000 2.716722000 |
|  | **Toluene**  E (Def2TZVPP): -271.578447 A.U.  G_corr_ (Def2SVP): 0.099761 A.U.  6 -0.419278000 -0.117209000 -3.303716000  6 -1.577551000 -0.465872000 -2.606810000  6 0.737556000 0.233628000 -2.609758000  1 -2.480279000 -0.738433000 -3.161039000  1 1.644874000 0.507722000 -3.153355000  6 -1.603560000 -0.471707000 -1.207173000  6 0.726177000 0.233204000 -1.213667000  1 1.627007000 0.508010000 -0.659065000  6 -0.432471000 -0.115591000 -0.522605000  1 -0.431741000 -0.111890000 0.571485000  1 -0.423037000 -0.119135000 -4.396662000  6 -2.844172000 -0.860978000 -0.446900000  1 -3.707092000 -0.982318000 -1.116911000  1 -2.695974000 -1.813860000 0.086558000  1 -3.102284000 -0.103365000 0.309148000 |
|  | **TS1Tol^ET^**  E (Def2TZVPP): -2408.124424 A.U.  G_corr_ (Def2SVP): 0.130884 A.U.  6 0.598323000 1.491019000 -2.620674000  6 -0.717158000 1.089245000 -2.825012000  6 1.618107000 0.538707000 -2.551491000  1 -1.515105000 1.834831000 -2.875907000  1 2.651266000 0.854823000 -2.389640000  6 -1.036982000 -0.273378000 -2.969347000  6 1.314971000 -0.817691000 -2.687632000  1 2.110548000 -1.564172000 -2.631952000  6 -0.000380000 -1.221479000 -2.889433000  1 -0.240803000 -2.282672000 -2.983806000  1 0.832931000 2.552491000 -2.513236000  6 -2.431224000 -0.700837000 -3.143537000  1 -2.567188000 -1.699543000 -3.576800000  1 -3.102539000 0.048424000 -3.584355000  16 -2.436167000 -1.829636000 0.170924000  8 -1.290857000 -0.977815000 0.383241000  8 -3.122774000 -2.464955000 1.274359000  7 -3.523993000 -0.949172000 -0.719765000  8 -1.929532000 -2.997862000 -0.842753000  6 -2.411234000 -4.321459000 -0.739801000  1 -3.011343000 -4.456674000 0.172027000  1 -1.541897000 -4.992928000 -0.703152000  6 -3.262972000 -4.691393000 -1.956850000  17 -2.304734000 -4.569732000 -3.459282000  17 -4.680973000 -3.613667000 -2.071978000  17 -3.803914000 -6.374284000 -1.740507000  1 -2.933978000 -0.822685000 -2.026235000 |
|  | **TS1Tol^SET^**  E (Def2TZVPP): -2408.309591 A.U.  G_corr_ (Def2SVP): 0.131271 A.U.  6 0.183672000 2.174662000 -3.198923000  6 -0.803956000 1.192207000 -3.258559000  6 1.454408000 1.864061000 -2.713277000  1 -1.798536000 1.444634000 -3.637567000  1 2.228786000 2.633701000 -2.664000000  6 -0.541737000 -0.123656000 -2.842040000  6 1.725695000 0.562457000 -2.283141000  1 2.714679000 0.312345000 -1.890078000  6 0.741079000 -0.419622000 -2.344579000  1 0.935559000 -1.430907000 -1.977125000  1 -0.041750000 3.191072000 -3.532562000  6 -1.588012000 -1.164465000 -2.863824000  1 -1.238707000 -2.164019000 -3.151246000  1 -2.510188000 -0.879821000 -3.387539000  16 -1.477570000 -3.068932000 -0.181328000  8 -0.163150000 -3.254040000 -0.799059000  8 -1.623555000 -3.486716000 1.217467000  7 -2.213151000 -1.716465000 -0.408205000  8 -2.326504000 -4.276911000 -1.045703000  6 -3.655948000 -4.421265000 -0.686974000  1 -4.095832000 -3.459909000 -0.363571000  1 -3.792544000 -5.162232000 0.120975000  6 -4.467521000 -4.903395000 -1.888221000  17 -3.848349000 -6.470020000 -2.486104000  17 -4.413915000 -3.713847000 -3.217737000  17 -6.168818000 -5.113764000 -1.364232000  1 -1.953731000 -1.357644000 -1.655441000 |
|  | **TS1Tol^TXO^**  E (Def2TZVPP): -1245.133026 A.U.  G_corr_ (Def2SVP): 0.247833 A.U.  6 -1.306668000 2.173859000 -0.688300000  6 -2.264886000 1.223609000 -1.008546000  6 -0.196134000 1.818309000 0.085536000  1 -3.139562000 1.505736000 -1.599475000  1 0.553827000 2.569508000 0.342855000  6 -2.133654000 -0.116056000 -0.570885000  6 -0.037008000 0.494942000 0.512061000  1 0.834188000 0.213167000 1.107443000  6 -0.992764000 -0.458490000 0.193721000  1 -0.875231000 -1.488061000 0.540153000  1 -1.427457000 3.203873000 -1.030857000  6 -3.178746000 -1.098748000 -0.838952000  1 -3.803793000 -0.878556000 -1.715266000  1 -2.843887000 -2.144885000 -0.815821000  1 -3.975388000 -1.049370000 0.082519000  6 -0.862120000 -1.069055000 4.383362000  6 -1.045951000 0.282874000 4.118885000  6 -2.093790000 0.707140000 3.294650000  6 -2.991595000 -0.227233000 2.707609000  6 -2.773389000 -1.600398000 3.000163000  6 -1.736706000 -2.010065000 3.816742000  6 -4.056763000 0.147217000 1.826477000  6 -4.323222000 1.502596000 1.448045000  6 -3.557668000 2.610154000 1.907306000  6 -3.836271000 3.910879000 1.473847000  1 -3.226305000 4.742848000 1.836029000  6 -4.876233000 4.147917000 0.582958000  6 -5.648130000 3.070921000 0.118342000  6 -5.378327000 1.782505000 0.538569000  1 -0.041635000 -1.391051000 5.027576000  1 -0.365764000 1.021599000 4.551332000  1 -3.456396000 -2.322991000 2.553232000  1 -1.597559000 -3.074156000 4.021813000  1 -5.086811000 5.166241000 0.250491000  1 -6.467826000 3.251293000 -0.581117000  1 -5.969646000 0.939345000 0.181036000  16 -2.196880000 2.427978000 2.992646000  8 -4.763613000 -0.819687000 1.246037000 |
|  | **4a‘**  E (Def2TZVPP): -1405.187198 A.U.  G_corr_ (Def2SVP): 0.213408 A.U.  7 -2.404575000 1.117590000 0.100173000  53 -3.255365000 2.174222000 -1.371370000  6 -1.701792000 2.385148000 -2.784318000  6 0.413521000 2.565727000 -4.544818000  6 -0.667822000 3.277257000 -2.501636000  6 -1.705721000 1.581851000 -3.923800000  6 -0.635941000 1.686038000 -4.811929000  6 0.399036000 3.356159000 -3.395243000  1 -0.677635000 3.878255000 -1.590690000  1 -2.515948000 0.875594000 -4.113809000  1 -0.619295000 1.065029000 -5.709999000  1 1.224092000 4.040102000 -3.186268000  1 1.254593000 2.632624000 -5.238287000  16 -1.408982000 1.965886000 1.064578000  8 -1.670682000 3.399363000 1.095166000  8 -1.339913000 1.220138000 2.312184000  8 0.073919000 1.900719000 0.387572000  6 0.920417000 0.771547000 0.643209000  1 0.978209000 0.606794000 1.729576000  6 0.473293000 -0.494304000 -0.066404000  1 -0.503280000 -0.794872000 0.344960000  1 1.185634000 -1.294502000 0.203266000  6 0.370838000 -0.372147000 -1.590751000  1 -0.187562000 0.552841000 -1.797265000  1 1.909918000 1.102951000 0.297338000  6 1.739251000 -0.253018000 -2.260060000  1 1.635897000 -0.140493000 -3.350851000  1 2.303156000 0.619727000 -1.895323000  1 2.351570000 -1.152035000 -2.072786000  6 -0.433926000 -1.532054000 -2.171749000  1 -1.451173000 -1.554962000 -1.749095000  1 -0.523042000 -1.448859000 -3.266986000  1 0.044058000 -2.501631000 -1.949520000 |
|  | **4a‘^3^**  E (Def2TZVPP): -1405.151584 A.U.  G_corr_ (Def2SVP): 0.211345 A.U.  7 -2.162878000 0.833331000 0.936879000  53 -3.320250000 1.805087000 -2.068023000  6 -1.527622000 2.319487000 -3.051792000  6 0.843323000 2.989739000 -4.341474000  6 -0.638494000 3.202135000 -2.437757000  6 -1.246243000 1.768528000 -4.302852000  6 -0.055688000 2.110797000 -4.944722000  6 0.549787000 3.531874000 -3.090387000  1 -0.860209000 3.620582000 -1.455116000  1 -1.945169000 1.076541000 -4.776554000  1 0.167828000 1.681095000 -5.923929000  1 1.248748000 4.221223000 -2.611051000  1 1.774582000 3.252943000 -4.847931000  16 -1.018840000 1.994974000 1.384940000  8 -1.581230000 3.301195000 1.148324000  8 -0.544794000 1.598070000 2.693703000  8 0.110524000 1.786963000 0.296043000  6 1.122961000 0.752498000 0.445492000  1 1.497433000 0.779734000 1.478089000  6 0.592597000 -0.615830000 0.074761000  1 -0.162321000 -0.918815000 0.822358000  1 1.432528000 -1.323225000 0.190861000  6 -0.000635000 -0.734884000 -1.334731000  1 -0.809479000 0.010865000 -1.418585000  1 1.921962000 1.091051000 -0.225484000  6 1.025455000 -0.430963000 -2.425107000  1 0.590412000 -0.590152000 -3.423306000  1 1.373445000 0.613073000 -2.390528000  1 1.906734000 -1.089344000 -2.335576000  6 -0.625963000 -2.114668000 -1.526202000  1 -1.395012000 -2.314753000 -0.763473000  1 -1.105009000 -2.197033000 -2.514184000  1 0.135126000 -2.910158000 -1.454642000 |
|  | **INT1^IntraET^**  E (Def2TZVPP): -875.703390 A.U.  G_corr_ (Def2SVP): 0.129437 A.U.  16 2.128557000 0.299935000 -0.091553000  8 2.523892000 -1.082594000 -0.168502000  8 2.496534000 1.144161000 1.023454000  7 2.706362000 1.054556000 -1.488926000  8 0.567060000 0.346789000 -0.343630000  6 -0.222791000 1.501302000 0.067870000  1 0.032127000 1.739980000 1.109369000  6 -0.017637000 2.692436000 -0.844270000  1 1.015374000 3.062682000 -0.719599000  1 -0.671106000 3.495828000 -0.461350000  6 -0.305767000 2.455570000 -2.332763000  1 0.336442000 1.622908000 -2.671582000  1 -1.249798000 1.117853000 0.038681000  6 -1.759468000 2.059382000 -2.587959000  1 -1.943012000 1.924598000 -3.665037000  1 -2.027712000 1.113178000 -2.093189000  1 -2.450663000 2.839347000 -2.225951000  6 0.081876000 3.693167000 -3.140035000  1 1.142368000 3.951457000 -2.992672000  1 -0.077644000 3.530030000 -4.217054000  1 -0.522090000 4.566034000 -2.839957000 |
|  | **TS1^IntraET^**  E (Def2TZVPP): -875.687655 A.U.  G_corr_ (Def2SVP): 0.126208 A.U.  16 1.798636000 0.343646000 -0.583448000  8 2.093058000 -1.067327000 -0.601065000  8 2.579757000 1.271786000 0.213295000  7 1.872108000 0.906042000 -2.148019000  8 0.258281000 0.435671000 -0.158748000  6 -0.330367000 1.677836000 0.289075000  1 0.044302000 1.901986000 1.298036000  6 -0.092756000 2.854520000 -0.649399000  1 0.892905000 3.302334000 -0.449305000  1 -0.841021000 3.625175000 -0.392146000  6 -0.192306000 2.532241000 -2.131325000  1 0.769300000 1.734391000 -2.285627000  1 -1.396458000 1.428556000 0.361517000  6 -1.421927000 1.765833000 -2.565677000  1 -1.392122000 1.548115000 -3.643177000  1 -1.531279000 0.813437000 -2.027136000  1 -2.325274000 2.370782000 -2.369359000  6 0.184877000 3.690896000 -3.027862000  1 1.143926000 4.139785000 -2.729204000  1 0.257996000 3.376059000 -4.079249000  1 -0.588732000 4.476837000 -2.965833000 |
|  | **INT2^IntraET^**  E (Def2TZVPP): -875.704094 A.U.  G_corr_ (Def2SVP): 0.126809 A.U.  16 1.952847000 0.355022000 -0.393602000  8 2.257548000 -1.050749000 -0.473128000  8 2.561117000 1.206158000 0.609277000  7 2.349287000 1.017233000 -1.856260000  8 0.360112000 0.435293000 -0.266870000  6 -0.284623000 1.636795000 0.217578000  1 0.047438000 1.824886000 1.248590000  6 -0.042509000 2.855447000 -0.657999000  1 0.983754000 3.230168000 -0.499617000  1 -0.698944000 3.654064000 -0.252839000  6 -0.299303000 2.682060000 -2.129434000  1 -1.344856000 1.355103000 0.240259000  6 -1.434801000 1.824094000 -2.586612000  1 -1.433320000 1.706683000 -3.680585000  1 -1.411586000 0.818908000 -2.136671000  1 -2.410619000 2.274321000 -2.310376000  6 0.189691000 3.770453000 -3.030437000  1 1.176424000 4.150242000 -2.720258000  1 0.256844000 3.435923000 -4.077662000  1 -0.503331000 4.637137000 -3.017182000  1 1.497441000 1.464121000 -2.262179000 |
|  | **5a**  E (Def2TZVPP): -875.833588 A.U.  G_corr_ (Def2SVP): 0.138037 A.U.  16 1.608744000 0.529437000 -0.700871000  8 1.892179000 -0.837423000 -1.060504000  8 2.561892000 1.246316000 0.129999000  7 1.233820000 1.419140000 -2.053174000  8 0.177615000 0.506127000 0.007808000  6 -0.408579000 1.761849000 0.422067000  1 -0.051460000 2.002058000 1.433646000  6 -0.106683000 2.882608000 -0.566640000  1 0.814361000 3.408893000 -0.273673000  1 -0.927028000 3.614187000 -0.533130000  6 0.074035000 2.362336000 -2.004934000  1 -1.481833000 1.539203000 0.472592000  6 -1.174022000 1.638740000 -2.516007000  1 -1.027702000 1.332634000 -3.562181000  1 -1.390955000 0.734582000 -1.930685000  1 -2.049070000 2.304711000 -2.467000000  6 0.421442000 3.522489000 -2.934005000  1 1.300987000 4.075442000 -2.568852000  1 0.633794000 3.154448000 -3.949459000  1 -0.423440000 4.223415000 -2.994047000  1 2.090513000 1.862172000 -2.386959000 |
|  | ***cis*-1,4-dimethylcyclohexane**  E (Def2TZVPP): -314.5421413 A.U.  G_corr_ (Def2SVP): 0.194937 A.U.  6 -0.592725000 -1.367808000 -0.156103000  6 0.852653000 -1.877913000 -0.111543000  6 1.806192000 -0.820586000 -0.680900000  6 1.646880000 0.533811000 0.012382000  6 0.201809000 1.057416000 -0.024804000  6 -0.748404000 -0.012812000 0.536796000  1 2.848476000 -1.168437000 -0.586032000  1 1.118289000 -2.021511000 0.953466000  1 -1.263702000 -2.106566000 0.313360000  1 1.956775000 0.427914000 1.067128000  1 2.327894000 1.274555000 -0.438950000  1 0.147546000 1.935084000 0.641947000  1 -0.537994000 -0.141780000 1.613291000  1 -1.792566000 0.334311000 0.463339000  1 1.616248000 -0.715913000 -1.764522000  1 -0.911775000 -1.292381000 -1.211475000  6 1.002972000 -3.218664000 -0.822661000  1 2.036208000 -3.595531000 -0.756929000  1 0.338623000 -3.982065000 -0.387009000  1 0.749083000 -3.127262000 -1.892299000  6 -0.211655000 1.529634000 -1.420373000  1 -1.235595000 1.936231000 -1.410927000  1 0.459988000 2.324634000 -1.781940000  1 -0.188317000 0.717751000 -2.163830000 |
|  | ***trans*-1,4-dimethylcyclohexane**  E (Def2TZVPP): -314.5445331 A.U.  G_corr_ (Def2SVP): 0.194509 A.U.  6 -0.577602000 -1.436339000 -0.172296000  6 0.870647000 -1.928758000 -0.082611000  6 1.815554000 -0.890144000 -0.696156000  6 1.634469000 0.495579000 -0.078243000  6 0.186220000 0.987999000 -0.167929000  6 -0.758687000 -0.050616000 0.445616000  1 2.861359000 -1.222153000 -0.583205000  1 1.125329000 -2.014781000 0.991523000  1 -1.250507000 -2.160639000 0.316877000  1 1.932469000 0.462829000 0.986830000  1 2.307374000 1.219880000 -0.567416000  1 -0.068462000 1.074021000 -1.242062000  1 -0.564962000 -0.107136000 1.533527000  1 -1.804492000 0.281394000 0.332665000  1 1.621829000 -0.833623000 -1.784066000  1 -0.875602000 -1.403589000 -1.237369000  6 1.043749000 -3.301856000 -0.723587000  1 2.080480000 -3.662883000 -0.627305000  1 0.382871000 -4.049915000 -0.256607000  1 0.801059000 -3.267722000 -1.799496000  6 0.013118000 2.361096000 0.473047000  1 0.673997000 3.109155000 0.006068000  1 -1.023613000 2.722124000 0.376765000  1 0.255808000 2.326963000 1.548956000 |
|  | **TSAx_*cis***  E (Def2TZVPP): -2451.094906 A.U.  G_corr_ (Def2SVP): 0.225185 A.U.  6 -0.587712000 -1.456750000 -0.185600000  6 0.845748000 -1.965310000 -0.224736000  6 1.849842000 -0.907796000 -0.652472000  6 1.686138000 0.403455000 0.116515000  6 0.248676000 0.943871000 0.079084000  6 -0.721834000 -0.137970000 0.577735000  1 2.873880000 -1.293303000 -0.532349000  1 1.071769000 -2.158140000 0.955760000  1 -1.243768000 -2.223882000 0.256137000  1 1.977901000 0.225757000 1.165418000  1 2.387226000 1.156581000 -0.277744000  1 0.193401000 1.789342000 0.785521000  1 -0.521968000 -0.330683000 1.645968000  1 -1.760626000 0.224418000 0.515423000  1 1.707829000 -0.740194000 -1.737018000  1 -0.923142000 -1.332384000 -1.232693000  16 2.844708000 -2.877705000 2.443790000  8 2.961532000 -4.217236000 1.917792000  8 3.687211000 -1.801649000 1.961658000  7 1.232788000 -2.480596000 2.387648000  8 3.071437000 -3.024333000 4.039978000  6 3.736140000 -2.031787000 4.795074000  1 4.343435000 -1.377608000 4.152239000  1 4.392730000 -2.560179000 5.499926000  6 2.754798000 -1.173187000 5.600634000  17 1.704390000 -2.197533000 6.607035000  17 1.741704000 -0.176809000 4.517083000  17 3.730044000 -0.099932000 6.640690000  6 1.026214000 -3.317334000 -0.877658000  1 2.059983000 -3.679802000 -0.777273000  1 0.351358000 -4.068887000 -0.440777000  1 0.797642000 -3.245189000 -1.955212000  6 -0.136343000 1.481549000 -1.300396000  1 -1.156265000 1.897584000 -1.290533000  1 0.549431000 2.283950000 -1.615411000  1 -0.107665000 0.702383000 -2.078498000 |
|  | **TSEq_*cis***  E (Def2TZVPP): -2451.094045 A.U.  G_corr_ (Def2SVP): 0.224654 A.U.  6 -0.520123000 -1.302795000 -0.735074000  6 0.808161000 -1.893849000 -0.283023000  6 1.957946000 -0.914998000 -0.481287000  6 1.672513000 0.412705000 0.234667000  6 0.339222000 1.030715000 -0.201229000  6 -0.800776000 0.022875000 -0.015635000  1 2.901843000 -1.352528000 -0.120731000  1 1.039441000 -2.764584000 -1.094081000  1 -1.334976000 -2.022430000 -0.565538000  1 1.653798000 0.249188000 1.326975000  1 2.497731000 1.116679000 0.039358000  1 0.414055000 1.249086000 -1.283383000  1 -0.947072000 -0.163118000 1.063461000  1 -1.746410000 0.448167000 -0.389241000  1 2.081814000 -0.718459000 -1.559833000  1 -0.476333000 -1.113191000 -1.821303000  16 0.417481000 -5.070128000 -1.472036000  8 1.311536000 -5.688252000 -0.521923000  8 -0.941581000 -4.720881000 -1.110523000  7 1.244048000 -3.788351000 -2.132333000  8 0.365032000 -6.079704000 -2.736703000  6 -0.813642000 -6.268080000 -3.493167000  1 -1.711199000 -6.027232000 -2.904746000  1 -0.834195000 -7.330039000 -3.773463000  6 -0.818078000 -5.432590000 -4.779081000  17 0.657000000 -5.734261000 -5.726128000  17 -0.933872000 -3.688021000 -4.410177000  17 -2.254311000 -5.927056000 -5.716366000  6 0.782609000 -2.587981000 1.063808000  1 -0.031954000 -3.324965000 1.119919000  1 1.734264000 -3.101189000 1.267325000  1 0.621734000 -1.854949000 1.872299000  6 0.059449000 2.339657000 0.530175000  1 0.866201000 3.071359000 0.365937000  1 -0.883010000 2.796057000 0.188616000  1 -0.024546000 2.173168000 1.617230000 |
|  | **INT1Ax_*cis***  E (Def2TZVPP): -2451.109191 A.U.  G_corr_ (Def2SVP): 0.225738 A.U.  6 -0.632106000 -1.452675000 -0.275266000  6 0.780796000 -1.931489000 -0.450721000  6 1.834753000 -0.894323000 -0.711761000  6 1.669975000 0.363690000 0.146993000  6 0.238099000 0.916999000 0.129951000  6 -0.738623000 -0.181231000 0.574849000  1 2.836711000 -1.326196000 -0.556562000  1 1.107197000 -2.310153000 1.428655000  1 -1.253173000 -2.253933000 0.159222000  1 1.945429000 0.113689000 1.185326000  1 2.382556000 1.136191000 -0.184361000  1 0.186640000 1.728352000 0.875821000  1 -0.524818000 -0.438625000 1.626825000  1 -1.773882000 0.195718000 0.551754000  1 1.794567000 -0.621200000 -1.788165000  1 -1.063632000 -1.264091000 -1.281812000  16 2.902670000 -2.931666000 2.501693000  8 3.043127000 -4.281198000 2.006200000  8 3.727079000 -1.856508000 1.988503000  7 1.295328000 -2.587082000 2.433677000  8 3.123743000 -3.030962000 4.094161000  6 3.763015000 -2.006819000 4.828908000  1 4.416290000 -1.398284000 4.186581000  1 4.369505000 -2.509003000 5.594690000  6 2.758649000 -1.089443000 5.537190000  17 1.623762000 -2.042881000 6.519156000  17 1.837947000 -0.110102000 4.358210000  17 3.702148000 0.002842000 6.587226000  6 1.011016000 -3.321846000 -0.949225000  1 2.042168000 -3.660871000 -0.761127000  1 0.318173000 -4.044269000 -0.488931000  1 0.851054000 -3.377217000 -2.045481000  6 -0.139051000 1.520021000 -1.224282000  1 -1.155956000 1.942810000 -1.198010000  1 0.553717000 2.330584000 -1.500864000  1 -0.114041000 0.776282000 -2.036386000 |
|  | **INT1Eq_*cis***  E (Def2TZVPP): -2451.107993 A.U.  G_corr_ (Def2SVP): 0.225729 A.U.  6 -0.475076000 -1.347100000 -0.585147000  6 0.841963000 -1.830485000 -0.046996000  6 1.986382000 -0.887700000 -0.289643000  6 1.656188000 0.505719000 0.280173000  6 0.329425000 1.048794000 -0.264036000  6 -0.803469000 0.045873000 -0.014542000  1 2.919230000 -1.271606000 0.151724000  1 1.211798000 -3.139147000 -1.421051000  1 -1.281353000 -2.061471000 -0.361365000  1 1.593661000 0.442965000 1.381658000  1 2.475705000 1.206353000 0.049938000  1 0.440556000 1.156295000 -1.359975000  1 -0.976588000 -0.038223000 1.073623000  1 -1.742919000 0.417450000 -0.455989000  1 2.159959000 -0.780881000 -1.376813000  1 -0.418046000 -1.259551000 -1.686613000  16 0.434352000 -5.165601000 -1.551423000  8 1.321976000 -5.848667000 -0.639660000  8 -0.906007000 -4.788887000 -1.150571000  7 1.292240000 -3.897546000 -2.156071000  8 0.333521000 -6.111661000 -2.851652000  6 -0.869323000 -6.257991000 -3.579180000  1 -1.745929000 -6.020750000 -2.958736000  1 -0.916016000 -7.309822000 -3.891653000  6 -0.893371000 -5.381007000 -4.836723000  17 0.546334000 -5.680889000 -5.837090000  17 -0.962423000 -3.647548000 -4.408918000  17 -2.366474000 -5.816772000 -5.746230000  6 0.867851000 -2.716060000 1.158424000  1 0.068944000 -3.472676000 1.127381000  1 1.835474000 -3.231787000 1.266427000  1 0.716958000 -2.128387000 2.086958000  6 0.003859000 2.421140000 0.317281000  1 0.805322000 3.146610000 0.105991000  1 -0.932199000 2.822581000 -0.102142000  1 -0.117105000 2.366460000 1.412205000 |
|  | **TSAx_*trans***  E (Def2TZVPP): -2451.097385 A.U.  G_corr_ (Def2SVP): 0.223395 A.U.  6 -0.584455000 -1.457664000 -0.283352000  6 0.850817000 -1.961088000 -0.278611000  6 1.855876000 -0.899825000 -0.692506000  6 1.670714000 0.413292000 0.064259000  6 0.233107000 0.937627000 -0.012537000  6 -0.746296000 -0.137991000 0.469560000  1 2.880194000 -1.280025000 -0.560542000  1 1.045986000 -2.139558000 0.911772000  1 -1.253833000 -2.227528000 0.132738000  1 1.941603000 0.254744000 1.123613000  1 2.371434000 1.169459000 -0.324873000  1 0.008659000 1.140189000 -1.077130000  1 -0.579365000 -0.313982000 1.547901000  1 -1.782952000 0.222223000 0.369777000  1 1.719352000 -0.728500000 -1.778007000  1 -0.881820000 -1.323281000 -1.341404000  16 2.756186000 -2.896241000 2.447648000  8 2.807741000 -4.273270000 2.016264000  8 3.656272000 -1.901259000 1.900189000  7 1.165243000 -2.430264000 2.347455000  8 2.962305000 -2.939501000 4.053177000  6 3.683861000 -1.936398000 4.739046000  1 4.327777000 -1.365671000 4.054109000  1 4.308571000 -2.449521000 5.483099000  6 2.754520000 -0.966107000 5.476149000  17 1.665391000 -1.848115000 6.571935000  17 1.779829000 -0.016075000 4.317834000  17 3.793292000 0.140228000 6.414439000  6 1.057056000 -3.318390000 -0.912209000  1 2.091804000 -3.670235000 -0.786230000  1 0.379615000 -4.071363000 -0.482032000  1 0.851867000 -3.260041000 -1.995332000  6 0.066413000 2.238943000 0.764968000  1 0.750550000 3.018468000 0.394046000  1 -0.961435000 2.626695000 0.684117000  1 0.282630000 2.087931000 1.835917000 |
|  | **TSEq_*trans***  E (Def2TZVPP): -2451.091885 A.U.  G_corr_ (Def2SVP): 0.227442 A.U.  6 -0.570698000 -1.383503000 -0.622771000  6 0.784603000 -1.946414000 -0.214602000  6 1.904614000 -0.931560000 -0.408722000  6 1.594817000 0.373140000 0.342758000  6 0.234832000 0.973856000 -0.047274000  6 -0.870545000 -0.079278000 0.131642000  1 2.863172000 -1.349471000 -0.063929000  1 1.023521000 -2.787221000 -1.048383000  1 -1.360923000 -2.126627000 -0.439013000  1 1.600902000 0.172406000 1.427355000  1 2.400624000 1.101511000 0.156179000  1 0.024023000 1.797260000 0.656174000  1 -0.984067000 -0.303649000 1.205685000  1 -1.838976000 0.323854000 -0.206373000  1 2.017978000 -0.718543000 -1.484499000  1 -0.566971000 -1.186487000 -1.707658000  16 0.440173000 -5.094494000 -1.495402000  8 1.361058000 -5.726734000 -0.580902000  8 -0.917246000 -4.778035000 -1.099424000  7 1.234781000 -3.779632000 -2.129715000  8 0.379309000 -6.065366000 -2.789752000  6 -0.813405000 -6.254340000 -3.523920000  1 -1.700340000 -6.031530000 -2.912694000  1 -0.830405000 -7.311679000 -3.821455000  6 -0.852799000 -5.396434000 -4.794042000  17 0.598698000 -5.674961000 -5.784003000  17 -0.964262000 -3.659665000 -4.389283000  17 -2.310599000 -5.878974000 -5.703633000  6 0.804920000 -2.674254000 1.115117000  1 0.017105000 -3.440256000 1.164765000  1 1.776668000 -3.159272000 1.290934000  1 0.631791000 -1.967608000 1.944437000  6 0.244452000 1.576430000 -1.453685000  1 -0.727265000 2.038534000 -1.689265000  1 1.016001000 2.357673000 -1.540890000  1 0.448387000 0.826148000 -2.233502000 |
|  | **INT1Ax_*trans***  E (Def2TZVPP): -2451.11165 A.U.  G_corr_ (Def2SVP): 0.225911 A.U.  6 -0.635478000 -1.435904000 -0.370167000  6 0.778393000 -1.927364000 -0.489848000  6 1.831576000 -0.895367000 -0.770231000  6 1.658472000 0.382412000 0.053968000  6 0.229713000 0.930405000 -0.006086000  6 -0.760253000 -0.146995000 0.447289000  1 2.834994000 -1.321145000 -0.609805000  1 1.054600000 -2.245948000 1.409741000  1 -1.281342000 -2.224915000 0.049685000  1 1.916655000 0.168787000 1.106664000  1 2.373904000 1.146517000 -0.290437000  1 0.005585000 1.167495000 -1.063859000  1 -0.575481000 -0.369444000 1.514482000  1 -1.792283000 0.234214000 0.384299000  1 1.780304000 -0.637531000 -1.850765000  1 -1.022071000 -1.248160000 -1.395784000  16 2.819724000 -2.890816000 2.513548000  8 2.912196000 -4.262503000 2.070317000  8 3.682143000 -1.866080000 1.961161000  7 1.226003000 -2.491196000 2.425883000  8 3.034404000 -2.936076000 4.109528000  6 3.725188000 -1.915566000 4.801862000  1 4.390088000 -1.353014000 4.130423000  1 4.324642000 -2.414839000 5.575164000  6 2.767371000 -0.938668000 5.493552000  17 1.630672000 -1.813657000 6.544295000  17 1.845140000 0.012819000 4.293533000  17 3.769882000 0.168629000 6.470329000  6 1.016331000 -3.333866000 -0.935966000  1 2.048825000 -3.660605000 -0.735411000  1 0.326408000 -4.042305000 -0.450148000  1 0.856582000 -3.430065000 -2.029480000  6 0.082119000 2.209916000 0.810602000  1 0.777615000 2.990269000 0.463342000  1 -0.939971000 2.614883000 0.742090000  1 0.295858000 2.023590000 1.876475000 |
|  | **INT1Eq_*trans***  E (Def2TZVPP): -2451.105808 A.U.  G_corr_ (Def2SVP): 0.225594 A.U.  6 -0.533942000 -1.422002000 -0.457300000  6 0.819333000 -1.857948000 0.031330000  6 1.923160000 -0.872222000 -0.231919000  6 1.554749000 0.509044000 0.349466000  6 0.191971000 1.014077000 -0.152159000  6 -0.892464000 -0.039730000 0.125521000  1 2.875233000 -1.220564000 0.197664000  1 1.193256000 -3.128540000 -1.375322000  1 -1.304699000 -2.161370000 -0.193977000  1 1.524233000 0.429848000 1.449604000  1 2.345050000 1.238471000 0.106923000  1 -0.062845000 1.911979000 0.436356000  1 -1.022311000 -0.142056000 1.216482000  1 -1.862193000 0.294997000 -0.278249000  1 2.084343000 -0.767365000 -1.320504000  1 -0.530177000 -1.351190000 -1.560735000  16 0.422704000 -5.152722000 -1.555897000  8 1.314964000 -5.851289000 -0.660505000  8 -0.919457000 -4.792632000 -1.145868000  7 1.271476000 -3.865952000 -2.131776000  8 0.325744000 -6.070497000 -2.876452000  6 -0.876099000 -6.203794000 -3.608079000  1 -1.752979000 -5.970662000 -2.986453000  1 -0.926708000 -7.251531000 -3.933350000  6 -0.893983000 -5.311114000 -4.854558000  17 0.546244000 -5.604302000 -5.856198000  17 -0.956499000 -3.582805000 -4.404955000  17 -2.366985000 -5.729071000 -5.772520000  6 0.917299000 -2.766512000 1.215989000  1 0.154439000 -3.559827000 1.187786000  1 1.910348000 -3.237985000 1.289589000  1 0.761230000 -2.206951000 2.161060000  6 0.231220000 1.436697000 -1.622275000  1 -0.744001000 1.836454000 -1.942308000  1 0.984933000 2.223222000 -1.785275000  1 0.479341000 0.600668000 -2.294881000 |
|  | **Norbornane**  E (Def2TZVPP): -273.9976119 A.U.  G_corr_ (Def2SVP): 0.148651 A.U.  6 -6.132221000 0.516161000 -0.643183000  6 -6.091212000 0.163000000 0.871025000  6 -4.764934000 -0.607376000 1.014186000  6 -3.600609000 0.388810000 0.856840000  6 -3.641538000 0.742829000 -0.657167000  6 -4.823971000 -0.094676000 -1.179909000  6 -4.703605000 -1.365713000 -0.322398000  1 -7.004976000 0.059060000 -1.134703000  1 -6.185233000 1.600039000 -0.828383000  1 -6.120694000 1.052731000 1.518656000  1 -6.943515000 -0.473844000 1.154235000  1 -4.704175000 -1.220740000 1.924371000  1 -2.644475000 -0.084471000 1.128943000  1 -3.723726000 1.269669000 1.505533000  1 -3.787593000 1.818319000 -0.841225000  1 -2.706276000 0.450965000 -1.159481000  1 -4.816844000 -0.241015000 -2.269347000  1 -5.540574000 -2.068133000 -0.463937000  1 -3.755208000 -1.905617000 -0.474056000 |
|  | **TSHAT_Norbornane_H^1^**  E (Def2TZVPP): -2410.539486 A.U.  G_corr_ (Def2SVP): 0.177588 A.U.  6 -5.583519000 0.261214000 -0.774559000  6 -6.122982000 -0.133066000 0.638227000  6 -4.813938000 -0.400773000 1.367684000  6 -4.074023000 0.887050000 1.699996000  6 -3.541628000 1.278024000 0.283647000  6 -4.052113000 0.135316000 -0.621226000  6 -3.933563000 -1.079000000 0.325781000  1 -5.956454000 -0.432631000 -1.542439000  1 -5.885838000 1.276935000 -1.069017000  1 -6.709123000 0.663221000 1.117361000  1 -6.748333000 -1.036810000 0.592396000  1 -5.045142000 -1.126910000 2.434843000  1 -3.248485000 0.704266000 2.401385000  1 -4.736378000 1.644291000 2.141539000  1 -3.910002000 2.260575000 -0.045988000  1 -2.442269000 1.315521000 0.279393000  1 -3.516079000 0.052655000 -1.576501000  1 -4.364173000 -2.003586000 -0.086414000  1 -2.907087000 -1.261405000 0.672991000  16 -3.903307000 -1.698921000 4.427577000  8 -3.928509000 -0.366040000 4.986091000  8 -3.746062000 -2.860606000 5.273682000  7 -5.264413000 -1.837248000 3.502298000  8 -2.736593000 -1.739551000 3.294871000  6 -1.697978000 -2.693714000 3.318374000  1 -1.655125000 -3.175115000 2.331161000  1 -1.873003000 -3.455805000 4.091675000  6 -0.359171000 -2.003185000 3.589783000  17 -0.385957000 -1.206229000 5.182170000  17 -0.019915000 -0.786469000 2.327737000  17 0.906197000 -3.259306000 3.560167000 |
|  | **TSHAT_Norbornane_H^2^**  E (Def2TZVPP): -2410.541324 A.U.  G_corr_ (Def2SVP): 0.17727 A.U.  6 -5.598276000 1.333458000 -0.632174000  6 -5.258354000 1.292834000 0.885514000  6 -4.726451000 -0.138055000 1.094381000  6 -3.342994000 -0.254771000 0.412371000  6 -3.686016000 -0.217239000 -1.107012000  6 -5.225651000 -0.079219000 -1.121588000  6 -5.560315000 -0.926668000 0.096347000  1 -6.668998000 1.522728000 -0.797765000  1 -5.039738000 2.109034000 -1.176135000  1 -4.512743000 2.046460000 1.178398000  1 -6.156491000 1.465080000 1.497959000  1 -4.744424000 -0.486458000 2.134866000  1 -2.852360000 -1.200385000 0.686326000  1 -2.677965000 0.564566000 0.723954000  1 -3.207700000 0.620780000 -1.635541000  1 -3.373711000 -1.145135000 -1.608414000  1 -5.698655000 -0.372015000 -2.066986000  1 -6.815054000 -0.800986000 0.411237000  1 -5.442610000 -2.018772000 0.036915000  16 -8.804243000 -1.143918000 -0.757967000  8 -8.542222000 -0.185798000 -1.802771000  8 -8.528704000 -2.556113000 -0.929155000  7 -8.102236000 -0.666590000 0.666545000  8 -10.366192000 -0.944235000 -0.421261000  6 -11.034299000 -1.874117000 0.412398000  1 -11.260414000 -1.396541000 1.376598000  1 -10.425850000 -2.775477000 0.578772000  6 -12.343707000 -2.292771000 -0.256621000  17 -12.016405000 -3.099317000 -1.810063000  17 -13.383619000 -0.871862000 -0.534545000  17 -13.163624000 -3.425925000 0.852579000 |
|  | **TSHAT_Norbornane_H^3^**  E (Def2TZVPP): -2410.543659 A.U.  G_corr_ (Def2SVP): 0.177398 A.U.  6 -6.822968000 0.840723000 -0.208562000  6 -6.423218000 0.379974000 1.194429000  6 -5.122170000 -0.384319000 1.005684000  6 -4.035669000 0.666150000 0.667244000  6 -4.404324000 1.104908000 -0.776136000  6 -5.674132000 0.287203000 -1.075925000  6 -5.364289000 -1.039990000 -0.364364000  1 -7.786706000 0.409997000 -0.520751000  1 -6.929617000 1.934743000 -0.269068000  1 -6.458254000 1.120983000 2.006428000  1 -7.224445000 -0.474951000 1.616975000  1 -4.859916000 -1.046794000 1.841232000  1 -3.041356000 0.196072000 0.706964000  1 -4.031211000 1.500659000 1.384217000  1 -4.581643000 2.187453000 -0.861712000  1 -3.602975000 0.846880000 -1.484815000  1 -5.925712000 0.218649000 -2.142638000  1 -6.213871000 -1.740784000 -0.362620000  1 -4.474652000 -1.551723000 -0.761898000  16 -9.473330000 -1.329947000 1.291773000  8 -9.240064000 -1.691287000 -0.089625000  8 -10.549292000 -1.934146000 2.045093000  7 -8.029321000 -1.530336000 2.076150000  8 -9.641867000 0.284978000 1.372542000  6 -10.808996000 0.891369000 1.885980000  1 -10.494121000 1.681965000 2.580529000  1 -11.437102000 0.162084000 2.418604000  6 -11.624762000 1.526664000 0.756352000  17 -12.180462000 0.277905000 -0.386209000  17 -10.643511000 2.731661000 -0.118835000  17 -13.029647000 2.329873000 1.506341000 |
|  | **TSHAT_Norbornane_H^4^**  E (Def2TZVPP): -2410.542353 A.U.  G_corr_ (Def2SVP): 0.178995 A.U.  6 -5.331483000 1.295332000 -0.240962000  6 -5.909815000 0.416216000 0.871569000  6 -4.992178000 -0.794237000 0.948722000  6 -3.620823000 -0.345897000 1.502027000  6 -3.026748000 0.505269000 0.346409000  6 -4.130200000 0.458844000 -0.725495000  6 -4.666484000 -0.972457000 -0.551636000  1 -6.077741000 1.428822000 -1.039723000  1 -5.040968000 2.299612000 0.101789000  1 -5.905495000 0.980021000 2.017101000  1 -6.987838000 0.204687000 0.816113000  1 -5.428083000 -1.657441000 1.467169000  1 -2.996683000 -1.225620000 1.717162000  1 -3.722959000 0.216132000 2.443245000  1 -2.796128000 1.537076000 0.644365000  1 -2.097169000 0.058761000 -0.037407000  1 -3.784580000 0.730815000 -1.731813000  1 -5.553522000 -1.187561000 -1.167221000  1 -3.908924000 -1.752138000 -0.728007000  16 -5.559472000 3.048153000 3.206453000  8 -6.505969000 3.706169000 2.337365000  8 -5.289797000 3.542674000 4.537782000  7 -5.976882000 1.443363000 3.285359000  8 -4.153994000 2.966075000 2.394810000  6 -2.915830000 3.238506000 3.017880000  1 -2.235055000 2.405006000 2.794206000  1 -3.031958000 3.341159000 4.106607000  6 -2.315929000 4.532922000 2.460900000  17 -3.373200000 5.918925000 2.826860000  17 -2.096525000 4.417367000 0.694231000  17 -0.729448000 4.757078000 3.245086000 |
|  | **Chloroadamantane**  E (Def2TZVPP): -850.3907147 A.U.  G_corr_ (Def2SVP): 0.202865 A.U.  6 -3.601575000 -0.193008000 -1.157042000  6 -2.911766000 -0.535924000 0.171882000  6 -3.905294000 -0.350844000 1.328539000  6 -5.113167000 -1.277100000 1.122814000  6 -5.802085000 -0.934113000 -0.206499000  6 -6.279856000 0.528706000 -0.172241000  6 -5.067398000 1.439492000 0.033853000  6 -4.079250000 1.269825000 -1.122422000  6 -4.383225000 1.111907000 1.363084000  6 -4.809229000 -1.119469000 -1.363698000  1 -2.890546000 -0.314828000 -1.989140000  1 -2.032253000 0.112259000 0.320570000  1 -2.547746000 -1.576329000 0.150320000  1 -3.412830000 -0.586131000 2.285063000  1 -5.824029000 -1.164032000 1.958083000  1 -4.784745000 -2.329467000 1.116736000  1 -6.674813000 -1.589217000 -0.354744000  1 -7.000558000 0.680844000 0.646695000  1 -6.785173000 0.792506000 -1.114754000  1 -3.225720000 1.951954000 -0.983002000  1 -4.570061000 1.538809000 -2.071113000  1 -5.091764000 1.267357000 2.191963000  1 -3.532085000 1.793376000 1.519161000  1 -4.475490000 -2.169178000 -1.409705000  1 -5.300623000 -0.893049000 -2.324458000  17 -5.635722000 3.180167000 0.074998000 |
|  | **TSHAT_Chloroadamantane_H^1^**  E (Def2TZVPP): -2986.938474 A.U.  G_corr_ (Def2SVP): 0.233094 A.U.  6 -4.304907000 -0.083829000 -1.516297000  6 -3.388382000 -0.340448000 -0.317468000  6 -4.252027000 -0.616301000 0.925093000  6 -5.129487000 -1.864338000 0.674697000  6 -6.000233000 -1.552623000 -0.527899000  6 -6.945542000 -0.395674000 -0.261288000  6 -6.064507000 0.848829000 -0.007213000  6 -5.202519000 1.127127000 -1.250321000  6 -5.156853000 0.592195000 1.204105000  6 -5.171864000 -1.327563000 -1.784866000  1 -2.747383000 0.538723000 -0.149280000  1 -2.730190000 -1.197312000 -0.530008000  1 -3.593819000 -0.805104000 1.786860000  1 -5.752277000 -2.071900000 1.557197000  1 -4.496049000 -2.744750000 0.483373000  1 -6.678452000 -2.570475000 -0.780334000  1 -7.575703000 -0.602688000 0.615800000  1 -7.609903000 -0.226367000 -1.122265000  1 -6.714448000 1.716307000 0.182901000  1 -4.578098000 2.019851000 -1.091151000  1 -5.839749000 1.317602000 -2.127814000  1 -5.767941000 0.407714000 2.101969000  1 -4.540940000 1.483118000 1.408005000  1 -4.528269000 -2.194604000 -1.996364000  1 -5.818396000 -1.152791000 -2.657777000  16 -8.798944000 -3.572576000 -0.183721000  8 -9.564394000 -2.469732000 -0.718989000  8 -9.403142000 -4.872078000 0.009430000  7 -7.436225000 -3.695354000 -1.116247000  8 -8.148412000 -3.104912000 1.232997000  6 -8.413862000 -3.785426000 2.440775000  1 -7.452590000 -3.986452000 2.934050000  1 -8.937127000 -4.735188000 2.257586000  6 -9.269850000 -2.913196000 3.362437000  17 -10.841434000 -2.565507000 2.601481000  17 -8.433985000 -1.375908000 3.720264000  17 -9.515860000 -3.820274000 4.877111000  17 -3.282115000 0.240902000 -2.991985000 |
|  | **TSHAT_Chloroadamantane_H^2^**  E (Def2TZVPP): -2986.936881 A.U.  G_corr_ (Def2SVP): 0.234015 A.U.  6 -4.475667000 0.035131000 -1.657458000  6 -3.524679000 -0.400047000 -0.532965000  6 -4.325225000 -0.624901000 0.758205000  6 -5.372981000 -1.728743000 0.528590000  6 -6.316356000 -1.290601000 -0.595528000  6 -6.999443000 0.011404000 -0.234682000  6 -5.987450000 1.112630000 0.035770000  6 -5.187116000 1.337814000 -1.266653000  6 -5.030084000 0.678839000 1.157647000  6 -5.523124000 -1.063897000 -1.895766000  1 -3.909284000 0.189754000 -2.588722000  1 -2.755833000 0.372423000 -0.369474000  1 -2.999147000 -1.325441000 -0.820076000  1 -3.649701000 -0.943534000 1.566715000  1 -5.955130000 -1.916115000 1.444357000  1 -4.885600000 -2.674253000 0.245969000  1 -7.615223000 -0.135331000 0.875720000  1 -7.824049000 0.286822000 -0.908492000  1 -6.517719000 2.035309000 0.314982000  1 -4.454592000 2.144821000 -1.104449000  1 -5.860784000 1.663910000 -2.075143000  1 -5.587539000 0.540821000 2.097818000  1 -4.287986000 1.473485000 1.334395000  1 -5.040927000 -2.010931000 -2.184666000  1 -6.213156000 -0.777781000 -2.704366000  16 -9.274416000 -1.339655000 2.155400000  8 -10.202604000 -1.271085000 1.058212000  8 -8.543953000 -2.546393000 2.486628000  7 -8.162298000 -0.107754000 2.110838000  8 -10.147304000 -0.885562000 3.435920000  6 -9.623609000 -1.046132000 4.740827000  1 -9.398893000 -0.056509000 5.164339000  1 -8.712621000 -1.663142000 4.736550000  6 -10.670299000 -1.736144000 5.616470000  17 -11.045047000 -3.354823000 4.976920000  17 -12.163867000 -0.764728000 5.681715000  17 -9.979666000 -1.879044000 7.256298000  17 -7.564453000 -2.581482000 -0.886142000 |

#

# 12. References:

1. a) Bess, E.N., DeLuca, R.J., Tindall, D.J., Oderinde, M.S., Roizen, J.L., Du Bois, J., and Sigman, M.S. (2014). Analyzing Site Selectivity in Rh_2_(esp)_2_ -Catalyzed Intermolecular C–H Amination Reactions. J. Am. Chem. Soc. *136*, 5783–5789. <https://doi.org/10.1021/ja5015508>; Jennifer, L. R, David, N. Z., and Du Bois, J. (2013). Selective Intermolecular Amination of C-H Bonds at Tertiary Carbon Centers. Angew. Chem. Int. Ed. *52*, 11343 –11346. https://doi.org/10.1002/anie.201304238.
2. Alderson, Juliet M., Alicia M. Phelps, Ryan J. Scamp, Nicholas S. Dolan, and Jennifer M. Schomaker (2014). “Ligand-Controlled, Tunable Silver-Catalyzed C–H Amination.” J. Am. Chem. Soc. 136 (48): 16720–23. <https://doi.org/10.1021/ja5094309>.
3. Ghosh, Subrata K., Mengnan Hu, and Robert J. Comito (2021). “One‐Pot Synthesis of Primary and Secondary Aliphatic Amines via Mild and Selective Sp3 C−H Imination.” Chem. Eur. J. 27 (70): 17601–8. <https://doi.org/10.1002/chem.202102627>.
4. Clark, Joseph R., Kaibo Feng, Anasheh Sookezian, and M. Christina White (2018). “Manganese-Catalysed Benzylic C(Sp3)–H Amination for Late-Stage Functionalization.” Nat. Chem. 10 (6): 583–91. <https://doi.org/10.1038/s41557-018-0020-0>.
5. Fiori, Kristin Williams, and J. Du Bois (2007). “Catalytic Intermolecular Amination of C−H Bonds: Method Development and Mechanistic Insights.” J. Am. Chem. Soc. 129 (3): 562–68. <https://doi.org/10.1021/ja0650450>.
6. Fei, Jun, Zhen Wang, Zheren Cai, Hao Sun, and Xu Cheng (2015). “Synthesis of α‐Tertiary Amine Derivatives by Intermolecular Hydroamination of Unfunctionalized Alkenes with Sulfamates under Trifluoromethanesulfonic Acid Catalysis.” Adv. Synth. Catal. 357 (18): 4063–68. <https://doi.org/10.1002/adsc.201500646>.
7. Combee, Logan A., Balaram Raya, Daoyong Wang, and Michael K. Hilinski (2018). “Organocatalytic Nitrenoid Transfer: Metal-Free Selective Intermolecular C(Sp3)–H Amination Catalyzed by an Iminium Salt.” Chem. Sci. 9 (4): 935–39. <https://doi.org/10.1039/C7SC03968A>.
8. Lu, Xunbo, Yufeng Shi, and Fangrui Zhong (2018). “Rhodium-Catalyzed Intermolecular C(Sp3)–H Amination in a Purely Aqueous System.” Green Chem. 20 (1): 113–17. <https://doi.org/10.1039/C7GC03149A>.
9. Heider, Christian, Dominik Pietschmann, Dieter Vogt, and Thomas Seidensticker (2022). “Selective Synthesis of Primary Amines by Kinetic‐based Optimization of the Ruthenium‐Xantphos Catalysed Amination of Alcohols with Ammonia.” ChemCatChem 14 (18): e202200788. <https://doi.org/10.1002/cctc.202200788>.
10. Kobayashi, Shoji, Ryo Yamaguchi, Fumiya Yamamoto, Jun Komori, Hotaka Sakamoto, Takahiro Kasashima, Louis Adriaenssens, and Martin J. Lear (2023). “One‐Pot Conversion of Benzyl Alcohols to N‐Protected Anilines and Alkyl Alcohols to Carbamoyl Azides.” Eur. J. Org. Chem. 2023 (47): e202300786. <https://doi.org/10.1002/ejoc.202300786>.
11. Satheesh, Vanaparthi, Indunil Alahakoon, Kendra K. Shrestha, Livina C. Iheme, Michal Marszewski, and Michael C. Young (2024). “Self‐Supported Heterogeneous Dirhodium(II) Catalyst for Nitrene and Carbene Transfer Reactions.” Eur. J. Org. Chem. 2024 (8): e202301114. <https://doi.org/10.1002/ejoc.202301114>.
12. Liu, Wei, Dayou Zhong, Cheng-Long Yu, Yan Zhang, Di Wu, Ya-Lan Feng, Hengjiang Cong, Xiuqiang Lu, and Wen-Bo Liu (2019). “Iron-Catalyzed Intramolecular Amination of Aliphatic C–H Bonds of Sulfamate Esters with High Reactivity and Chemoselectivity.” Org. Lett. 21 (8): 2673–78. <https://doi.org/10.1021/acs.orglett.9b00660>.
13. Espino, Christine G., Paul M. Wehn, Jessica Chow, and J. Du Bois (2001). “Synthesis of 1,3-Difunctionalized Amine Derivatives through Selective C−H Bond Oxidation.” J. Am. Chem. Soc. 123 (28): 6935–36. <https://doi.org/10.1021/ja011033x>.
14. Alderson, Juliet M., Alicia M. Phelps, Ryan J. Scamp, Nicholas S. Dolan, and Jennifer M. Schomaker (2014). “Ligand-Controlled, Tunable Silver-Catalyzed C–H Amination.” J. Am. Chem. Soc. 136 (48): 16720–23. <https://doi.org/10.1021/ja5094309>.
15. Paradine, Shauna M., Jennifer R. Griffin, Jinpeng Zhao, Aaron L. Petronico, Shannon M. Miller, and M. Christina White (2015). “A Manganese Catalyst for Highly Reactive yet Chemoselective Intramolecular C(Sp3)–H Amination.” Nat. Chem. 7 (12): 987–94. <https://doi.org/10.1038/nchem.2366>.
16. Lakowicz, Joseph R., ed. (1983). *Principles of Fluorescence Spectroscopy*. New York: Plenum Press, 52–93.
17. Montalti, Marco, Andrea Credi, Lucia Prodi, and Margherita T. Gandolfi (2006). *Handbook of Photochemistry*. 3rd ed. Boca Raton, FL: CRC Press, Taylor & Francis Group.
18. Frisch, Michael J., G. W. Trucks, H. B. Schlegel, G. E. Scuseria, M. A. Robb, J. R. Cheeseman, G. Scalmani, V. Barone, G. A. Petersson, H. Nakatsuji, X. Li, M. Caricato, A. V. Marenich, J. Bloino, B. G. Janesko, R. Gomperts, B. Mennucci, H. P. Hratchian, J. V. Ortiz, A. F. Izmaylov, J. L. Sonnenberg, D. Williams-Young, F. Ding, F. Lipparini, F. Egidi, J. Goings, B. Peng, A. Petrone, T. Henderson, D. Ranasinghe, V. G. Zakrzewski, J. Gao, N. Rega, G. Zheng, W. Liang, M. Hada, M. Ehara, K. Toyota, R. Fukuda, J. Hasegawa, M. Ishida, T. Nakajima, Y. Honda, O. Kitao, H. Nakai, T. Vreven, K. Throssell, J. A. Montgomery Jr., J. E. Peralta, F. Ogliaro, M. J. Bearpark, J. J. Heyd, E. N. Brothers, K. N. Kudin, V. N. Staroverov, T. A. Keith, R. Kobayashi, J. Normand, K. Raghavachari, A. P. Rendell, J. C. Burant, S. S. Iyengar, J. Tomasi, M. Cossi, J. M. Millam, M. Klene, C. Adamo, R. Cammi, J. W. Ochterski, R. L. Martin, K. Morokuma, O. Farkas, J. B. Foresman, and D. J. Fox (2016). Gaussian 16, Revision C.01. Gaussian, Inc., Wallingford CT.
19. Chai, Jeng-Da, and Martin Head-Gordon (2008). “Long-Range Corrected Hybrid Density Functionals with Damped Atom–Atom Dispersion Corrections.” Physical Chemistry Chemical Physics 10 (44): 6615–20. <https://doi.org/10.1039/B810189B>.
20. Weigend, Florian, and Reinhart Ahlrichs (2005). “Balanced Basis Sets of Split Valence, Triple Zeta Valence and Quadruple Zeta Valence Quality for H to Rn: Design and Assessment of Accuracy.” Physical Chemistry Chemical Physics 7 (18): 3297–3305. <https://doi.org/10.1039/B508541A>.
21. Marenich, Aleksandr V., Christopher J. Cramer, and Donald G. Truhlar (2009). “Universal Solvation Model Based on Solute Electron Density and a Continuum Model of the Solvent Defined by the Bulk Dielectric Constant and Atomic Surface Tensions.” Journal of Physical Chemistry B 113 (18): 6378–96. <https://doi.org/10.1021/jp810292n>.
22. Legault, Cyril Y. (2020). CYLview 1.0. Université de Sherbrooke. http://www.cylview.org.
23. Marcus, Rudolph A. (1956). “On the Theory of Oxidation-Reduction Reactions Involving Electron Transfer. I.” Journal of Chemical Physics 24: 966–78.
24. de Aguirre, Adrián, Ignacio Funes-Ardoiz, and Feliu Maseras (2019). “Computational Characterization of Single-Electron Transfer Steps in Water Oxidation.” Inorganics 7 (3): 32. <https://doi.org/10.3390/inorganics7030032>.
25. Solé-Daura, Albert, and Feliu Maseras (2024). “Straightforward Computational Determination of Energy-Transfer Kinetics through the Application of the Marcus Theory.” Chemical Science 15: 13650–58. <https://doi.org/10.1039/D4SC02057H>.

# 13. Spectral data:

^1^H NMR Spectrum of **2,2,2-trifluoroethyl sulfamate 1** (400 MHz, CDCl_3_)

^13^C NMR Spectrum of **2,2,2-trifluoroethyl sulfamate 1** (101 MHz, CDCl_3_)

^19^F NMR Spectrum of **2,2,2-trifluoroethyl sulfamate 1** (376 MHz, CDCl_3_)

^1^H NMR Spectrum of **2,2,2-trichloroethyl sulfamate 2** (400 MHz, CDCl_3_)

^13^C NMR Spectrum of **2,2,2-trichloroethyl sulfamate 2** (101 MHz, CDCl_3_)

^1^H NMR Spectrum of **2a** (400 MHz, CD_3_OD)

^13^C NMR Spectrum of **2a** (101 MHz, CD_3_OD)

^19^F NMR Spectrum of **2a** (376 MHz, CD_3_OD)

^1^H NMR Spectrum of **2b** (400 MHz, CD_3_OD)

^13^C NMR Spectrum of **2b** (101 MHz, CD_3_OD)

^1^H NMR Spectrum of **3a** (400 MHz, CDCl_3_)

^13^C NMR Spectrum of **3a** (101 MHz, CDCl_3_)

^19^F NMR Spectrum of **3a** (376 MHz, CDCl_3_)

^1^H NMR Spectrum of **3a’** (400 MHz, CDCl_3_)

^13^C NMR Spectrum of **3a’** (101 MHz, CDCl_3_)

^1^H NMR Spectrum of **3b** (400 MHz, CDCl_3_)

^13^C NMR Spectrum of **3b** (101 MHz, CDCl_3_)

^19^F NMR Spectrum of **3b** (376 MHz, CDCl_3_)

^1^H NMR Spectrum of **3b’** (400 MHz, CDCl_3_)

^13^C NMR Spectrum of **3b’** (101 MHz, CDCl_3_)

^1^H NMR Spectrum of **3c** (400 MHz, CDCl_3_)

^13^C NMR Spectrum of **3c** (101 MHz, CDCl_3_)

^19^F NMR Spectrum of **3c** (376 MHz, CDCl_3_)

^1^H NMR Spectrum of **3c’** (400 MHz, CDCl_3_)

^13^C NMR Spectrum of **3c’** (101 MHz, CDCl_3_)

^1^H NMR Spectrum of **3d** (400 MHz, CDCl_3_)

^13^C NMR Spectrum of **3d** (101 MHz, CDCl_3_)

^19^F NMR Spectrum of **3d** (376 MHz, CDCl_3_)

^1^H NMR Spectrum of **3d’** (400 MHz, CDCl_3_)

^13^C NMR Spectrum of **3d’** (101 MHz, CDCl_3_)

^1^H NMR Spectrum of **3e** (400 MHz, CDCl_3_)

^13^C NMR Spectrum of **3e** (400 MHz, CDCl_3_)

COSY spectrum of **3e**

HSQC spectrum of **3e**

^1^H NMR Spectrum of **3f** (400 MHz, CDCl_3_)

^13^C NMR Spectrum of **3f** (101 MHz, CDCl_3_)

^19^F NMR Spectrum of **3f** (376 MHz, CDCl_3_)

COSY spectrum of **3f**

HSQC spectrum of **3f**

^1^H NMR Spectrum of **3f’** (400 MHz, CDCl_3_)

^13^C NMR Spectrum of **3f’** (101 MHz, CDCl_3_)

COSY spectrum of **3f’**

HSQC spectrum of **3f’**

^1^H NMR Spectrum of **3g** (400 MHz, CDCl_3_)

^13^C NMR Spectrum of **3g** (101 MHz, CDCl_3_)

^19^F NMR Spectrum of **3g** (376 MHz, CDCl_3_)

COSY spectrum of **3g**

HSQC spectrum of **3g**

^1^H NMR Spectrum of **3h** (400 MHz, CDCl_3_)

^13^C NMR Spectrum of **3h** (101 MHz, CDCl_3_)

^19^F NMR Spectrum of **3h** (376 MHz, CDCl_3_)

^1^H NMR Spectrum of **3i** (400 MHz, CDCl_3_)

^13^C NMR Spectrum of **3i** (101 MHz, CDCl_3_)

^19^F NMR Spectrum of **3i** (376 MHz, CDCl_3_)

^1^H NMR Spectrum of **3j** (400 MHz, CDCl_3_)

^13^C NMR Spectrum of **3j** (101 MHz, CDCl_3_)

^19^F NMR Spectrum of **3j** (376 MHz, CDCl_3_)

^1^H NMR Spectrum of **3k** (400 MHz, CDCl_3_)

^13^C NMR Spectrum of **3k** (101 MHz, CDCl_3_)

^19^F NMR Spectrum of **3k** (376 MHz, CDCl_3_)

^1^H NMR Spectrum of **3****l** (400 MHz, CDCl_3_)

^13^C NMR Spectrum of **3l** (101 MHz, CDCl_3_)

^19^F NMR Spectrum of **3l** (376 MHz, CDCl_3_)

^1^H NMR Spectrum of **3****l’** (400 MHz, CDCl_3_)

^13^C NMR Spectrum of **3l’** (101 MHz, CDCl_3_)

^1^H NMR Spectrum of **3****m** (400 MHz, CDCl_3_)

^13^C NMR Spectrum of **3m** (101 MHz, CDCl_3_)

^19^F NMR Spectrum of **3m** (376 MHz, CDCl_3_)

^1^H NMR Spectrum of **3****m’** (400 MHz, CDCl_3_)

^13^C NMR Spectrum of **3m’** (101 MHz, CDCl_3_)

^1^H NMR Spectrum of **3n** (400 MHz, CDCl_3_)

^13^C NMR Spectrum of **3n** (101 MHz, CDCl_3_)

^19^F NMR Spectrum of **3n** (376 MHz, CDCl_3_)

^1^H NMR Spectrum of **3o** (400 MHz, CDCl_3_)

^13^C NMR Spectrum of **3o** (101 MHz, CDCl_3_)

^19^F NMR Spectrum of **3o** (376 MHz, CDCl_3_)

^1^H NMR Spectrum of **3o’** (400 MHz, CDCl_3_)

^13^C NMR Spectrum of **3o’** (101 MHz, CDCl_3_)

^1^H NMR Spectrum of **3****p** (400 MHz, CDCl_3_)

^13^C NMR Spectrum of **3p** (101 MHz, CDCl_3_)

^19^F NMR Spectrum of **3p** (376 MHz, CDCl_3_)

^1^H NMR Spectrum **3****q** (400 MHz, CDCl_3_)

^13^C NMR Spectrum of **3q** (101 MHz, CDCl_3_)

^19^F NMR Spectrum of **3q** (376 MHz, CDCl_3_)

^1^H NMR Spectrum of **3****r** (400 MHz, CDCl_3_)

^13^C NMR Spectrum of **3r** (101 MHz, CDCl_3_)

^19^F NMR Spectrum of **3r** (376 MHz, CDCl_3_)

^1^H NMR Spectrum of **3s** (400 MHz, CDCl_3_)

^13^C NMR Spectrum of **3s** (101 MHz, CDCl_3_)

^19^F NMR Spectrum of **3s** (376 MHz, CDCl_3_)

^1^H NMR Spectrum of **3****t** (400 MHz, CDCl_3_)

^13^C NMR Spectrum of **3t** (101 MHz, CDCl_3_)

^19^F NMR Spectrum of **3t** (376 MHz, CDCl_3_)

^1^H NMR Spectrum of **3****t’** (400 MHz, CDCl_3_)

^13^C NMR Spectrum of **3t’** (101 MHz, CDCl_3_)

^1^H NMR Spectrum of **3u** (400 MHz, CDCl_3_)

^13^C NMR Spectrum of **3u** (101 MHz, CDCl_3_)

^19^F NMR Spectrum of **3u** (376 MHz, CDCl_3_)

^1^H NMR Spectrum of **3v** (400 MHz, CDCl_3_)

^13^C NMR Spectrum of **3v** (101 MHz, CDCl_3_)

^19^F NMR Spectrum of **3v** (376 MHz, CDCl_3_)

^1^H NMR Spectrum of **5a** (400 MHz, CDCl_3_)

^13^C NMR Spectrum of **5a** (101 MHz, CDCl_3_)

^1^H NMR Spectrum of **5b** (400 MHz, CDCl_3_)

^13^C NMR Spectrum of **5b** (101 MHz, CDCl_3_)

^1^H NMR Spectrum of **5c** (400 MHz, CDCl_3_)

^13^C NMR Spectrum of **5c** (101 MHz, CDCl_3_)

^1^H NMR Spectrum of **5d** (400 MHz, CDCl_3_)

^13^C NMR Spectrum of **5d** (101 MHz, CDCl_3_)

^1^H NMR Spectrum of **5****e** (400 MHz, CDCl_3_)

^13^C NMR Spectrum of **5e** (101 MHz, CDCl_3_)

^1^H NMR Spectrum of **5****e’** (400 MHz, CDCl_3_)

^13^C NMR Spectrum of **5e’** (101 MHz, CDCl_3_)

^1^H NMR Spectrum of **5f, 5f’** (400 MHz, CDCl_3_)

^13^C NMR Spectrum of **5f, 5f’** (101 MHz, CDCl_3_)

^1^H NMR Spectrum of **5g** (400 MHz, CDCl_3_)

^13^C NMR Spectrum of **5g** (101 MHz, CDCl_3_)

^1^H NMR Spectrum of **5g’** (400 MHz, CDCl_3_)

^13^C NMR Spectrum of **5g’** (101 MHz, CDCl_3_)

^1^H NMR Spectrum of **6** (400 MHz, CDCl_3_)

^13^C NMR Spectrum of **6** (101 MHz, CDCl_3_)

^1^H NMR Spectrum of **7** (400 MHz, CDCl_3_)

^13^C NMR Spectrum of **7** (101 MHz, CDCl_3_)

^1^H NMR Spectrum of **8** (400 MHz, CDCl_3_)

^13^C NMR Spectrum of **8** (101 MHz, CDCl_3_)

^1^H NMR Spectrum of **9** (400 MHz, CDCl_3_)

^13^C NMR Spectrum of **9** (101 MHz, CDCl_3_)

^19^F NMR Spectrum of **9** (376 MHz, CDCl_3_)

^1^H NMR Spectrum of **10** (400 MHz, CDCl_3_)

^13^C NMR Spectrum of **10** (101 MHz, CDCl_3_)

^1^H NMR Spectrum of **5h (Rad****ical Clock****)** (400 MHz, CDCl_3_)

^13^C NMR Spectrum of **5h** **(Radical Clock)** (101 MHz, CDCl_3_)

Crude-^1^H NMR Spectrum of **Radical Clock experiment** (400 MHz, CDCl_3_)
